# Estimates of the global, regional, and national burden of atrial fibrillation in older adults from 1990 to 2019: insights from the Global Burden of Disease study 2019

**DOI:** 10.3389/fpubh.2023.1137230

**Published:** 2023-06-12

**Authors:** Min Jiao, Chenglin Liu, Yongwen Liu, Yan Wang, Qianqian Gao, Anning Ma

**Affiliations:** ^1^School of Public Health, Weifang Medical University, Weifang, China; ^2^Department of Cardiology, Affiliated Hospital of Jining Medical University, Jining, China; ^3^School of Management, Weifang Medical University, Weifang, China

**Keywords:** atrial fibrillation, aging, Global Burden of Disease, risk factors, older adults

## Abstract

**Background:**

Atrial fibrill ation (AF) is a predominant public health concern in older adults. Therefore, this study aimed to explore the global, regional, and national burden of AF in older adults aged 60–89 between 1990 and 2019.

**Methods:**

The morbidity, mortality, disability-adjusted life years (DALYs), and age-standardized rates of AF were refined from the Global Burden of Diseases study 2019. The epidemiological characteristics were assessed based on numerical values, age-standardized rates per 100,000 person-years, and estimated annual percentage changes (EAPC).

**Results:**

Globally, a total of 33.31 million AF cases, 219.4 thousand deaths, and 65.80 million DALYs were documented in 2019. There were no appreciable changes in EAPC from 1990 to 2019. The disease burden of AF differed significantly across different territories and countries. At the national level, China exhibited the highest number of incident cases [818,493 (562,871–1,128,695)], deaths [39,970 (33,722–46,387)], and DALYs [1,383,674 (1,047,540–1,802,516)]. At the global level, high body mass index (BMI) and high systolic blood pressure (SBP) were two predominant risk factors contributing to the proportion of AF-related deaths.

**Conclusion:**

AF in older adults remains a major public health concern worldwide. The burden of AF varies widely at both national and regional levels. From 1990 to 2019, the cases of incidences, deaths, and DALYs have shown a global increase. The ASIR, ASMR, and ASDR have declined in the high-moderate and high SDI regions; however, the burden of AF increased promptly in the lower SDI regions. Special attention should be paid to the main risk factors for high-risk individuals with AF, which can help control systolic blood pressure and body mass index within normal limits. Over all, it is necessary to illustrate the features of the global AF burden and develop more effective and targeted prevention and treatment strategies.

## Introduction

Atrial fibrillation (AF) is a common cardiac arrhythmia (1), affecting more than 33 million people. Globally, it is a primary cause of coronary heart disease and mortality (2). Relevant research indicates that the incidence and prevalence of AF have been going up in Europe and North America from the 1970's to the early twenty-first century (3). The prevalence of AF is expected to rise in the next 30–50 years (4, 5). Hence, AF has become a worldwide public health problem and strained the healthcare system. Understanding the pattern of AF incidence and the temporal trends can facilitate the development of more well-directed preventive measures.

The Global Burden of Disease (GBD) study estimates the AF burden in 204 countries and territories worldwide, providing an excellent opportunity to comprehend the picture of AF (6). Recent studies have assessed the prevalence, disability-adjusted life years (DALYs), and AF-related mortality in all age groups using the data derived from the GBD (7, 8). However, of all cardiovascular diseases, AF commonly causes heart failure (9), imposing a substantial disease burden, especially on older adults. The overall health of older adults declines with age, and multimorbidity is prevalent, especially in cardiovascular disease. Therefore, it is necessary to pay particular attention to older adults. A cohort study found a significant increase in the prevalence of AF in older adults over 60 years of age (10). In addition, the proportion of the older population over 90 years old is only 2%. Therefore, we selected 60–89-year-olds as the research subjects. Based on the GBD Study 2019 data, the purpose of this study was to explore the burden of AF and its risk factors among older adults aged 60–89 years at global, regional, and national levels from 1990 to 2019. Our outcomes can act as a crucial supplement and expansion to previous research while also contributing to the formulation of AF prevention measures targeting various countries.

## Methods

### Data sources

We collected corresponding data from the Global Health Data Exchange (GHDx) query tool (http://ghdx.healthdata.org/gbd-results-tool) (11), an online tool of the Global Burden of Diseases (GBD), Etiologies, Injuries, and Risk Factors Study. The quality of statistics has been recognized internationally, and the approaches employed in the GBD have been illustrated elsewhere. The GBD 2019 database consisted of epidemiological data (i.e., deaths, prevalence, incidence, and DALYs) for 204 countries and territories worldwide. Then, these countries and territories were divided into five regions in accordance with the sociodemographic index (SDI) (2). The regions ranged from high to low as follows: high SDI, high-middle SDI, middle SDI, low-middle SDI, and low SDI.

Furthermore, the world was geographically divided into 21 regions, such as Australasia and Eastern Europe. The Human Development Index (HDI) data were available for extraction from Human Development Reports (http://hdr.undp.org/en/data). It was a composite measure index of three elementary aspects of human development: life expectancy, education, and per capita income. In our research, HDI was assembled and utilized to match GBD data. The secondary analysis data in the present article did not require ethical agreement or approval from the ethics committee or the institutional review board.

### Statistical analysis

The burden of AF was expressed in terms of number, age-standardized incidence rates (ASIRs), age-standardized mortality rates (ASMRs), age-standardized DALY rates (ASDRs), and estimated annual percentage change (EAPC) with a confidence interval of 95%. The ASRs were counted using a standardized global aging structure, which is necessary when comparing populations in different locations or sample groups over a period of time (12).

In the present study, we calculated the EAPC in the age-standardized rates of AF to further assess the burden of the disease. EAPC was a generalization and extensively used weighing measure for ASR's tendency within a specified interval. It was calculated using the referring regression line: Y = α + βX + ε, where Y referred to ln (age-standardized rate), X presented the calendar year, β influenced the active or passive trends in ASR and ε meant error value. The EAPC can be given by the following formula: 100 × [exp (β) – 1], and its 95% confidence interval (CI) could be obtained from the linear regression model (12). There are three situations: (1) if the 95% CI and EAPC assessment were both <0, the ASRs were regarded as a descending trend, (2) if the 95% CI and EAPC were both >0, the ASRs were regarded as an increasing trend. (3) If not, the ASRs were regarded as a steady trend. The statistical analysis in the present study was accomplished using the R program (Version 4.1.2). If the *p*-value was <0.05, it was deemed to be statistically significant.

## Results

### Global burden of AF

**Figure 1:**
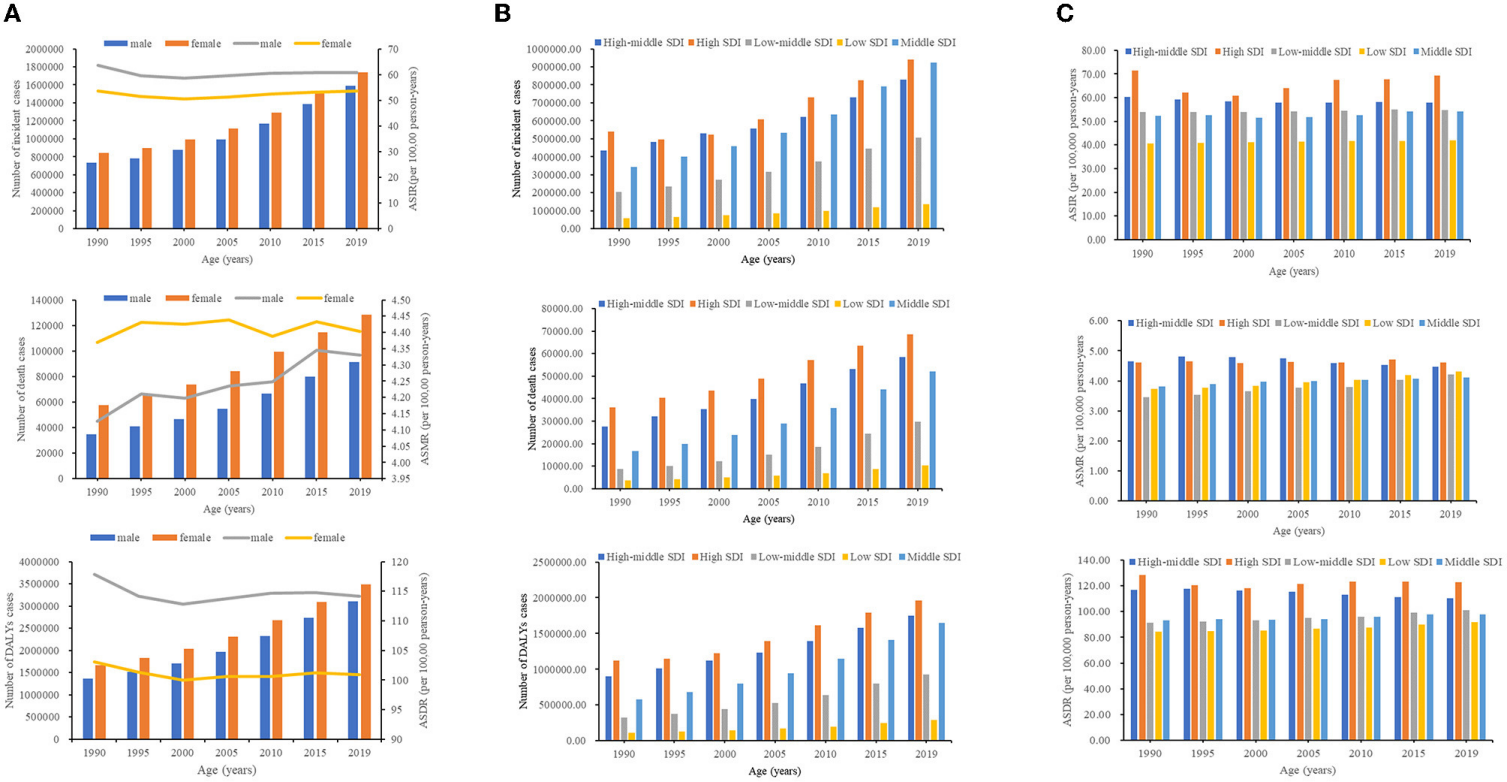
The number of cases and ASRs of AF from 1990 to 2019. **(A)** The number of cases of AF caused by global, from 1990 to 2019. **(B)** The number of cases of AF caused by SDI regions from 1990 to 2019. **(C)** The ASRs of AF caused by SDI regions from 1990 to 2019. SDI, sociodemographic index.

At the global level, a total of 3,331,144 (2,342,510–4,489,654) cases of AF were reported in 2019. The morbidity in both sexes increased by 1.1% from 1,583,024 (1,094,531–2,166,439) in 1990 to 3,331,144 (2,342,510–4,489,654) in 2019. Moreover, the ASIR fluctuated slightly by 0.05% from 58.54 (44.92–74.24) to 57.09 (44.07–71.9) during the past 30 years. Similarly, the mortality rate increased by 1.36% from 92,801 (82,995–111,456) in 1990 to 219,437 (190,526–249,642) in 2019. The ASMR demonstrates a mild increase of 0.04%, from 4.29 (3.73–5.09) in 1990 to 4.38 (3.7–5.05) in 2019 per 100,000 population. AF caused 6,579,978 (5,241,696–8,321,653) DALYs in 2019 worldwide, which was a 1.16% increase from 3,044,717 (2,363,647–3,900,467) in 1990. With regard to age-standardized rates, in 1990, the ASDR of AF was 110 (87.66–139.16) per 100,000 population, which slightly decreased to 107.13 (86.18–133.73) in 2019 (Table 1, Figure 1A).

**Table 1:** Incident cases, death, and DALYs of cardiomyopathy and myocarditis and ASRs per 10,000 population from 1990 and 2019 by Global Burden of Disease.

**Characteristics**	**1990**		**2019**		**1990–2019**	**1990–2019**
	**Incidence cases No. (95% UI)**	**ASIR per 100,000 No. (95% UI)**	**Incidence cases No. (95% UI)**	**ASIR per 100,000 No. (95% UI)**	**Percentage change No. (95% CI)**	**EAPC No. (95% CI)**
**Overall**	1,583,023.57 (1,094,531.35–2,166,438.85)	58.54 (44.92–74.24)	3,331,144.11 (2,342,509.66–4,489,654.42)	57.09 (44.07–71.9)	1.1% (1.06–1.16)	0.05 (−0.02–0.13)
**Sociodemographic index**						
Low SDI	59,083.69 (40,267.86–81,934.91)	40.67 (30.92–52.06)	135,864.94 (92,489.13–186,062.1)	41.97 (31.77–53.69)	1.19% (1.14–1.26)	0.12 (0.11–0.12)
Low-middle SDI	202,876.21 (138,996.1–280,233.52)	53.96 (41.04–68.62)	505,077.85 (346,414.8–690,949.01)	54.72 (41.51–69.76)	1.48% (1.4–1.57)	0.08 (0.06–0.09)
Middle SDI	342,872.23 (233,718.56–470,907.06)	52.39 (39.69–66.74)	922,124.53 (632,539.34–1,262,914.42)	54.27 (41.16–69.05)	1.57% (1.52–1.63)	0.14 (0.08–0.2)
High-middle SDI	435,935.66 (300,652.64–602,950.05)	60.34 (46.52–77.1)	827,741.17 (571,861.8–1,134,881.72)	57.97 (44.57–74.2)	0.9% (0.84–0.96)	−0.12 (−0.15 to −0.09)
High SDI	541,597 (376,964.16–733,826.69)	71.45 (55.34–90.2)	939,060.48 (696,820.22–1,228,719.31)	69.22 (55.37–85.18)	0.73% (0.63–0.87)	0.26 (0.09–0.44)
**Regions**						
Andean-Latin America	1,894.12 (1,297.77–2,554.18)	13.63 (10.08–17.53)	6,097.32 (4,265.51–8,160.3)	14.84 (11.11–19.17)	2.22% (2.07–2.38)	0.43 (0.35–0.52)
Australasia	17,400.97 (12,027.61–23,837.67)	98.68 (75.18–125.06)	32,335.39 (22,689.52–43,431.81)	90.39 (69.71–114.51)	0.86% (0.75–0.97)	−0.24 (−0.3 to −0.19)
Caribbean	5,691.5 (3,900.06–7,753.2)	29.33 (22.05–38.08)	11,441.62 (7,849.53–15,488.95)	29.72 (22.18–38.44)	1.01% (0.96–1.07)	0.07 (0.06–0.08)
Central Asia	18,123.03 (12,503.21–24,707.24)	63.51 (48.9–80.87)	27,908.61 (18,911.11–38,566.56)	64.77 (49.64–82.66)	0.54% (0.47–0.6)	0.1 (0.09–0.12)
Central Europe	69,623.44 (47,658.46–96,047.25)	72.77 (55.81–92.66)	98,521.6 (67,133.05–136,390.75)	70.59 (54.18–89.82)	0.42% (0.36–0.46)	0.02 (−0.06–0.11)
Central Latin America	17,879.46 (12,333.8–24,307.64)	31.81 (23.94–41.05)	53,721.27 (37,296.58–73,064.04)	31.77 (23.84–41.21)	2% (1.94–2.07)	0.01 (0–0.02)
Central Sub-Saharan Africa	4,453.46 (2,990–6,276.85)	33.03 (25.06–42.21)	9,596.86 (6,537.13–13,243.4)	32.22 (24.45–41.46)	1.15% (1.01–1.31)	−0.1 (−0.12 to −0.09)
East Asia	313,996.06 (212,426.67–436,983.35)	55.26 (41.95–70.12)	845,659.53 (583,221.77–1,164,608.61)	57.4 (43.67–72.99)	1.69% (1.61–1.8)	0.16 (0.04–0.28)
Eastern Europe	122,722.76 (84,488.3–167,427.12)	68.4 (52.4–87.04)	167,038.62 (114,134.66–231,211.1)	74.4 (57.18–95.28)	0.36% (0.32–0.4)	0.35 (0.32–0.38)
Eastern Sub-Saharan Africa	8,610.45 (5,833.87–11,972.16)	19.48 (14.83–25.05)	18,593.54 (12,619.36–25,545)	19.89 (15.18–25.61)	1.16% (1.11–1.22)	0.16 (0.1–0.23)
High-income Asia Pacific	31,243.1 (20,944.28–43,603.15)	26.92 (20.67–34.42)	49,432.85 (33,174.99–68,914.26)	21.12 (16.28–27.19)	0.58% (0.5–0.7)	−1.58 (−1.91 to −1.24)
High-income North America	265,521.57 (184,073.28–358,286.57)	95.3 (73.03–121)	548,955.86 (418,372.27–698,785.69)	108.53 (87.59–131.44)	1.07% (0.86–1.34)	1.33 (0.95–1.72)
North Africa and the Middle East	45,957.21 (31,425.75–63,186.57)	41.81 (31.63–53.41)	116,006.36 (80,041.9–158,616.64)	41.93 (31.66–53.56)	1.52% (1.46–1.61)	−0.08 (−0.11 to −0.05)
Oceania	1,027.48 (699.01–1,447.68)	57.91 (43.98–73.48)	2,374.01 (1,625.76–3,294.83)	59.26 (44.94–75.2)	1.31% (1.19–1.43)	0.07 (0.05–0.09)
South Asia	207,889.94 (142,458.26–289,158.44)	60.06 (45.66–76.21)	586,125.53 (400,228.16–804,858.9)	61.37 (46.53–78)	1.82% (1.74–1.94)	0.06 (0.06–0.07)
Southeast Asia	97,679.58 (66,820.01–134,561.02)	61.29 (46.46–78.2)	247,606.5 (169,923.94–339,117.02)	62.77 (47.54–79.6)	1.53% (1.49–1.58)	0.09 (0.08–0.1)
Southern Latin America	13,914.25 (9,465.08–18,990.5)	40.73 (30.74–52.21)	25,772.38 (17,760.5–35,322.51)	41.8 (31.8–53.39)	0.85% (0.76–0.95)	0.07 (0–0.14)
Southern Sub-Saharan Africa	6,675.23 (4,564.91–9,166.84)	37.43 (28.33–48.01)	13,535.76 (9,269.77–18,486.87)	37.09 (28.17–47.62)	1.03% (0.98–1.08)	−0.02 (−0.03 to −0.01)
Tropical Latin America	26,492.79 (18,160.56–36,288.69)	43.44 (32.89–55.6)	77,585.15 (53,528.43–105,946.86)	44.8 (33.81–57.84)	1.93% (1.84–2.04)	0.55 (0.39–0.7)
Western Europe	286,609.98 (200,891.8–389,866.68)	72.79 (55.34–92.55)	352,998.6 (247,983.17–481,656.6)	63.01 (48.37–79.79)	0.23% (0.2–0.26)	−0.36 (−0.39 to −0.32)
Western Sub-Saharan Africa	19,617.2 (13,303.69–27,132.38)	35.51 (26.88–45.46)	39,836.76 (27,328.41–54,873.98)	36.01 (27.33–46.19)	1.03% (1–1.07)	0.05 (0.01–0.08)
**Characteristics**	**1990**		**2019**		**1990–2019**	**1990–2019**
	**Deaths cases No. (95% UI)**	**ASMR per 100,000 No. (95% UI)**	**Deaths cases No. (95% UI)**	**ASMR per 100,000 No. (95% UI)**	**Percentage change No. (95% CI)**	**EAPC No. (95% CI)**
**Overall**	92,801.05 (82,995.05–111,456.15)	4.29 (3.73–5.09)	219,437.14 (190,525.86–249,642.14)	4.38 (3.7–5.05)	1.36% (1.14–1.55)	0.04 (0.02–0.06)
**Sociodemographic index**						
Low SDI	3,728.16 (2,483.7–4,731.5)	3.73 (2.42–4.76)	10,484.14 (7,648.72–12,654.91)	4.3 (3.08–5.25)	1.81% (1.34–2.51)	0.5 (0.47–0.53)
Low-middle SDI	8,678.73 (6,914.63–10,558.98)	3.46 (2.66–4.14)	29,699.98 (25,034.79–34,583.16)	4.21 (3.5–4.87)	2.42% (1.76–3.14)	0.61 (0.55–0.67)
Middle SDI	16,620.38 (14,704.44–18,971.98)	3.82 (3.32–4.28)	52,178.17 (45,208.24–61,158.62)	4.11 (3.5–4.75)	2.14% (1.71–2.6)	0.21 (0.19–0.24)
High-middle SDI	27,689.09 (24,854.06–35,444.35)	4.64 (4.04–5.92)	58,332.62 (50,409.75–69,838.57)	4.47 (3.78–5.39)	1.11% (0.89–1.28)	−0.25 (−0.32 to −0.18)
High SDI	36,029.71 (31,280.88–45,846.61)	4.62 (3.93–5.82)	68,619.71 (56,411.87–81,382)	4.61 (3.67–5.52)	0.9% (0.65–1.04)	0.03 (0.01–0.06)
**Regions**						
Andean-Latin America	504.52 (424.82–588.91)	4.41 (3.67–5.06)	1,525.05 (1,231.71–1,852.46)	4.32 (3.48–5.24)	2.02% (1.46–2.77)	0.1 (0.03–0.18)
Australasia	1,158.23 (958.03–1,334.33)	7.38 (5.96–8.41)	2,511.5 (2,016.89–3,042.33)	6.86 (5.45–8.33)	1.17% (0.96–1.42)	−0.44 (−0.52 to −0.36)
Caribbean	697.31 (592.49–813.9)	4.46 (3.72–5.28)	1,549.42 (1,297.45–1,884.17)	4.73 (3.91–5.79)	1.22% (0.92–1.56)	0.26 (0.16–0.36)
Central Asia	933.47 (773.86–1,271.8)	3.62 (2.82–4.8)	1,951.57 (1,721.37–2,586.44)	5.75 (4.95–7.74)	1.09% (0.62–1.45)	1.49 (1.33–1.64)
Central Europe	4,332.08 (3,890.56–5,357.81)	4.51 (3.93–5.43)	8,073.21 (6,837.02–9,536.65)	4.83 (3.99–5.7)	0.86% (0.61–1.09)	0.15 (0.1–0.2)
Central Latin America	1,960.79 (1,699.83–2,517.32)	4.46 (3.76–5.68)	6,478.07 (5,267.49–8,265.53)	4.56 (3.72–5.83)	2.3% (1.87–2.71)	−0.09 (−0.17 to −0.01)
Central Sub-Saharan Africa	467.17 (268.6–797.97)	5 (2.85–8.21)	1,334.27 (857.82–1,933.42)	5.63 (3.55–8.24)	1.86% (1.15–2.84)	0.37 (0.29–0.45)
East Asia	14,205.46 (1,1974.33–16,636.49)	4 (3.31–4.63)	41,526.2 (35,287.76–47,920.9)	3.82 (3.19–4.42)	1.92% (1.36–2.59)	−0.26 (−0.31 to −0.21)
Eastern Europe	7,005.17 (6,098.93–9,688.8)	4.03 (3.44–5.6)	11,419.28 (9,643.68–14,750.74)	4.62 (3.88–6.02)	0.63% (0.43–0.83)	0.22 (0.07–0.36)
Eastern Sub-Saharan Africa	1,440.88 (840.03–1,891.54)	4.23 (2.37–5.58)	3,586.81 (2,304.99–4,437.21)	4.69 (2.98–5.87)	1.49% (0.99–2.18)	0.34 (0.25–0.43)
High-income Asia Pacific	3,746.97 (3,218.5–4,775.05)	3 (2.54–4.03)	9,103.35 (7,090.93–11,637.45)	2.39 (1.87–3.19)	1.43% (1.13–1.72)	−0.85 (−0.94 to −0.75)
High-income North America	11,153.7 (9,461.43–13,912.54)	4.04 (3.36–4.99)	22,725.4 (18,505.88–27,506.97)	4.96 (4.01–6.05)	1.04% (0.84–1.12)	0.72 (0.64–0.8)
North Africa and the Middle East	2,650.72 (2,061.78–3,059.75)	3.48 (2.67–4.11)	7,696.85 (6,582.67–9,421.38)	3.66 (3.07–4.33)	1.9% (1.45–2.87)	0.19 (0.1–0.28)
Oceania	44.44 (32.32–62.72)	4.1 (2.76–6.01)	121.79 (92.59–161.5)	4.25 (3.24–5.5)	1.74% (1.25–2.31)	0.17 (0.12–0.22)
South Asia	7,479.16 (5,630.22–9,687.24)	3.33 (2.41–4.27)	29,215.99 (22,995.29–36,408.38)	4.1 (3.18–5.15)	2.91% (1.92–4.04)	0.59 (0.47–0.71)
Southeast Asia	3,464.3 (3,046.27–3,994.08)	3.19 (2.72–3.64)	11,360.88 (9,546.76–13,785.34)	3.99 (3.27–4.75)	2.28% (1.7–2.91)	0.76 (0.7–0.81)
Southern Latin America	1,383.83 (1,183.52–1,701.62)	4.92 (4.13–5.93)	3,074.48 (2,606.14–4,013.77)	5.32 (4.44–6.94)	1.22% (0.99–1.46)	0.29 (0.2–0.38)
Southern Sub-Saharan Africa	409.26 (344.19–464.65)	3.1 (2.59–3.5)	1,045.72 (916.49–1,151.35)	3.94 (3.38–4.37)	1.56% (1.23–1.91)	0.79 (0.59–0.99)
Tropical Latin America	2,187.75 (1,873.43–2,735.8)	4.78 (3.99–5.95)	7,598.03 (6,107.51–8,898.99)	5.03 (4.01–5.94)	2.47% (1.93–2.73)	0.5 (0.32–0.69)
Western Europe	25,871.16 (22,624.18–34,903.21)	5.77 (4.91–7.83)	43,760.97 (35,707.5–52,790.04)	5.76 (4.59–6.99)	0.69% (0.38–0.86)	0.06 (0.02–0.09)
Western Sub-Saharan Africa	1,704.67 (1,332.45–2,229.05)	4.5 (3.55–5.93)	3,778.27 (3,177.8–4,408.07)	4.75 (3.89–5.59)	1.22% (0.57–1.76)	0.08 (0.04–0.12)
**Characteristics**	**1990**		**2019**		**1990–2019**	**1990–2019**
	**DALYs cases No. (95% UI)**	**ASDR per 100,000 No. (95% UI)**	**DALYs cases No. (95% UI)**	**ASDR per 100,000 No. (95% UI)**	**Percentage change No. (95% CI)**	**EAPC No. (95% CI)**
**Overall**	3,044,717.13 (2,363,647.01–3,900,467.06)	110 (87.66–139.16)	6,579,977.8 (5,241,696.12–8,321,653.38)	107.13 (86.18–133.73)	1.16% (1.06–1.25)	−0.03 (−0.06–0)
**Sociodemographic index**						
Low SDI	114,659.53 (85,965.49–146,544.67)	84.28 (63.03–105.84)	287,302.79 (224,193.31–354,685.81)	91.91 (71.89–111.57)	1.51% (1.27–1.81)	0.3 (0.27–0.33)
Low-middle SDI	326,477.32 (249,420.63–425,285.99)	91.5 (71.71–114.77)	927,307.72 (727,621.88–1,164,150)	101.01 (81.22–123.92)	1.84% (1.58–2.13)	0.34 (0.32–0.37)
Middle SDI	577,551.29 (444,346.58–745,572.44)	93.38 (74.4–116.56)	1,648,790.75 (1,285,587.8–2,095,429.98)	97.9 (78.07–122.72)	1.85% (1.67–2.05)	0.17 (0.14–0.2)
High-middle SDI	903,441.18 (702,229.35–1,176,141.28)	116.99 (92.57–149.81)	1,752,067.46 (1,361,490.97–2,252,102.91)	110.21 (86.73–140.14)	0.94% (0.84–1.02)	−0.25 (−0.28 to −0.23)
High SDI	1,121,109.75 (882,347.51–1,445,544.14)	128.62 (101.73–165.21)	1,961,481.71 (1,561,092.24–2,473,050.53)	122.64 (97.3–153.57)	0.75% (0.64–0.83)	0.03 (−0.05–0.11)
**Regions**						
Andean-Latin America	8,588.77 (7,326.22–10,022.07)	64.49 (55.4–74.42)	25,568.67 (21,197.9–30,491.13)	63.61 (52.67–75.22)	1.98% (1.5–2.57)	0.11 (0.04–0.18)
Australasia	35,222.44 (27,725.1–45,237.53)	183.72 (145.83–231.54)	70,166.25 (55,042.18–90,300.77)	168.28 (132.54–214.32)	0.99% (0.89–1.11)	−0.36 (−0.39 to −0.34)
Caribbean	14,971.71 (12,424.5–18,055.44)	79.4 (66.52–94.7)	31,795.63 (26,276.01–38,251.98)	83.27 (69.65–99.83)	1.12% (0.93–1.34)	0.19 (0.13–0.25)
Central Asia	35,540.25 (26,530.96–47,120.72)	109.82 (82.86–143.91)	61,772.11 (47,916.22–79,286.07)	138.35 (109.64–176.31)	0.74% (0.57–0.89)	0.82 (0.76–0.88)
Central Europe	155,815.55 (120,452.79–203,689.02)	134.74 (105.22–173.65)	251,596.5 (195,996.37–324,725.31)	136.04 (106.47–173.32)	0.61% (0.51–0.72)	0.07 (0.01–0.12)
Central Latin America	43,319.71 (36,219.34–53,516.31)	80.65 (68.44–98.18)	138,688.09 (113,679.15–171,245.23)	82.84 (68.32–101.95)	2.2% (1.93–2.47)	−0.01 (−0.05–0.04)
Central Sub-Saharan Africa	122,66.03 (8,247.28–18,438.06)	99.09 (65.94–148.49)	30,142.71 (21,400.77–39,321.26)	104.25 (72.95–138.1)	1.46% (0.98–1.99)	0.14 (0.07–0.2)
East Asia	519,048.51 (394,223.27–675,814.17)	98.28 (76.84–124.34)	1,432,517.88 (1,088,053.16–1,867,068.05)	96.78 (75.08–122.56)	1.76% (1.5–2.01)	−0.05 (−0.13–0.03)
Eastern Europe	258,196.1 (196,297.15–341,308.43)	120.85 (92.65–157.62)	384,777.08 (292,577.71–503,883.63)	136.59 (104.82–177.07)	0.49% (0.41–0.57)	0.36 (0.28–0.43)
Eastern Sub-Saharan Africa	31,126.86 (21,024.73–39,298.52)	73.06 (48.45–92.35)	71,336.96 (50,303.75–87,335.35)	77.09 (53.33–94.33)	1.29% (0.93–1.76)	0.18 (0.1–0.27)
High-income Asia Pacific	94,477.13 (77,636.52–118,368.25)	66.35 (54.42–82.7)	195,233.79 (157,805.14–243,046.5)	53.53 (43.1–66.58)	1.07% (0.92–1.23)	−1.06 (−1.22 to −0.91)
High-income North America	424,379.23 (327,241.59–558,162.82)	140.63 (108.35–183.61)	832,025.39 (649,706.07–1,063,351.64)	160.18 (125.7–202.67)	0.96% (0.83–1.09)	0.89 (0.71–1.07)
North Africa and the Middle East	81,921.61 (64,667.39–104,296.2)	79.91 (63.99–99.66)	217,791.49 (171,790.55–275,028.53)	81.61 (65.1–100.78)	1.66% (1.43–2.05)	0.01 (−0.01–0.04)
Oceania	1,714.91 (1,278.28–2,249.71)	109.88 (83.38–140.9)	4,265.72 (3,269.65–5,477.39)	116.58 (91.06–148.01)	1.49% (1.26–1.74)	0.23 (0.22–0.25)
South Asia	313,839.17 (233,856.25–415,086.12)	96.83 (73.96–124.55)	1,004,000.6 (767,638.51–1,284,863.5)	106.05 (82.89–132.86)	2.2% (1.83–2.62)	0.26 (0.22–0.31)
Southeast Asia	146,339.5 (109,111.94–193,284.06)	95.11 (73.3–122.94)	407,099.72 (309,292.37–534,122.36)	105.7 (82.22–133.52)	1.78% (1.59–2.01)	0.37 (0.34–0.4)
Southern Latin America	33,270.72 (27,276.91–41,107.45)	96.08 (79.7–117.41)	67,885.01 (55,648.18–84,703.48)	100.95 (83.35–125.77)	1.04% (0.92–1.17)	0.18 (0.13–0.22)
Southern Sub-Saharan Africa	12,289.22 (9,702.16–15,684.97)	71.59 (58.23–88.42)	27,739.42 (22,471.51–34,542.25)	79.24 (65.78–95.69)	1.26% (1.1–1.41)	0.36 (0.24–0.48)
Tropical Latin America	57,018.18 (46,603.14–71,324.15)	98.38 (81.69–120.63)	179,609.76 (147,086.35–221,530.81)	102.34 (84.28–124.34)	2.15% (1.86–2.3)	0.52 (0.4–0.64)
Western Europe	721,985.38 (571,968.84–930,654.64)	144.62 (114.54–185.07)	1,054,466.71 (845,195.76–1,323,431.16)	132.81 (105.93–168.11)	0.46% (0.32–0.56)	−0.19 (−0.22 to −0.16)
Western Sub-Saharan Africa	43,386.15 (33,451.62–54,984.95)	82.99 (65.49–103.45)	91,498.32 (73,641.02–111785.32)	86.19 (70–103.25)	1.11% (0.73–1.39)	0.08 (0.05–0.1)

ASIR, age-standardized incidence rate; ASMR, age-standardized mortality rate; ASDR, age-standardized DALYs rate; EAPC, estimated annual percentage change; CI, confidence interval; UI, uncertainty interval.

### Regional burden of AF

For SDI regions, the number of AF cases showed an upward trend in the five SDI countries from 2009 to 2019 (Table 1, Figure 1B). Interestingly, the higher the SDI region, the higher the morbidity and mortality. From 1990 to 2019, the number of incidences and DALY cases of AF rose most dramatically in the middle SDI. However, the number of deaths increased most substantially in low-middle SDI (Table 1, Figure 1B). Only high-middle SDI recorded a decreasing AF ASR, including ASIR (−0.12; 95% CI: −0.15 to −0.09), ASMR (−0.25; −0.32 to −0.18), and ASDR (−0.25; −0.28 to −0.23) of the EAPC (Table 1, Figure 1C, Supplementary Figure 1). Regarding the rest of the 21 GBD regions, the highest ASIR due to AF has occurred in high-income North America [108.53 (87.59–131.44)] since 2019 (Table 1, Supplementary Figure 2A). Similarly, the ASMR and ASDR of AF for men and women in Australasia were 6.86 (5.45–8.33) and 168.28 (132.54–214.32), respectively, which placed Australia in the top rank for AF related-mortality in 2019 (Table 1, Supplementary Figures 2B, C). It is also worth noting that the most prominent increases in the ASIR and ASDR were in high-income North America. Additionally, the most significant increases in the ASMR were exhibited in Central Asia, followed by southern sub-Saharan Africa, Southeast Asia, and high-income North America. During the research period, the most pronounced decrease in the ASIR, ASMR, and ASDR was reported, all in high-income Asia Pacific (Table 1, Supplementary Figure 3).

### National burden of AF

**Figure 2:**
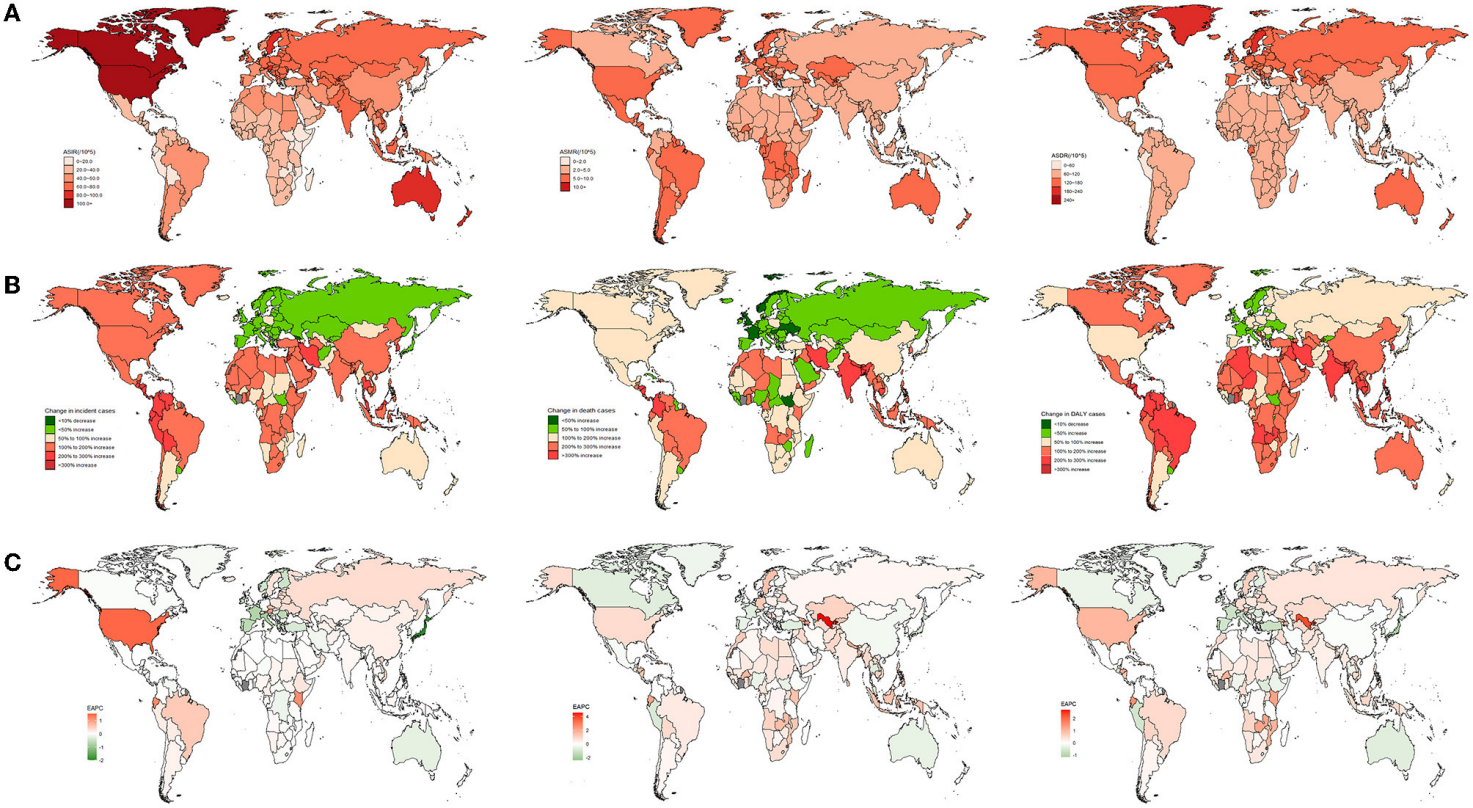
The national burden of AF for both sexes in 204 countries and territories. **(A)** The relative ASRs of AF between 1990 and 2019. **(B)** The relative changes of AF between 1990 and 2019. **(C)** The relative EAPCs of AF between 1990 and 2019.

In 2019, China presented the highest morbidity [818,493 (562,871–1,128,695)], mortality [39,970 (33,722–46,387)], and DALYs [1,383,674 (1,047,540–1,802,516)]. Coincidentally, all three accounted for approximately a quarter of the global caseload (Supplementary Tables 1–3). The United States of America exhibited the highest ASIR [109.52 (88.97–131.92)]; Montenegro showed the highest ASMR [14.38 (11.31–19.38)] and ASDR [250.62 (205.82–313.72)]. Conversely, Bolivia (Plurinational State of) had the lowest ASIR [14.41 (10.71–18.68)]; Singapore had the lowest ASMR [1.97 (1.44–2.37)] and ASDR [51.94 (40.06–67.04)] owing to AF (Supplementary Tables 1–3, Figure 2A). As for the percentage change, the most remarkable rise was in Qatar from 1990 to 2019, with a 7.91% increase in AF incidences; the most conspicuous rise was in Bahrain during the 30 years, with an 8.82% increase in deaths and a 6.51% increase in DALYs (Supplementary Tables 1–3, Figure 2B). Furthermore, the most apparent increase in ASIR was in the United States of America [1.51 (1.07–1.95)] from 1990 to 2019; the most significant rise in ASMR was in Uzbekistan [4.58 (4.23–4.94)]; the most demonstrable increases in the ASDR were in Bahrain [2.71(2.27–3.14)] and Uzbekistan [2.32 (2.17–2.47)] (Supplementary Tables 1–3, Figure 2C). By comparison, Japan displayed significant decreases in the ASIR and ASDR; Guam exhibited the most pronounced decline in the ASMR (Supplementary Tables 1–3, Figure 2C).

### The influential factors for EAPC

**Figure 3:**
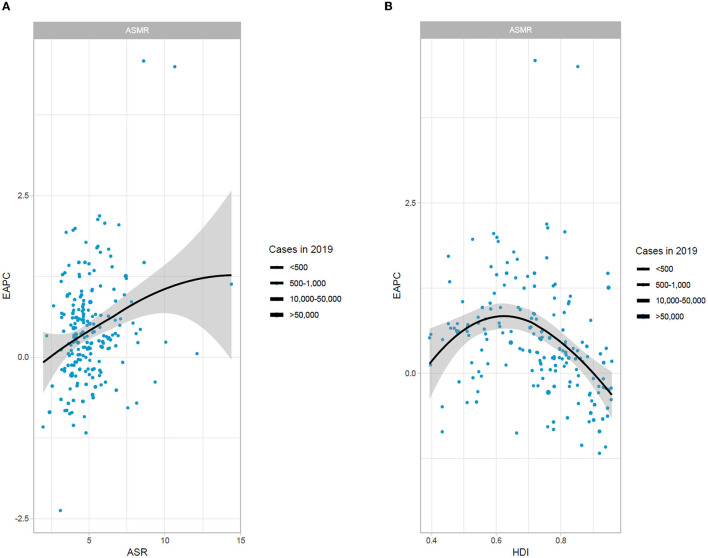
The association between EAPC and ASMR in 2019 and HDI in 2019. **(A)** The association between EAPC and ASMR in 2019. **(B)** The association between EAPC and HDI in 2019. EAPC, estimated annual percentage change; ASMR, age-standardized mortality rate; HDI, human development index.

Figure 3 shows that ASR (in 2019) and HDI (in 2019) seem to be particularly associated with EAPC. The ASMR of AF implies a healthy life expectancy from disease onset to eventual death. Meanwhile, HDI can be considered a metric to measure the accessibility and level of medical treatment. A significant positive relationship was observed between EAPCs and ASMR. Remarkably, a significant positive association was observed between EAPC and HDI when the HDI was restricted to <0.6. However, when the HDI was above 0.6, the countries with a higher HDI experienced a sharper decrease in EAPC of atrial fibrillation from 1990 to 2019 (Figure 3).

### Relationship of ASRs with SDI

**Figure 4:**
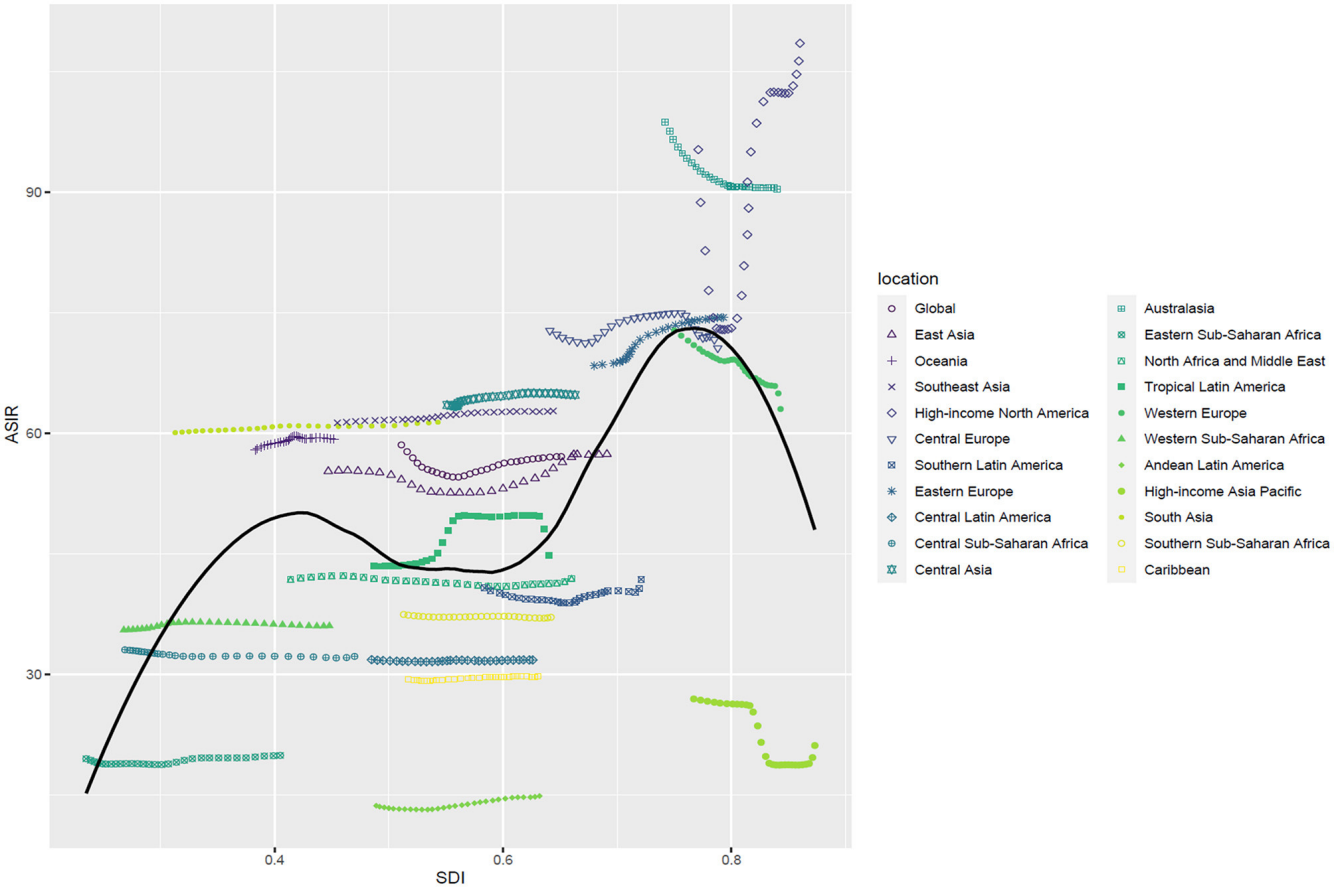
ASIR of AF in the global and 21 regions by SDI, 1990–2019. ASIR, age-standardized incidence rate; SDI, sociodemographic index.

A non-linear association was investigated between SDI and ASIR in global and 21 regions. The highest ASIR was found in high-income North America, with an SDI of 0.86. The ASIR was lowest in the Andean-Latin America area when the SDI was 0.53. At the region level, the ASIR values of 10 areas based on SDI in 2019 were higher than the global level, consisting of high-income North America, Australasia, Central Europe, Eastern Europe, Western Europe, Central Asia, Southeast Asia, South Asia, Oceania, and East Asia (Figure 4). The biggest ASMR and ASDR both appeared in Australasia, with SDI values of 0.77 and 0.74, respectively.

**Figure 5:**
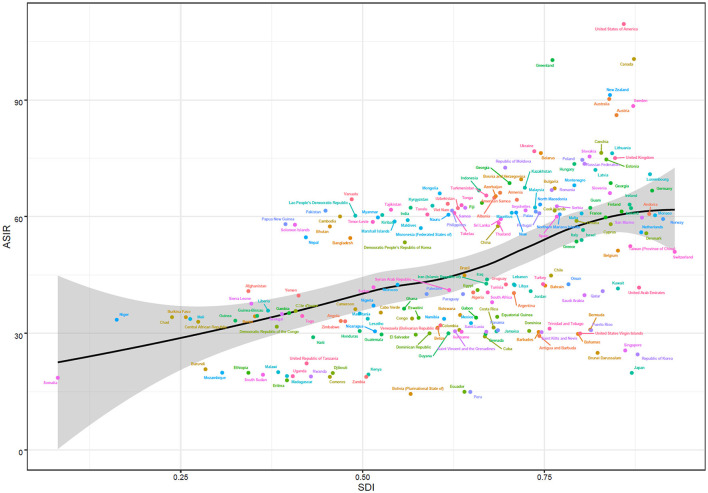
ASIR of AF in 204 countries by SDI, 1990–2019. ASIR, age-standardized incidence rate; SDI, sociodemographic index.

At the national level, we explored the relationship between ASIR and SDI values, which were complex and non-linear in 2019. Further analysis revealed that the ASIR in 204 countries and territories was positively correlated with the SDI. The largest ASIR was in the United States of America, followed by Canada and Greenland, according to the SDI. The ASIR was larger than the anticipated level in a number of territories. Nevertheless, Bolivia (the Plurinational State of), Peru, Ecuador, Eritrea, Somalia, and numerous other countries held a smaller ASIR than the expected SDI (Figure 5). Non-linear relationships among the SDI, ASMR, and ASDR of atrial fibrillation were also surveyed. The highest ASMR and ASDR were both noted in Montenegro when the SDI value was 0.79. However, the lowest ASMR and ASDR occurred in Singapore, with an SDI value of 0.86. And the majority of countries had lower ASMR and ASDR levels than expected, such as Japan, Kuwait, and the Republic of Korea. Intriguingly, burden estimates experienced an upward trend with increasing SDI, yet ASDR had a higher rise than ASMR (Supplementary Figures 4A, B).

### Risk factors for AF

**Figure 6:**
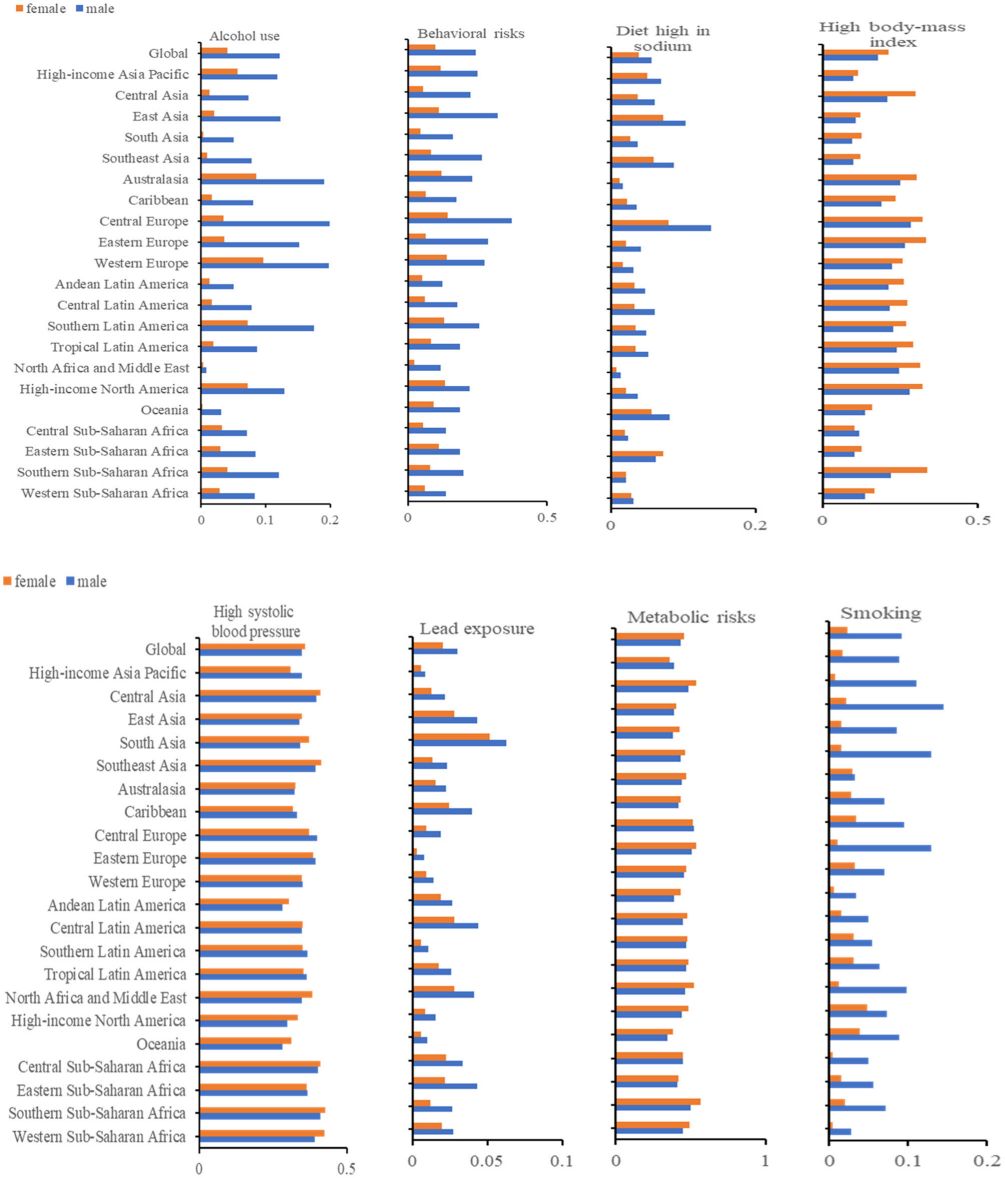
The age-standardized deaths are attributable to the risk factors.

Globally, 219,437 (190,526–249,642) individuals have died from AF. The mortality of AF in 2019 may be attributed to the referring risk factors evaluated by GBD: alcohol use, behavioral risks, a diet high in sodium, a high body mass index (BMI), high systolic blood pressure (SBP), lead exposure, metabolic risks, and smoking. Among them, the two main risk factors were a high BMI and a high SBP. The above risk factors displayed different percentages depending on the sexes at the regional level. For instance, the highest percentage of deaths due to smoking was detected in Central Europe, 52% for males. Simultaneously, the lowest percentage of deaths due to smoking was in High-income Asia Pacific, with 36% for females. In Central Europe, men (37%) and women (14%) had the highest mortality rates due to behavioral risks. Regarding high systolic blood pressure (SBP), the highest proportion of deaths occurred in southern sub-Saharan Africa (men, 41%; women, 43%) (Figure 6).

## Discussion

This is the first study to estimate the global burden and trends of AF in older adults aged 60–89 years and to recognize the associated risk factors. The results indicated that the burden of AF is gradually increasing globally, with an ~1.1-fold increase in incidences and an ~1.4-fold increase in deaths from 1990 to 2019. It may be attributable to improved survival with chronic diseases and the global population rising and aging (4, 13, 14). Moreover, the ASIR was higher in high-income North America, Australasia, Eastern Europe, and Central Europe; it was lower in Andean-Latin America, Eastern Sub-Saharan Africa, and high-income Asia Pacific, which was in accordance with the prevalence of AF (13). However, it was worth noting that the rate of increase in the prevalence of AF was extraordinarily rapid in high-income North America and surrounding areas. This observation may be due to the rapidly evolving regional economy, the Westernization tendency of personal diet, and the higher morbidity of cardiovascular problems and metabolic disorders (15).

Our findings also revealed that the degree of disease burden in AF differed between men and women. In general, the number of incident cases was higher in women, but the result of the age-standardized incidence rate was completely opposite, which was consistent with the perspectives of IHME (16). It may be related to our selection of older adults aged 60–89 years as our research subjects. An original research article demonstrated that the incidence rate of AF in women has increased significantly since the age of 60 (10). Longevity in women may be another reason for the increased morbidity of AF (10).

Furthermore, several studies have been conducted to show that estrogen plays a protective role against AF by prolonging the effective refractory period and reducing atrial pressure, which could profoundly interpret the increase in susceptibility to AF in post-menopausal women (17, 18). Andrade et al. also observed a higher prevalence of age-adjusted AF in men than in women (19), which may be partially explained by the lower lifetime risk of AF in women (20, 21). As for mortality, female patients with AF had a higher mortality rate, which may be due to the risk of thromboembolism (22). Previous studies have found female gender to be a major independent risk factor for thromboembolism in patients with AF (23, 24).

At the global level, the values of ASIR and ASDR were far higher than ASMR. Patients with AF were at higher risk for concurrent stroke, heart failure, hypertension, and other cardiovascular diseases than those without arrhythmias (25). In clinical practice, complications and co-morbidities have been documented as the leading cause of death in patients with AF (26). As a result, the actual number of deaths caused by AF may be hard to count, and the estimated disease burden of AF-related deaths is lower than the actual number of deaths. Our analysis also revealed characteristics in incidence, mortality rates, and DALYs in distinct SDI territories. In 2019, both morbidity and mortality were highest in high SDI regions, while the lowest morbidity and mortality occurred in low SDI and middle SDI regions, respectively. However, the most dramatic growth can be discerned in the low and low-middle SDI regions. From 1990 to 2019, ASIR, ASMR, and ASDR showed a decreasing trend in both high SDI and high-moderate SDI quintiles. In addition, the results reflected that the ASIR, ASMR, and ASDR were positively correlated with SDI, which signified that the higher the SDI region, the higher the burden of disease in older adults. These findings were roughly consistent with the research of other scholars (22, 27). Groups with higher access to health care, educational qualifications, and greater health awareness would focus more on their health (28–30). Therefore, AF was more likely to be discovered and reported in areas with a higher SDI. The effective and proven tactics used to manage the burden associated with AF in high SDI regions, such as the United States of America, may serve as a valuable source of policy inspiration for other regions with relatively low SDI quintiles.

Furthermore, we explored the association between ASMR, EAPC, and HDI. HDI is a meaningful indicator to evaluate the level of national social and economic development (31). There was an inverted U-shaped relationship diagram: mid-HDI regions had a higher EAPC of deaths than high-HDI regions. It implied that the ASMR of AF in high HDI areas experienced a decrease while low HDI areas experienced an increase. This may be attributed to the more powerful medical and health services, higher resource allocation, and residents' more aggressive attitude to prevent and treat AF and its complications (32). It was clear from the current study that the disease burden of AF is an urgent concern and an issue that needs to be addressed in less economically developed regions at present and in the future. Hence, it was imperative to establish health- and economic-based AF prevention initiatives for relevant national departments and regional health authorities.

Eventually, we explored the influencing risk factors of AF in older adults based on the GBD database, involving alcohol use, smoking, a diet high in sodium, a high BMI, a high SBP, lead exposure, and metabolic risks. The relationship between AF and the first six risk factors has already been identified (8, 33). Our analysis confirmed that high SBP and high BMI were principal considerations in the burden of AF. Credible evidence has demonstrated that high blood pressure (HBP) is an independent risk element for AF (34–36). A systematic review showed a 50% higher relative risk of AF in patients with HBP compared to those without HBP (37). Each 20 mm Hg increase in systolic blood pressure was associated with a 19% corresponding increase in the risk of atrial fibrillation (38). A few clinical mechanisms might account for the elevated risk of AF in people with HBP. Higher blood pressure leads to an increased risk of coronary artery disease and myocardial infarction (39), which can induce AF (4, 40, 41). Predictable epidemiological findings have demonstrated that increased blood pressure triggers inflammation in myocardial cells, contributing to fibrillation and an enlarged left ventricle (42, 43), which in turn raises the risk of suffering from AF (4, 44).

Furthermore, chronic hypertensive patients may experience impaired left ventricular hypertrophy and left ventricular contraction-diastolic function, which increase atrial pressure and eventually worsen atrial contraction (45). Experts believe controlling blood pressure within the normal range is conducive to reducing the occurrence of AF (46, 47). It is essential to adopt a low-salt diet and engage in home self-monitoring of blood pressure in daily life to prevent the occurrence of hypertension. High SBP and AF often co-exist, and the effect of obesity on AF should not be ignored. A former report highlighted that obesity elevated the risk of AF occurring by ~50% (48). Moreover, every 1 kg/m^2^ higher BMI was correlated with a 6% increase in risk (49), which was perceived as an independent predictor of incident AF (48, 50). Obesity exerted a proximate effect on cardiac muscle structure through enhanced exposure to oxidative strain (51). AF may be caused by electroanatomic remodeling in patients with a high BMI (52). Additionally, the role of BMI in generating the incidence of AF changed as age advanced. After the age of 60, alterations in physical components, such as increased fat mass and redistribution, may explain the increased influence of a high BMI on the risk of AF with age (53). As for the prognosis for patients with AF, a high BMI would increase the recurrence rate of AF (54) and the risk of paroxysmal atrial fibrillation developing into permanent atrial fibrillation (55). Therefore, weight control and physical exercise play a positive role in improving the prognosis and preventing the worsening of AF. In the future, both doctors and heart specialists are expected to incorporate recommendations on diet and lifestyle improvements, especially for obese older adults, into regular medical care. Moreover, metabolic syndrome may increase susceptibility to AF by activating significant oxidative stress signaling pathways and an inflammatory response (56). Metabolic syndrome is a kind of cardiovascular and metabolic disease that includes elevated blood pressure, overweight, hyperlipidemia, and insulin resistance (57). In the section above, we described the effects of high SBP and high BMI on AF, which contributed most to the increased risk of AF among the components of the metabolic syndrome (58). Considering the important impact of the metabolic syndrome on AF, a low-fat, low-sugar diet and regular monitoring of blood glucose and lipids are essential for formulating targeted AF prevention strategies in older adults. Additionally, we can create prevention and monitoring precautions according to the risk factors in different regions and by gender. For example, South Asia has higher mortality rates due to lead exposure than other regions, so avoiding lead exposure and reducing environmental lead concentrations could be used for AF prevention in South Asia. It was worth mentioning that the attribution proportions for smoking and alcohol consumption were significantly higher for men than for women. For men, quitting smoking and limiting alcohol to prevent the occurrence of AF should be valued.

Based on the findings above, we can conclude that our research is valuable in developing a worldwide strategy for effectively for screening AF in certain groups. More precisely, screening programs targeting risk factors are more effective. First, we should pay special attention to the older female population. Regular electrocardiogram examinations are necessary for them. Second, high SBP and high BMI have the greatest impact on AF. Thus, blood pressure and weight measurements must be included in the screening programs. Finally, we can establish screening plans for other risk factors. Smoking, alcohol consumption, a high-salt diet, and chronic lead exposure are key populations for AF screening, especially in males. In other words, high-risk individuals with AF need to undergo regular medical check-ups in specialized medical institutions with the goal of early screening, timely treatment planning, and delaying the progression of AF.

The current study has several limitations. First, our analysis was impacted by deficiencies in the 2019 GBD study methodology, which have been documented in previous literature (59–61). Insufficient and untimely registration of AF in some underdeveloped regions resulted in the underestimation of patients as well as misdiagnosis or under diagnosis owing to the lack of adequate medical resources. Overall, the data from some countries were not integral, which affected the precision and reliability of the results. Second, the GBD research did not meticulously classify AF into first-diagnosed AF, paroxysmal AF, persistent AF, and so on, based on clinical characteristics. Third, other pathological factors, such as rheumatic heart disease, hyperthyroidism, and pre-excitation syndrome, were not considered. Finally, the outbreak and prevalence of COVID-19 have had a serious impact on the burden of AF (62), and more evidence is urgently needed to compare changes in AF during the epidemic.

## Conclusion

In conclusion, AF in older adults is a major public health concern worldwide. The burden of AF varies widely at both national and regional levels. From 1990 to 2019, the cases of AF incidences, deaths, and DALYs have shown a global increase. The ASIR, ASMR, and ASDR have declined in the high-moderate and high SDI regions. However, the burden of AF increased promptly in the lower SDI regions. Moreover, it is necessary to consider the predominant risk factors for AF, such as high BMI and SBP, to encourage individuals with a high risk of AF to perform health management and early screening. By interpreting the burden of AF at different regional and national levels, our research illustrates the features of the global AF burden in pursuit of more effective and targeted prevention and treatment strategies.

## Data availability statement

The original contributions presented in the study are included in the article/Supplementary material, further inquiries can be directed to the corresponding authors.

## Author contributions

MJ, CL, and QG conducted a statistical analysis of the data. MJ drafted the manuscript. AM and QG conceptualized and designed the research and revised the manuscript. YL and YW collected and sorted the data. All authors contributed to the article and approved the submitted version.

## Supplementary material

Supplementary Figure 1 The EAPCs of AF ASRs from 1990 to 2019, both sexes by SDI. SDI, sociodemographic index.

Supplementary Figure 2 The regional burden of AF for both sexes in 2019. **(A)** ASIR in regions, 1990–2019; **(B)** ASMR in regions, 1990–2019; **(C)** ASDR in regions, 1990–2019. ASIR, age-standardized incidence rate; ASMR, age-standardized mortality rate; ASDR, age-standardized DALYs rate.

Supplementary Figure 3 The EAPCs of AF at the regional level, from 1990 to 2019.

Supplementary Figure 4 ASRs of AF in 204 countries by SDI, 1990–2019. **(A)** ASMR in 204 countries and territories, 2019; **(B)** ASDR in 204 countries and territories, 2019. ASMR, age-standardized mortality rate; ASDR, age-standardized DALYs rate; SDI, sociodemographic index.

Supplementary Table 1 The incidence of atrial fibrillation between 1990 and 2019 at national level.

Supplementary Table 2 The deaths of atrial fibrillation between 1990 and 2019 at national level.

Supplementary Table 3 The disability-adjusted life years (DALYs) of atrial fibrillation between 1990 and 2019 at national level.

## References

[1] Fang MC Chen J Rich MW. Atrial fibrillation in the older adults. Am J Med. (2007) 120:481–7. 10.1016/j.amjmed.2007.01.026 17524745

[2] Roth GA Johnson C Abajobir A Abd-Allah F Abera SF Abyu G. Global, regional, and national burden of cardiovascular diseases for 10 causes, 1990 to 2015. J Am Coll Cardiol. (2017) 70:1–25. 10.1016/j.jacc.2017.04.052 28527533 PMC5491406

[3] Guez D Boroumand G Ruggiero NJ Mehrotra P Halpern EJ. Automated and manual measurements of the aortic annulus with ECG-gated cardiac CT angiography prior to transcatheter aortic valve replacement: Comparison with 3D-transesophageal echocardiography. Acad Radiol. (2017) 24:587–93. 10.1016/j.acra.2016.12.008 28130049

[4] Schnabel RB Yin X Gona P Larson MG Beiser AS McManus DD. 50 year trends in atrial fibrillation prevalence, incidence, risk factors, and mortality in the Framingham Heart Study: A cohort study. Lancet. (2015) 386:154–62. 10.1016/S0140-6736(14)61774-8 25960110 PMC4553037

[5] Lippi G Sanchis-Gomar F Cervellin G. Global epidemiology of atrial fibrillation: An increasing epidemic and public health challenge. Int J Stroke. (2021) 16:217–21. 10.1177/1747493019897870 31955707

[6] Murray CJ Lopez AD. Measuring the globalburden of disease. N Engl J Med. (2013) 369:448–57. 10.1056/NEJMra1201534 23902484

[7] Wang L Ze F Li J Mi L Han B Niu H. Trends of global burden of atrial fibrillation/flutter from Global Burden of Disease Study 2017. Heart. (2021) 107:881–7. 10.1136/heartjnl-2020-317656 33148545

[8] Li H Song X Liang Y Bai X Liu-Huo WS Tang C. Global, regional, and national burden of disease study of atrial fibrillation/flutter, 1990-2019: Results from a global burden of disease study, 2019. BMC Public Health. (2022) 22:2015. 10.1186/s12889-022-14403-2 36329400 PMC9632152

[9] Osorio J Mansour M Melby D Imhoff RJ Hunter TD Maccioni S. Economic evaluation of contact force catheter ablation for persistent atrial fibrillation in the United States. Heart Rhythm O_2_. (2022) 3:647–55. 10.1016/j.hroo.2022.09.011 36589917 PMC9795304

[10] Magnussen C Niiranen TJ Ojeda FM Gianfagna F Blankenberg S Njølstad I. Sex differences and similarities in atrial fibrillation epidemiology, risk factors, and mortality in community cohorts: Results from the BiomarCaRE Consortium (Biomarker for Cardiovascular Risk Assessment in Europe). Circulation. (2017) 136:1588–97. 10.1161/CIRCULATIONAHA.117.028981 29038167 PMC5657474

[11] Global Burden of Disease Collaborative Network. Global Burden ofDisease Study 2016 (GBD 2016) Results. Seattle, United States: Institutefor Health Metrics and Evaluation (IHME) (2017). Available from http://ghdx.healthdata.org/gbd-results-tool.

[12] Liu Z Jiang Y Yuan H Fang Q Cai N Suo C. The trends in incidence of primary liver cancer caused by specific etiologies: Results from the Global Burden of Disease Study 2016 and implications for liver cancer prevention. J Hepatol. (2019) 70:674–83. 10.1016/j.jhep.2018.12.001 30543829

[13] Chugh SS Havmoeller R Narayanan K Singh D Rienstra M Benjamin EJ. Worldwide epidemiology of atrial fibrillation: A Global Burden of Disease 2010 Study. Circulation. (2014) 129:837–47. 10.1161/CIRCULATIONAHA.113.005119 24345399 PMC4151302

[14] Fordyce CB Roe MT Ahmad T Libby P Borer JS Hiatt WR. Cardiovascular drug development: Is it dead or just hibernating? J Am Coll Cardiol. (2015) 65:1567–82. 10.1016/j.jacc.2015.03.016 25881939

[15] Bhagavathula AS Shehab A Ullah A Rahmani J. The burden of cardiovascular disease risk factors in the Middle East: A systematic review and Meta-analysis focusing on primary prevention. Curr Vasc Pharmacol. (2021) 19:379–89. 10.2174/1573406416666200611104143 32525775

[16] Institute for Health Metrics Evaluation. GBD Compare Data Visualization. Seattle, WA: IHME, University of Washington (2018). Available online at: http://vizhub.healthdata.org/gbd-compare (accessed June 3, 2020).

[17] Tse HF Oral H Pelosi F Knight BP Strickberger SA Morady F. Effect of gender on atrial electrophysiologic changes induced by rapid atrial pacing and elevation of atrial pressure. J Cardiovasc Electrophysiol. (2001) 12:986–9. 10.1046/j.1540-8167.2001.00986.x 11573707

[18] Westerman S Wenger N. Gender differences in atrial fibrillation: A review of epidemiology, management, and outcomes. Curr Cardiol Rev. (2019) 15:136–44. 10.2174/1573403X15666181205110624 30516110 PMC6520576

[19] Andrade JG Deyell MW Lee AYK Macle L. Sex differences in atrial fibrillation. Can J Cardiol. (2018) 34:429–36. 10.1016/j.cjca.2017.11.022 29455950

[20] Ko D Rahman F Schnabel RB Yin X Benjamin EJ Christophersen IE. Atrial fibrillation in women: Epidemiology, pathophysiology, presentation, and prognosis. Nat Rev Cardiol. (2016) 13:321–32. 10.1038/nrcardio.2016.45 27053455 PMC5579870

[21] Alonso A Agarwal SK Soliman EZ Ambrose M Chamberlain AM Prineas RJ. Incidence of atrial fibrillation in whites and African-Americans: The Atherosclerosis Risk in Communities (ARIC) study. Am Heart J. (2009) 158:111–7. 10.1016/j.ahj.2009.05.010 19540400 PMC2720573

[22] Zhang J Johnsen SP Guo Y Lip GYH. Epidemiology of atrial fibrillation: Geographic/ecological risk factors, age, sex, genetics. Card Electrophysiol Clin. (2021) 13:1–23. 10.1016/j.ccep.2020.10.010 33516388

[23] Fang MC Singer DE Chang Y Hylek EM Henault LE Jensvold NG. Gender differences in the risk of ischemic stroke and peripheral embolism in atrial fibrillation: the AnTicoagulation and risk factors in atrial fibrillation (ATRIA) study. Circulation. (2005) 112:1687–91. 10.1161/CIRCULATIONAHA.105.553438 16157766 PMC3522521

[24] Lip GYH Nieuwlaat R Pisters R Lane DA Crijns HJGM. Refining clinical risk stratification for predicting stroke and thromboembolism in atrial fibrillation using a novel risk factor-based approach: The euro heart survey on atrial fibrillation. Chest. (2010) 137:263–72. 10.1378/chest.09-1584 19762550

[25] Virani SS Alonso A Benjamin EJ Bittencourt MS Callaway CW Carson AP. Heart disease and stroke statistics-2020 update: A report from the American Heart Association. Circulation. (2020) 141:e139–596. 10.1161/CIR.0000000000000757 31992061

[26] Deshpande S Catanzaro J Wann S. Atrial fibrillation: Prevalence and scope of the problem. Card Electrophysiol Clin. (2014) 6:1–4. 10.1016/j.ccep.2013.10.006 27063816

[27] Freedman B Hindricks G Banerjee A Baranchuk A Ching CK Du X. World heart federation roadmap on atrial fibrillation—A 2020 update. Glob Heart. (2021) 16:41. 10.5334/gh.1023 34211827 PMC8162289

[28] Zhang Y Su D Chen Y. Effect of socioeconomic status on the physical and mental health of the older adults: The mediating effect of social participation. BMC Public Health. (2022) 22:605. 10.1186/s12889-022-13062-7 35351078 PMC8962021

[29] Lövdén M Fratiglioni L Glymour MM Lindenberger U Tucker-Drob EM. Education and cognitive functioning across the life span. Psychol Sci Public Interest. (2020) 21:6–41. 10.1177/1529100620920576 32772803 PMC7425377

[30] Ding D Zhao Q Wu W Xiao Z Liang X Luo J. Prevalence and incidence of dementia in an older Chinese population over two decades: The role of education. Alzheimers Dement. (2020) 16:1650–62. 10.1002/alz.12159 32886438

[31] Veisani Y Jenabi E Khazaei S Nematollahi S. Global incidence and mortality rates in pancreatic cancer and the association with the Human Development Index: Decomposition approach. Public Health. (2018) 156:87–91. 10.1016/j.puhe.2017.12.015 29408193

[32] Chugh SS Roth GA Gillum RF Mensah GA. Global burden of atrial fibrillation in developed and developing nations. Glob Heart. (2014) 9:113–9. 10.1016/j.gheart.2014.01.004 25432121

[33] Xu S Chen Y Lin R Huang W Zhou H Lin Y. Burden of atrial fibrillation and its attributable risk factors from 1990 to 2019: An analysis of the Global Burden of Disease study 2019. Front Cardiovasc Med. (2022) 9:997698. 10.3389/fcvm.2022.997698 36386344 PMC9643162

[34] Wingerter R Steiger N Burrows A Estes NAM. Impact of lifestyle modification on atrial fibrillation. Am J Cardiol. (2020) 125:289–97. 10.1016/j.amjcard.2019.10.018 31761147

[35] Morin DP Bernard ML Madias C Rogers PA Thihalolipavan S. The state of the art: Atrial fibrillation epidemiology, prevention, and treatment. Mayo Clin Proc. (2016) 91:1778–810. 10.1016/j.mayocp.2016.08.022 27825618

[36] Manolis AJ Rosei EA Coca A Cifkova R Erdine SE Kjeldsen S. Hypertension and atrial fibrillation: Diagnostic approach, prevention and treatment. Position paper of the Working Group “Hypertension Arrhythmias and Thrombosis” of the European Society of Hypertension. J Hypertens. (2012) 30:239–52. 10.1097/HJH.0b013e32834f03bf 22186358

[37] Cameron A Cheng HK Lee R-P Doherty D Hall M Khashayar P. Biomarkers for atrial fibrillation detection after stroke: Systematic review and meta-analysis. Neurology. (2021) 97:e1775–89. 10.1212/WNL.0000000000012769 34504030

[38] Nazarzadeh M Pinho-Gomes A-C Bidel Z Canoy D Dehghan A Byrne KS. Genetic susceptibility, elevated blood pressure, and risk of atrial fibrillation: A Mendelian randomization study. Genome Med. (2021) 13:38. 10.1186/s13073-021-00849-3 33663581 PMC7934395

[39] Rapsomaniki E Timmis A George J Pujades-Rodriguez M Shah AD Denaxas S. Blood pressure and incidence of twelve cardiovascular diseases: Lifetime risks, healthy life-years lost, and age-specific associations in 1.25 million people. Lancet. (2014) 383:1899–911. 10.1016/S0140-6736(14)60685-1 24881994 PMC4042017

[40] Alonso A Krijthe BP Aspelund T Stepas KA Pencina MJ Moser CB. Simple risk model predicts incidence of atrial fibrillation in a racially and geographically diverse population: The CHARGE-AF consortium. J Am Heart Assoc. (2013) 2:e000102. 10.1161/JAHA.112.000102 23537808 PMC3647274

[41] Khurshid S Choi SH Weng L-C Wang EY Trinquart L Benjamin EJ. Frequency of cardiac rhythm abnormalities in a half million adults. Circ Arrhythm Electrophysiol. (2018) 11:e006273. 10.1161/CIRCEP.118.006273 29954742 PMC6051725

[42] Cao X Broughton ST Waits GS Nguyen T Li Y Soliman EZ. Interrelations between hypertension and electrocardiographic left ventricular hypertrophy and their associations with cardiovascular mortality. Am J Cardiol. (2019) 123:274–83. 10.1016/j.amjcard.2018.10.006 30390988

[43] Soliman EZ Byington RP Bigger JT Evans G Okin PM Goff DC. Effect of intensive blood pressure lowering on left ventricular hypertrophy in patients with diabetes mellitus: Action to control cardiovascular risk in diabetes blood pressure trial. Hypertension. (2015) 66:1123–9. 10.1161/HYPERTENSIONAHA.115.06236 26459421 PMC4644090

[44] Ogunmoroti O Michos ED Aronis KN Salami JA Blankstein R Virani SS. Life's Simple 7 and the risk of atrial fibrillation: The Multi-Ethnic Study of Atherosclerosis. Atherosclerosis. (2018) 275:174–81. 10.1016/j.atherosclerosis.2018.05.050 29920438

[45] Gumprecht J Domek M Lip GYH Shantsila A. Invited review: Hypertension and atrial fibrillation: epidemiology, pathophysiology, and implications for management. J Hum Hypertens. (2019) 33:824–36. 10.1038/s41371-019-0279-7 31690818

[46] Chen C Liu L Yu Y Shen G Huang J Huang Y. Association of systolic blood pressure with atrial fibrillation among treated hypertensive patients. Ann Palliat Med. (2020) 9:1752–63. 10.21037/apm-19-649 32527128

[47] Kim D Yang P-S Kim T-H Jang E Shin H Kim HY. Ideal blood pressure in patients with atrial fibrillation. J Am Coll Cardiol. (2018) 72:1233–45. 10.1016/j.jacc.2018.05.076 30190001

[48] Wanahita N Messerli FH Bangalore S Gami AS Somers VK Steinberg JS. Atrial fibrillation and obesity—Results of a meta-analysis. Am Heart J. (2008) 155:310–5. 10.1016/j.ahj.2007.10.004 18215602

[49] Zhao M Song L Zhao Q Chen Y Li B Xie Z. Elevated levels of body mass index and waist circumference, but not high variability, are associated with an increased risk of atrial fibrillation. BMC Med. (2022) 20:215. 10.1186/s12916-022-02413-1 35765047 PMC9241273

[50] Korantzopoulos P Kolettis TM. Obesity and the risk of new-onset atrial fibrillation. J Am Med Assoc. (2004) 292:2471–7. 10.1001/jama.292.20.2471 15562125

[51] Vincent HK Powers SK Stewart DJ Shanely RA Demirel H Naito H. Obesity is associated with increased myocardial oxidative stress. Int J Obes Relat Metab Disord. (1999) 23:67–74. 10.1038/sj.ijo.0800761 10094579

[52] Lavie CJ Pandey A Lau DH Alpert MA Sanders P. Obesity and atrial fibrillation prevalence, pathogenesis, and prognosis: Effects of weight loss and exercise. J Am Coll Cardiol. (2017) 70:2022–35. 10.1016/j.jacc.2017.09.002 29025560

[53] Senoo K Nakata M Teramukai S Yamamoto T Nishimura H Matoba S. Age-specific association between body mass index and the incidence of atrial fibrillation in Japanese men. Circ Rep. (2020) 2:466–70. 10.1253/circrep.CR-20-0067 33693271 PMC7819657

[54] Guglin M Maradia K Chen R Curtis AB. Relation of obesity to recurrence rate and burden of atrial fibrillation. Am J Cardiol. (2011) 107:579–82. 10.1016/j.amjcard.2010.10.018 21195377

[55] Tsang TSM Barnes ME Miyasaka Y Cha SS Bailey KR Verzosa GC. Obesity as a risk factor for the progression of paroxysmal to permanent atrial fibrillation: A longitudinal cohort study of 21 years. Eur Heart J. (2008) 29:2227–33. 10.1093/eurheartj/ehn324 18611964 PMC2733739

[56] Tadic M Ivanovic B Cuspidi C. What do we currently know about metabolic syndrome and atrial fibrillation? Clin Cardiol. (2013) 36:654–62. 10.1002/clc.22163 23788255 PMC6649365

[57] Yu S Guo X Li G Yang H Zheng L Sun Y. Gender discrepancies in predictors for newly onset cardiovascular events and metabolic syndrome in older adults patients from rural China. Front Cardiovasc Med. (2022) 9:995128. 10.3389/fcvm.2022.995128 36505366 PMC9726899

[58] Watanabe H Tanabe N Watanabe T Darbar D Roden DM Sasaki S. Metabolic syndrome and risk of development of atrial fibrillation: the Niigata preventive medicine study [published correction appears in Circulation. 2010 Aug 17;122:e433]. Circulation. (2008) 117:1255–60. 10.1161/CIRCULATIONAHA.107.744466 18285562 PMC2637133

[59] GBD 2016 Mortality Collaborators. Global, regional, and national under-5 mortality, adult mortality, age-specific mortality, and life expectancy, 1970–2016: A systematic analysis for the Global Burden of Disease Study 2016. Lancet. (2017) 390:1084–150. 10.1016/S0140-6736(17)31833-0 28919115 PMC5605514

[60] GBD 2016 Disease and Injury Incidence and Prevalence Collaborators. Global, regional, and national incidence, prevalence, and years lived with disability for 328 diseases and injuries for 195 countries, 1990–2016: A systematic analysis for the Global Burden of Disease Study 2016. Lancet. (2017) 390:1211–59. 10.1016/S0140-6736(17)32154-2 28919117 PMC5605509

[61] GBD 2016 Causes of Death Collaborators. Global, regional, and national age-sex specific mortality for 264 causes of death, 1980–2016: A systematic analysis for the Global Burden of Disease Study 2016. Lancet. (2017) 390:1151–210. 10.1016/S0140-6736(17)32152-9 28919116 PMC5605883

[62] Holt A Gislason GH Schou M Zareini B Biering-Sørensen T Phelps M. New-onset atrial fibrillation: Incidence, characteristics, and related events following a national COVID-19 lockdown of 56 million people. Eur Heart J. (2020) 41:3072–9. 10.1093/eurheartj/ehaa494 32578859 PMC7337750

