# Supplementary figures and images for: Estimates of the global, regional, and national burden of atrial fibrillation in older adults from 1990 to 2019: insights from the Global Burden of Disease study 2019
Source: Front Public Health. 2023 Jun 12;11:1137230. doi: 10.3389/fpubh.2023.1137230 (PMC10291625; doi:10.3389/fpubh.2023.1137230)

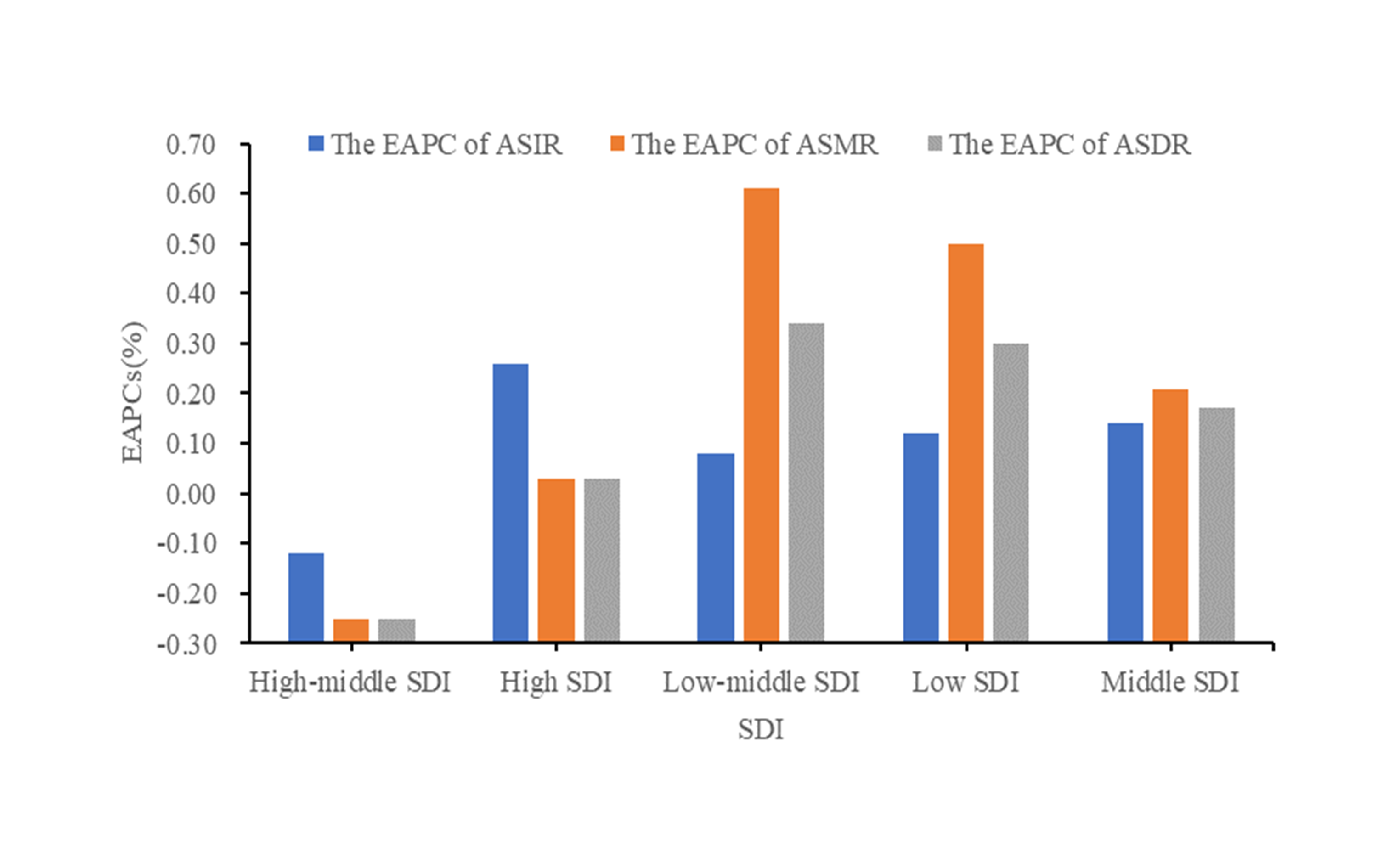

Supplement: Supplementary Figure 1 — The EAPCs of AF ASRs from 1990 to 2019, both sexes by SDI. SDI, sociodemographic index. [file Image_1.TIF]

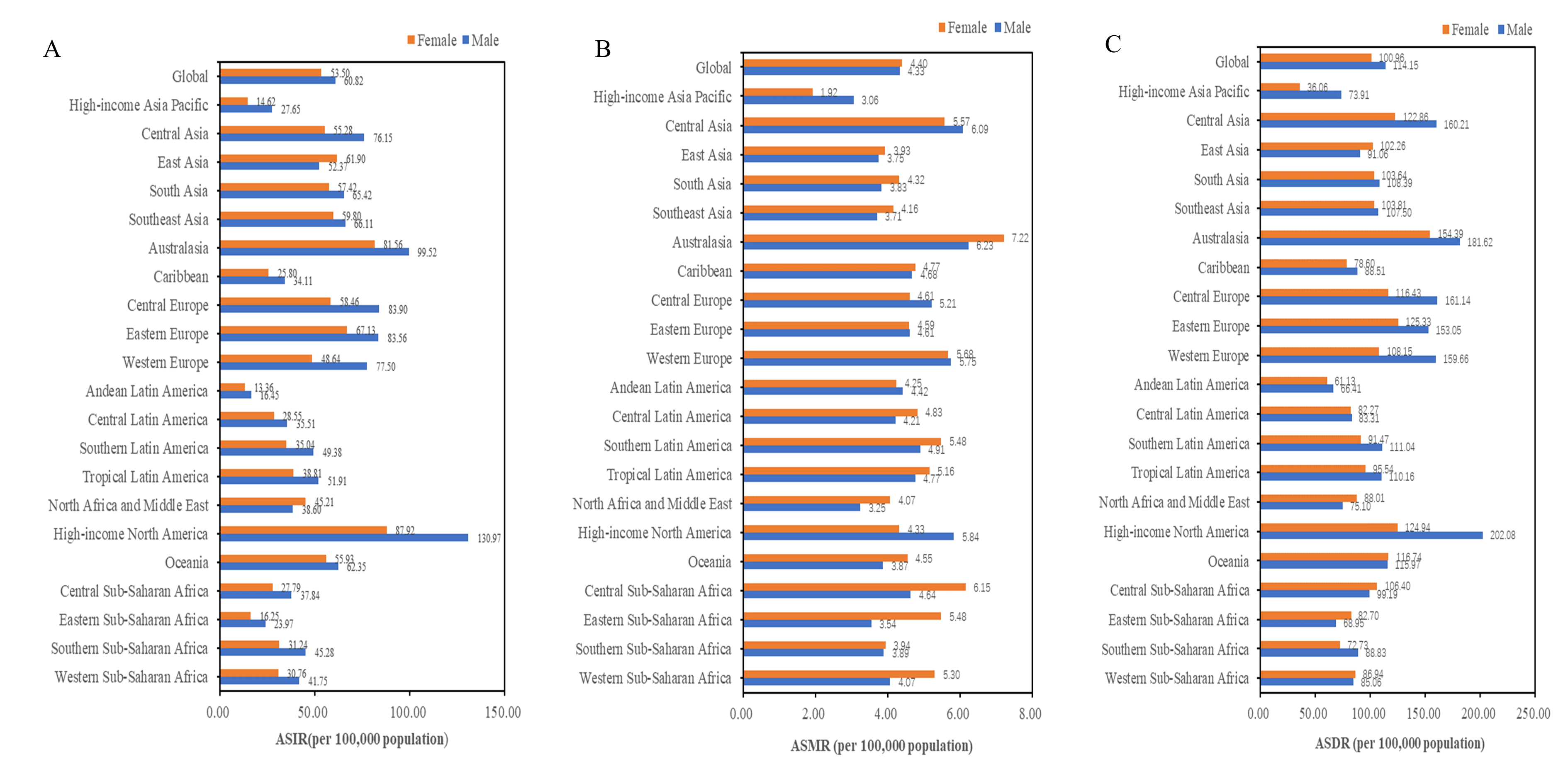

Supplement: Supplementary Figure 2 — The regional burden of AF for both sexes in 2019. (A) ASIR in regions, 1990–2019; (B) ASMR in regions, 1990–2019; (C) ASDR in regions, 1990–2019. ASIR, age-standardized incidence rate; ASMR, age-standardized mortality rate; ASDR, age-standardized DALYs rate. [file Image_2.TIF]

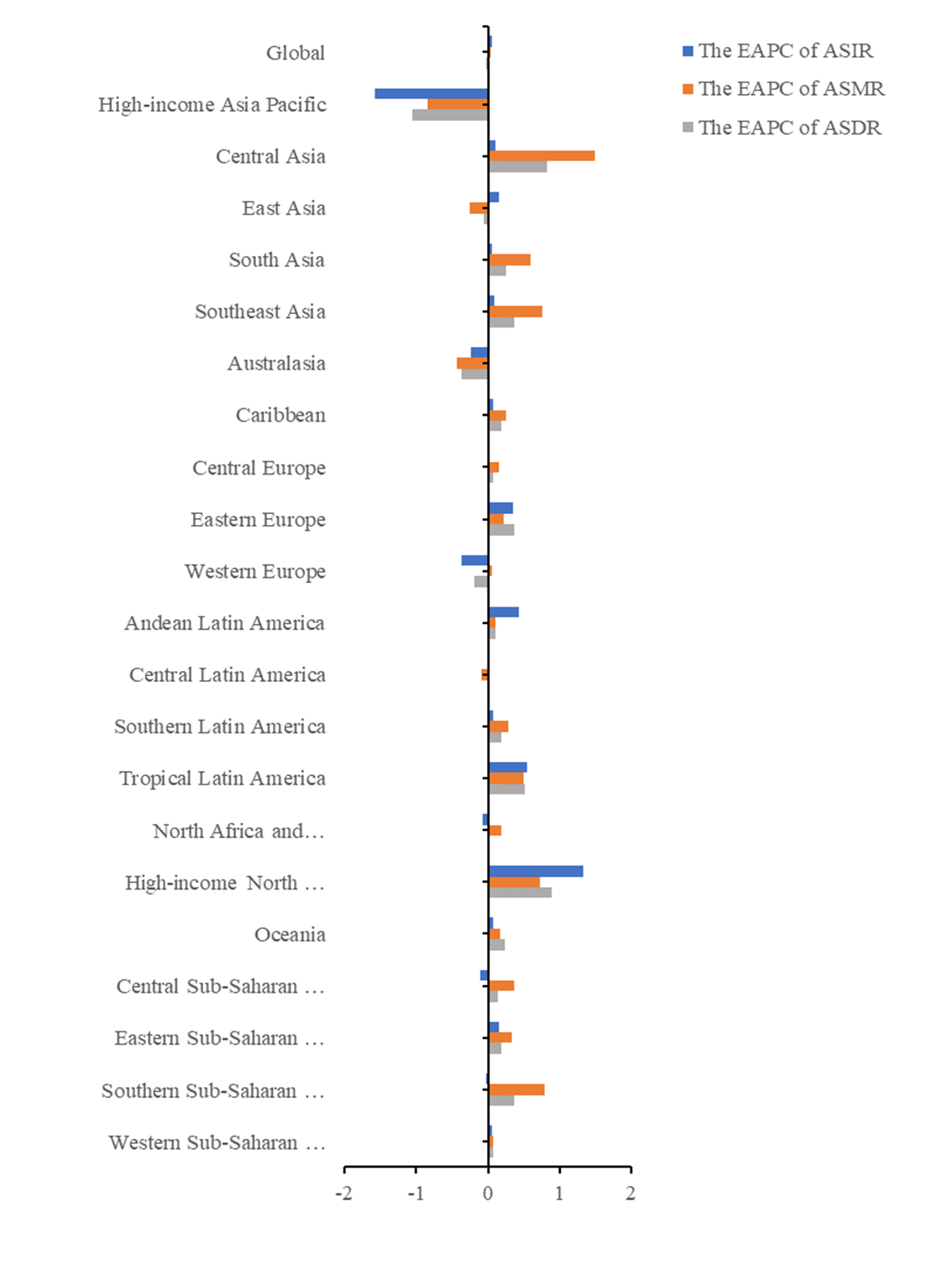

Supplement: Supplementary Figure 3 — The EAPCs of AF at the regional level, from 1990 to 2019. [file Image_3.TIF]

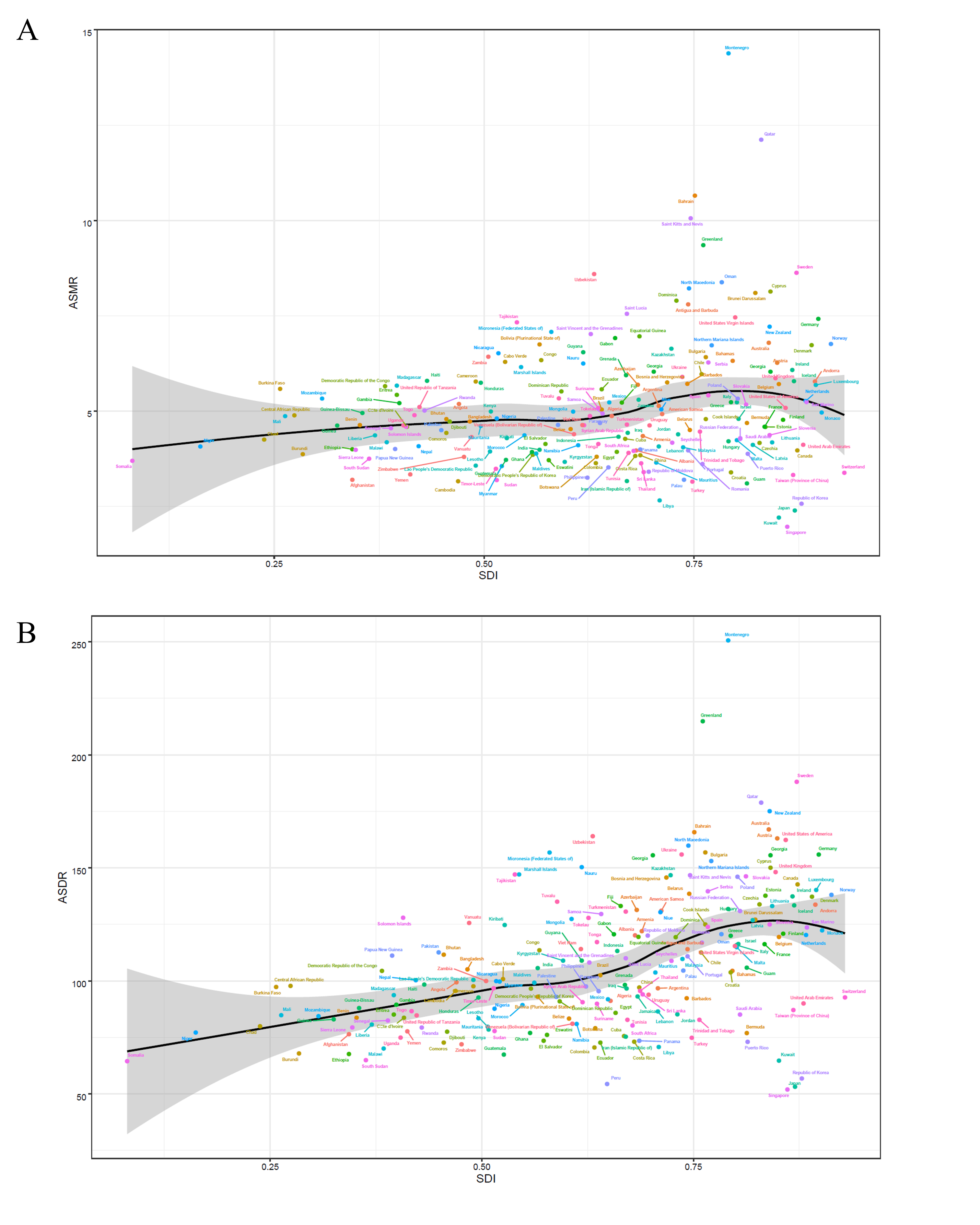

Supplement: Supplementary Figure 4 — ASRs of AF in 204 countries by SDI, 1990–2019. (A) ASMR in 204 countries and territories, 2019; (B) ASDR in 204 countries and territories, 2019. ASMR, age-standardized mortality rate; ASDR, age-standardized DALYs rate; SDI, sociodemographic index. [file Image_4.TIF]
