# Supplementary material for: Estimates of the global, regional, and national burden of atrial fibrillation in older adults from 1990 to 2019: insights from the Global Burden of Disease study 2019
Source: Front Public Health. 2023 Jun 12;11:1137230. doi: 10.3389/fpubh.2023.1137230 (PMC10291625; doi:10.3389/fpubh.2023.1137230)
Supplement: Supplementary Table 1 — The incidence of atrial fibrillation between 1990 and 2019 at national level. [file Table_1.DOCX]

Supplementary Table 1. The incidence of atrial fibrillation between 1990 and 2019 at national level.

| location | Case in 1990 | ASIR in 1990 | Case in 2019 | ASIR in 2019 | Percentage change | EAPC (95%) |
| --- | --- | --- | --- | --- | --- | --- |
| Afghanistan | 1907.76 (1306.49-2651.13) | 39.42 (29.98-50.72) | 2651.9 (1815.92-3683.32) | 40.85 (30.96-52.69) | 0.39% (0.28-0.5) | 0.15 (0.13-0.17) |
| Albania | 787.51 (534.49-1104.24) | 65.34 (49.91-83.91) | 1816.36 (1240.9-2497.34) | 64.84 (49.47-83.47) | 1.31% (1.11-1.46) | 0.04 (0.01-0.06) |
| Algeria | 3392.77 (2277.06-4694.94) | 40.13 (30.36-51.57) | 9251.87 (6394.17-12571.21) | 40.5 (30.55-52.6) | 1.73% (1.53-1.98) | 0 (-0.01-0.01) |
| American Samoa | 8.54 (5.78-11.91) | 62.18 (47.27-79.94) | 20.08 (13.7-27.69) | 64.33 (48.79-82.78) | 1.35% (1.2-1.52) | 0.08 (0.04-0.11) |
| Andorra | 26.94 (18.32-37.81) | 70.25 (53.5-90.02) | 52.73 (36.2-73.76) | 60.69 (45.67-77.75) | 0.96% (0.83-1.09) | -0.71 (-0.79--0.63) |
| Angola | 706.55 (476.09-991.26) | 32.87 (25.02-42.08) | 2032.7 (1382.82-2829.95) | 33.15 (25.02-42.47) | 1.88% (1.67-2.14) | 0.05 (0.03-0.06) |
| Antigua and Barbuda | 12.2 (8.26-16.62) | 28.34 (21.29-37.01) | 21.09 (14.46-28.7) | 29.31 (21.97-37.8) | 0.73% (0.54-0.91) | 0.14 (0.13-0.15) |
| Argentina | 9536.21 (6475.52-13006.92) | 39.57 (29.79-50.59) | 16212.84 (11122.71-22283.92) | 40.34 (30.69-51.65) | 0.7% (0.6-0.83) | 0.13 (0.1-0.16) |
| Armenia | 1076.63 (745.82-1443.36) | 64.69 (49.76-82.36) | 1861.52 (1288.15-2543) | 66.14 (50.45-84.61) | 0.73% (0.59-0.87) | 0.09 (0.08-0.1) |
| Australia | 14479.63 (9995.87-19781.75) | 98.3 (75-124.58) | 27165.47 (18992.55-36806.19) | 90.24 (68.96-115.19) | 0.88% (0.76-1.01) | -0.25 (-0.3--0.2) |
| Austria | 6195.91 (4762.77-7999.67) | 74.92 (61.35-90.61) | 9412.9 (6738.94-12806.99) | 86.1 (67.28-108.91) | 0.52% (0.33-0.72) | 1.03 (0.76-1.31) |
| Azerbaijan | 1828.12 (1265.77-2473) | 63.78 (48.76-81.18) | 3552.98 (2411.17-4847.69) | 65.25 (49.7-84.05) | 0.94% (0.81-1.08) | 0.15 (0.12-0.17) |
| Bahamas | 31.69 (21.34-43.28) | 30.32 (22.41-39.45) | 78.59 (54.25-106.11) | 29.84 (22.22-38.81) | 1.48% (1.29-1.66) | -0.02 (-0.03-0) |
| Bahrain | 41.9 (28.29-58.46) | 43.54 (32.94-56.27) | 216.57 (145.95-310.12) | 42.26 (31.85-54.76) | 4.17% (3.71-4.62) | -0.13 (-0.13--0.12) |
| Bangladesh | 16742.15 (11632.69-22969.21) | 55.78 (42.26-71.94) | 49679.2 (34126.74-67850.45) | 54.48 (41.11-69.99) | 1.97% (1.79-2.17) | -0.1 (-0.11--0.08) |
| Barbados | 73.28 (49.57-100.43) | 29.48 (21.98-38.35) | 118.18 (80.97-160.78) | 30.33 (22.71-39.56) | 0.61% (0.49-0.77) | 0.11 (0.1-0.11) |
| Belarus | 5609.25 (3844.78-7676.8) | 69.01 (52.94-88.38) | 7551.55 (5129.65-10350.39) | 76.34 (57.97-97.49) | 0.35% (0.26-0.44) | 0.39 (0.37-0.4) |
| Belgium | 6318.58 (4369.48-8654.3) | 59.38 (44.71-76.89) | 7236.23 (5123.79-9923.1) | 51.25 (39.9-65.15) | 0.15% (0.06-0.24) | -0.67 (-0.82--0.52) |
| Belize | 20.14 (13.64-27.52) | 29.89 (22.33-38.99) | 56.16 (38.42-76.91) | 31.41 (23.67-40.94) | 1.79% (1.61-1.98) | 0.21 (0.19-0.23) |
| Benin | 450.98 (307.19-622.32) | 34.01 (25.62-43.88) | 1004.57 (685.01-1383.46) | 34.37 (25.96-44.55) | 1.23% (1.1-1.36) | 0.04 (0.03-0.05) |
| Bermuda | 14.8 (10.1-20.28) | 31.81 (23.73-41.26) | 31.91 (21.89-43.24) | 30.93 (23.24-39.99) | 1.16% (1.01-1.3) | -0.14 (-0.17--0.11) |
| Bhutan | 85.78 (58.18-120.69) | 58.02 (43.86-74.26) | 219.93 (153.07-303.93) | 57.47 (43.71-73.71) | 1.56% (1.36-1.79) | -0.09 (-0.11--0.07) |
| Bolivia (Plurinational State of) | 291.17 (199.55-397.04) | 13.8 (10.21-17.83) | 894.77 (613.72-1219.86) | 14.41 (10.71-18.68) | 2.07% (1.89-2.28) | 0.2 (0.17-0.24) |
| Bosnia and Herzegovina | 1736.16 (1205.71-2366.96) | 72 (54.75-91.85) | 2729.34 (1858.94-3774.89) | 69.58 (53.44-88.8) | 0.57% (0.47-0.69) | -0.09 (-0.11--0.07) |
| Botswana | 123.87 (84.11-173.03) | 33.77 (25.61-43.6) | 283.68 (193.73-391.72) | 34.7 (26.26-44.71) | 1.29% (1.14-1.48) | 0.1 (0.09-0.11) |
| Brazil | 25880.51 (17740.81-35460.91) | 43.53 (32.97-55.73) | 76047.54 (52461.45-103870.41) | 44.91 (33.89-58) | 1.94% (1.84-2.06) | 0.56 (0.4-0.72) |
| Brunei Darussalam | 11.96 (7.85-17.07) | 27.42 (21.09-35.11) | 35.44 (23.35-51) | 24.96 (19.1-32.13) | 1.96% (1.72-2.21) | -0.33 (-0.37--0.29) |
| Bulgaria | 5998.81 (4066.61-8328.44) | 70.76 (53.82-90.07) | 6291.06 (4317.65-8807.62) | 67.26 (51.18-85.95) | 0.05% (-0.03-0.14) | -0.13 (-0.16--0.1) |
| Burkina Faso | 986.7 (659.55-1375.72) | 34.76 (26.19-44.64) | 1861.17 (1265.86-2552.33) | 34.13 (25.88-43.82) | 0.89% (0.76-1.02) | -0.09 (-0.11--0.07) |
| Burundi | 298.15 (201.55-415.41) | 20.22 (15.36-26.25) | 526.35 (353.77-741.56) | 20.74 (15.57-27.18) | 0.77% (0.6-0.9) | 0.06 (0.04-0.08) |
| Cabo Verde | 60.49 (40.66-82.75) | 34.96 (26.44-45.28) | 95.28 (66.17-127.23) | 35.33 (26.91-45.13) | 0.58% (0.46-0.71) | 0.02 (0.01-0.04) |
| Cambodia | 1574.59 (1064.92-2192.35) | 56.95 (42.77-73.05) | 4591.97 (3100.65-6376.38) | 60.03 (44.84-76.84) | 1.92% (1.73-2.14) | 0.17 (0.12-0.22) |
| Cameroon | 946.91 (639.7-1314.56) | 34.86 (26.15-45.12) | 2544.4 (1741.11-3507.33) | 36.19 (27.39-46.64) | 1.69% (1.51-1.85) | 0.2 (0.16-0.23) |
| Canada | 26244.81 (18468.97-35513.18) | 101.53 (77.57-129.83) | 54720.41 (38396.24-73947.63) | 100.53 (76.24-128.26) | 1.08% (0.98-1.21) | -0.05 (-0.06--0.04) |
| Central African Republic | 222.23 (149.03-311.55) | 33.58 (25.33-42.98) | 369.42 (248.43-516.13) | 32.95 (24.91-42.2) | 0.66% (0.56-0.77) | -0.08 (-0.09--0.07) |
| Chad | 633.88 (429.46-880.48) | 33.14 (25.11-42.42) | 1154.99 (794.46-1607.73) | 34.15 (25.95-43.76) | 0.82% (0.7-0.98) | 0.11 (0.1-0.11) |
| Chile | 3104.72 (2127.36-4292.04) | 43.99 (33.25-56.5) | 7859.14 (5418.7-10817.64) | 44.84 (33.73-57.62) | 1.53% (1.37-1.7) | -0.08 (-0.37-0.21) |
| China | 303020.75 (204936.69-421777.66) | 55.32 (42.01-70.18) | 818493.43 (562870.64-1128694.84) | 57.57 (43.76-73.12) | 1.7% (1.62-1.8) | 0.17 (0.04-0.29) |
| Colombia | 3624.32 (2483.28-4914.72) | 31.09 (23.46-40.28) | 12033.81 (8454.35-16285.52) | 30.85 (23.31-39.92) | 2.32% (2.09-2.57) | 0.01 (-0.01-0.04) |
| Comoros | 27.43 (18.67-37.63) | 18.94 (14.4-24.53) | 58.31 (39.81-80.61) | 18.79 (14.27-24.3) | 1.13% (1.01-1.24) | -0.03 (-0.04--0.03) |
| Congo | 223.26 (150.31-315.75) | 33.63 (25.39-43.46) | 495.67 (336.83-692.14) | 33.79 (25.47-43.79) | 1.22% (1.04-1.41) | 0.02 (0.01-0.03) |
| Cook Islands | 4.93 (3.38-6.79) | 59.2 (44.99-75.62) | 11.1 (7.57-15.16) | 61.55 (46.52-79.23) | 1.25% (1.1-1.45) | 0.1 (0.08-0.13) |
| Costa Rica | 390.93 (269.22-529.37) | 31.53 (23.61-40.68) | 1178.74 (814.64-1600.91) | 31.48 (23.68-40.64) | 2.02% (1.84-2.19) | 0 (-0.01-0) |
| Croatia | 2608.97 (1793.91-3553.03) | 63.85 (48.69-82.14) | 3226.7 (2346.35-4268.69) | 58.91 (46.39-73.03) | 0.24% (0.13-0.37) | -0.2 (-0.61-0.22) |
| Cuba | 2310.24 (1584.98-3140.75) | 29.16 (21.76-38.02) | 4175.75 (2852.58-5709.65) | 29.18 (21.79-37.55) | 0.81% (0.7-0.91) | 0.05 (0.03-0.06) |
| Cyprus | 382.2 (264.39-535.02) | 64.01 (48.77-81.73) | 788.44 (540.67-1111.24) | 58.04 (44.11-74.57) | 1.06% (0.92-1.2) | -0.7 (-0.92--0.48) |
| Czechia | 6811.09 (4665.21-9413.92) | 76.68 (58.31-98.09) | 10010.25 (6668.07-14347.67) | 76.39 (58.51-97.37) | 0.47% (0.34-0.58) | 0.67 (0.43-0.91) |
| C么te d'Ivoire | 788.04 (525.19-1114.68) | 35.83 (27.14-46.71) | 2126.05 (1430.71-2965.93) | 35.74 (27.08-46.23) | 1.7% (1.47-1.94) | -0.01 (-0.03-0) |
| Democratic People's Republic of Korea | 5036.35 (3404.06-6910.01) | 53.02 (39.85-67.69) | 11841.66 (8006.55-16109.54) | 53.38 (40.4-68.62) | 1.35% (1.13-1.67) | 0.01 (0-0.02) |
| Democratic Republic of the Congo | 3135.19 (2103.48-4470.64) | 32.97 (24.91-42.14) | 6386.77 (4365.96-8848.8) | 31.69 (24.09-40.86) | 1.04% (0.88-1.23) | -0.17 (-0.18--0.15) |
| Denmark | 3452.54 (2395.02-4746.75) | 63.07 (48.23-80.54) | 4228.93 (2921.96-5893.7) | 55.71 (42.39-71.53) | 0.22% (0.11-0.33) | -0.52 (-0.56--0.47) |
| Djibouti | 12.81 (8.57-17.86) | 18.61 (14.2-23.87) | 62.57 (41.78-88.01) | 19.78 (15.01-25.62) | 3.89% (3.61-4.26) | 0.22 (0.2-0.24) |
| Dominica | 17.88 (12.01-24.7) | 29.34 (21.86-38.27) | 21.24 (14.43-28.99) | 30.6 (22.83-39.8) | 0.19% (0.12-0.28) | 0.15 (0.14-0.16) |
| Dominican Republic | 749.94 (515.21-1021.66) | 29.41 (21.98-38.13) | 1978.37 (1375.86-2683.75) | 29.99 (22.52-38.96) | 1.64% (1.49-1.81) | 0.08 (0.06-0.09) |
| Ecuador | 452.45 (318.05-601.12) | 12.2 (9.09-15.53) | 1670.97 (1191.21-2191.23) | 14.95 (11.36-18.82) | 2.69% (2.37-3.01) | 1.18 (0.94-1.43) |
| Egypt | 7596.94 (5236.99-10398.67) | 40.87 (30.89-52.32) | 16488.63 (11198.61-22753.36) | 41.12 (30.92-52.81) | 1.17% (1.03-1.33) | 0.02 (0.01-0.03) |
| El Salvador | 602.75 (418.14-822.11) | 29.13 (21.81-37.7) | 1317.67 (909.95-1786.96) | 29.64 (22-38.29) | 1.19% (1.05-1.34) | 0.09 (0.06-0.11) |
| Equatorial Guinea | 39.07 (26.55-54.11) | 32.74 (24.72-41.96) | 96.07 (65.39-132.5) | 34.23 (25.9-44.28) | 1.46% (1.31-1.64) | 0.22 (0.19-0.25) |
| Eritrea | 81.44 (54.81-115.08) | 17.62 (13.37-22.84) | 242.36 (164.76-336.51) | 17.91 (13.6-23.12) | 1.98% (1.8-2.17) | 0.03 (0.02-0.03) |
| Estonia | 852.14 (587.38-1173.52) | 66.67 (50.31-85.43) | 1166.97 (806.39-1614.34) | 74.7 (56.79-95.71) | 0.37% (0.27-0.46) | 0.47 (0.43-0.51) |
| Eswatini | 60.09 (40.94-82.97) | 34.39 (26.01-44.11) | 123.08 (82.81-172.3) | 33.97 (25.65-43.73) | 1.05% (0.91-1.2) | -0.06 (-0.12-0.01) |
| Ethiopia | 2049.48 (1363.75-2880.57) | 18.41 (13.94-23.73) | 4878.16 (3362.97-6711.69) | 19.84 (15.06-25.55) | 1.38% (1.2-1.63) | 0.26 (0.24-0.28) |
| Fiji | 125.02 (85.5-173.94) | 61.01 (46.36-77.58) | 301.66 (204.31-423.14) | 63.54 (48.42-80.64) | 1.41% (1.3-1.54) | 0.13 (0.11-0.15) |
| Finland | 3384.24 (2355.05-4696.32) | 69.27 (52.65-88.02) | 4957.71 (3409.19-6946.16) | 61.32 (46.32-78.87) | 0.46% (0.36-0.59) | -0.48 (-0.53--0.43) |
| France | 40199.18 (27894.64-54914.68) | 72.9 (55.07-93.08) | 50874.62 (35280.65-71243.2) | 59.79 (44.98-76.73) | 0.27% (0.19-0.36) | -0.74 (-0.8--0.69) |
| Gabon | 127.15 (86.31-177.66) | 33.85 (25.62-43.99) | 216.23 (148.49-296.02) | 34.02 (25.9-43.85) | 0.7% (0.57-0.84) | 0.06 (0.04-0.07) |
| Gambia | 76.53 (51.64-105.72) | 35.4 (26.69-45.28) | 212.38 (144.52-288.87) | 35.2 (26.61-44.93) | 1.77% (1.59-2.01) | -0.02 (-0.04-0) |
| Georgia | 2730.25 (1872.85-3694.68) | 66.93 (51.3-84.52) | 2703.43 (1848.75-3676.39) | 68.62 (52.43-87.96) | -0.01% (-0.08-0.06) | 0.11 (0.09-0.13) |
| Germany | 66166.78 (46696.22-89817.96) | 80.22 (60.76-102.88) | 76630.64 (52779.8-105563.85) | 66.69 (50.48-85.63) | 0.16% (0.08-0.24) | -0.08 (-0.26-0.09) |
| Ghana | 1342.86 (910.62-1872.73) | 35.68 (27.03-45.87) | 3567.3 (2434.17-4936.8) | 36.33 (27.54-46.76) | 1.66% (1.52-1.81) | -0.01 (-0.06-0.05) |
| Greece | 6573.93 (4498.11-9086.38) | 62.37 (47.2-80.06) | 7704.48 (5324.27-10537.3) | 53.61 (40.5-68.25) | 0.17% (0.08-0.29) | -0.61 (-0.66--0.56) |
| Greenland | 20.71 (14.41-28.32) | 102.77 (78.64-131.61) | 48.45 (33.61-65.93) | 100.25 (76.51-126.96) | 1.34% (1.21-1.48) | -0.08 (-0.09--0.08) |
| Grenada | 17.23 (11.78-23.3) | 28.95 (21.77-37.36) | 23.43 (16.29-32.38) | 29.82 (22.55-39.12) | 0.36% (0.2-0.51) | 0.1 (0.09-0.1) |
| Guam | 27.24 (18.53-37.98) | 57.89 (44.02-73.81) | 81.71 (55.91-112.25) | 62.26 (47-79.85) | 2% (1.78-2.27) | 0.25 (0.22-0.27) |
| Guatemala | 691.54 (476.23-961.3) | 29.47 (22.01-38.29) | 2395.56 (1642.32-3221.28) | 29.7 (22.03-38.57) | 2.46% (2.15-2.82) | 0.1 (0.06-0.13) |
| Guinea | 740.78 (501.99-1028.85) | 32.77 (24.63-42.38) | 1165.15 (800.65-1608.89) | 33.25 (25.19-43.3) | 0.57% (0.47-0.69) | 0.06 (0.05-0.08) |
| Guinea-Bissau | 87.6 (59.03-123.97) | 34.64 (26.18-44.67) | 142.09 (96.43-196.69) | 34.48 (26.22-44.15) | 0.62% (0.52-0.74) | -0.01 (-0.01-0) |
| Guyana | 77.91 (52.97-106.26) | 30.75 (22.99-39.65) | 124.03 (85.65-171.47) | 29.99 (22.49-39.01) | 0.59% (0.46-0.71) | -0.1 (-0.1--0.09) |
| Haiti | 602.26 (408.99-829.64) | 28.86 (21.68-37.38) | 1278.06 (871.55-1748.91) | 28.98 (21.49-37.55) | 1.12% (0.98-1.3) | 0.02 (0.01-0.02) |
| Honduras | 415.55 (287.05-565.24) | 30 (22.47-38.64) | 1284.83 (874.39-1745.52) | 30.57 (22.74-39.42) | 2.09% (1.89-2.26) | 0.09 (0.06-0.11) |
| Hungary | 7668.03 (5191.76-10606.68) | 80.39 (61.4-103.23) | 9028.39 (6135.34-12662.41) | 73.54 (56.09-94.88) | 0.18% (0.09-0.26) | -0.33 (-0.36--0.3) |
| Iceland | 126.35 (87.53-173.66) | 67.06 (51.03-86.06) | 221.26 (151.34-309.89) | 62.17 (47.16-79.59) | 0.75% (0.64-0.87) | 0.27 (0.11-0.43) |
| India | 164638 (112257.15-230809.15) | 60.47 (46.01-76.77) | 486748.49 (332098.76-668462.81) | 62.29 (47.32-79.35) | 1.96% (1.84-2.12) | 0.09 (0.08-0.1) |
| Indonesia | 38210.11 (26026.21-53179.64) | 65.22 (49.53-83.02) | 89577.23 (61428.27-124144.27) | 66.74 (50.67-85.21) | 1.34% (1.28-1.41) | 0.07 (0.06-0.07) |
| Iran (Islamic Republic of) | 6833.62 (4613.86-9733.4) | 42.83 (32.58-54.95) | 20504.63 (14240.3-27858.08) | 42.76 (32.42-55.06) | 2% (1.73-2.4) | -0.14 (-0.28-0) |
| Iraq | 2280.4 (1567.08-3134.44) | 44.49 (33.18-57.43) | 6177.82 (4221.93-8447.56) | 43.84 (33.03-56.02) | 1.71% (1.53-1.92) | -0.03 (-0.06--0.01) |
| Ireland | 2003.17 (1382.1-2807.58) | 69.93 (53.51-89.57) | 3105.09 (2108.74-4359.71) | 63.1 (48-80.65) | 0.55% (0.42-0.66) | -0.53 (-0.61--0.44) |
| Israel | 2086.98 (1446.17-2896.94) | 61.23 (46.79-77.76) | 4384.76 (2987.08-6152.63) | 56.56 (42.84-72.52) | 1.1% (0.96-1.29) | -0.34 (-0.37--0.31) |
| Italy | 41687.5 (28934.22-57405.89) | 66.8 (51.14-85) | 46918.83 (32740.92-64208.89) | 53.97 (41.05-69.02) | 0.13% (0.08-0.18) | -0.84 (-0.92--0.76) |
| Jamaica | 404.49 (277.77-550.51) | 28.61 (21.59-37.04) | 660.84 (452.81-894.82) | 30.5 (22.7-39.62) | 0.63% (0.53-0.74) | 0.29 (0.26-0.32) |
| Japan | 26871.66 (17979.32-37369.19) | 26.69 (20.4-34.22) | 35710.85 (23697.58-49835.33) | 19.81 (15.36-25.34) | 0.33% (0.23-0.45) | -2.01 (-2.42--1.6) |
| Jordan | 315.44 (215.38-439.37) | 41.16 (30.9-53.02) | 1594.62 (1094.32-2199.16) | 40.83 (30.84-52.28) | 4.06% (3.78-4.39) | -0.03 (-0.04--0.02) |
| Kazakhstan | 5207.89 (3594.98-7113) | 67.01 (50.96-85.84) | 7122.46 (4824.1-9868.69) | 67.43 (51.31-86.52) | 0.37% (0.28-0.46) | 0.1 (0.07-0.13) |
| Kenya | 974.91 (664.92-1333.17) | 19.56 (14.86-25.12) | 2394.69 (1621.31-3314.8) | 19.39 (14.74-24.86) | 1.46% (1.39-1.52) | 1.17 (0.59-1.75) |
| Kiribati | 13.3 (8.93-18.64) | 58.99 (44.65-75.3) | 23.59 (15.73-33.4) | 60.42 (45.65-78.28) | 0.77% (0.65-0.9) | 0.08 (0.05-0.12) |
| Kuwait | 128.53 (89.25-177.21) | 41.36 (31.35-52.93) | 544.38 (377.58-738.73) | 41.51 (31.31-53.18) | 3.24% (2.97-3.64) | 0.04 (0.03-0.05) |
| Kyrgyzstan | 1171.35 (822.89-1586.92) | 61.8 (47.1-79.2) | 1624.66 (1108.87-2211.99) | 62.8 (48.12-79.41) | 0.39% (0.29-0.49) | 0.04 (0.01-0.06) |
| Lao People's Democratic Republic | 810.54 (553.05-1132.98) | 60.79 (46.13-77.63) | 1603.63 (1099.56-2218.2) | 60.23 (45.33-78.19) | 0.98% (0.85-1.14) | -0.04 (-0.05--0.03) |
| Latvia | 1464.76 (1015.58-1993.06) | 65.63 (50.1-83.9) | 1711.54 (1176.48-2359.04) | 72.02 (55.27-91.75) | 0.17% (0.08-0.24) | 0.47 (0.36-0.59) |
| Lebanon | 650.03 (449.22-899.79) | 41.93 (31.62-54.11) | 1650.67 (1123.81-2239.74) | 42.56 (31.75-54.97) | 1.54% (1.32-1.78) | 0.07 (0.04-0.1) |
| Lesotho | 215.7 (146.48-300.27) | 33.16 (25.17-42.82) | 270.77 (183.15-379.48) | 33.74 (25.51-43.27) | 0.26% (0.18-0.35) | 0.06 (0.03-0.08) |
| Liberia | 286.6 (191.73-402.44) | 36.89 (27.76-47.71) | 414.79 (283.03-574.4) | 35.82 (27.2-45.94) | 0.45% (0.34-0.55) | -0.1 (-0.13--0.08) |
| Libya | 499.67 (345.43-679.95) | 42.29 (31.85-54.71) | 1295.06 (882.03-1766.09) | 42.37 (31.72-54.56) | 1.59% (1.41-1.75) | 0 (-0.01-0.01) |
| Lithuania | 1912.29 (1319.2-2621.66) | 69.17 (52.82-88.19) | 2561.91 (1743.75-3512.93) | 76.29 (58.07-97.11) | 0.34% (0.25-0.45) | 0.43 (0.4-0.47) |
| Luxembourg | 271.31 (190.04-372.57) | 73.04 (56.14-93.3) | 436.38 (298.58-605.18) | 70.9 (53.21-90.73) | 0.61% (0.5-0.73) | 0.09 (-0.14-0.33) |
| Madagascar | 596.17 (403.14-824.15) | 18.94 (14.29-24.51) | 1079.04 (728.92-1494.94) | 19.01 (14.32-24.64) | 0.81% (0.68-0.94) | 0.02 (0.01-0.04) |
| Malawi | 457.88 (310.39-649.25) | 19.82 (15.13-25.76) | 870.65 (593.42-1210.97) | 20.01 (15.17-25.8) | 0.9% (0.77-1.04) | 0.02 (0.01-0.02) |
| Malaysia | 3565.55 (2454.29-4860.96) | 62.11 (47.08-79.4) | 11300.76 (7663.98-15627.72) | 62.34 (47.56-79.36) | 2.17% (1.97-2.38) | 0.05 (-0.01-0.1) |
| Maldives | 30.46 (20.62-42.82) | 58.61 (44.35-74.88) | 102.25 (71.33-137.25) | 59.08 (44.88-75.9) | 2.36% (2.02-2.82) | 0.03 (0.02-0.03) |
| Mali | 882.23 (595.5-1231.93) | 33.48 (25.29-43.19) | 1772.59 (1218.65-2435.06) | 33.67 (25.57-43.44) | 1.01% (0.88-1.16) | 0.03 (0.02-0.04) |
| Malta | 196.97 (135.61-277.97) | 66.54 (50.4-85.95) | 391.35 (268.19-551.78) | 60.81 (46.15-78.24) | 0.99% (0.85-1.12) | -0.11 (-0.18--0.05) |
| Marshall Islands | 5.96 (4.02-8.23) | 56.89 (42.94-73) | 11.62 (7.78-16.65) | 58.74 (44.47-75.36) | 0.95% (0.76-1.13) | 0.09 (0.07-0.1) |
| Mauritania | 229.24 (154.29-319.08) | 34.53 (26.13-44.76) | 474.52 (323.27-645.81) | 35.08 (26.7-44.79) | 1.07% (0.91-1.23) | 0.05 (0.03-0.07) |
| Mauritius | 300.93 (202.69-416.54) | 60.41 (45.93-77.44) | 765.18 (524.33-1054.26) | 60.99 (46.24-77.84) | 1.54% (1.39-1.72) | 0 (-0.01-0.01) |
| Mexico | 9381.21 (6479.62-12727.64) | 32.48 (24.35-41.9) | 27000.26 (18605.29-36535.46) | 32.61 (24.42-42.11) | 1.88% (1.84-1.93) | 0.02 (0-0.03) |
| Micronesia (Federated States of) | 17.59 (11.77-24.87) | 56.98 (43.34-73.25) | 23.51 (16.18-32.63) | 57.05 (42.95-73.12) | 0.34% (0.23-0.47) | -0.03 (-0.04--0.01) |
| Monaco | 32.31 (22.44-44.34) | 68.59 (52.19-87.66) | 36.18 (24.93-50.41) | 60.34 (45.58-77.29) | 0.12% (0.05-0.2) | -0.54 (-0.59--0.49) |
| Mongolia | 402.36 (273.04-564.41) | 63.99 (48.53-81.57) | 791.18 (548.09-1081.16) | 65.97 (50.24-84.4) | 0.97% (0.83-1.14) | 0.13 (0.11-0.14) |
| Montenegro | 275.74 (191.3-377.82) | 73.71 (56.41-94.51) | 440.05 (299.21-610) | 68.05 (51.7-87) | 0.6% (0.5-0.7) | -0.23 (-0.27--0.2) |
| Morocco | 3933.74 (2680.95-5419.54) | 43.09 (32.32-55.44) | 8778.51 (6043.33-11976.14) | 42.5 (31.99-54.68) | 1.23% (1.1-1.36) | -0.07 (-0.08--0.06) |
| Mozambique | 697.03 (477.83-977.11) | 19.75 (14.98-25.61) | 1249.24 (834.08-1742.25) | 19.86 (14.97-25.64) | 0.79% (0.69-0.91) | 0 (-0.01-0.02) |
| Myanmar | 9067.6 (6163.98-12740.52) | 60.53 (45.75-78.09) | 18029.4 (12351.99-24753.21) | 59.78 (45.33-75.89) | 0.99% (0.88-1.12) | -0.03 (-0.04--0.02) |
| Namibia | 165.08 (111.33-229.88) | 32.86 (24.87-42.67) | 303.22 (207.92-416.23) | 33.71 (25.41-43.02) | 0.84% (0.69-0.97) | 0.13 (0.09-0.16) |
| Nauru | 1.04 (0.71-1.43) | 59.01 (44.78-74.78) | 1.08 (0.72-1.53) | 60.49 (45.66-78.4) | 0.04% (-0.06-0.15) | 0.06 (0.03-0.09) |
| Nepal | 3294.38 (2241.74-4600.33) | 56.47 (43.1-72.48) | 8441.94 (5745.48-11548.24) | 54.67 (41.51-69.51) | 1.56% (1.38-1.76) | -0.1 (-0.13--0.08) |
| Netherlands | 8452.33 (6358.97-11038.71) | 58.53 (47.14-71.05) | 12687.05 (8796.43-17810.51) | 55.97 (42.51-71.94) | 0.5% (0.28-0.7) | -0.28 (-0.34--0.22) |
| New Zealand | 2921.34 (1984.6-4062.67) | 100.59 (75.99-128.39) | 5169.92 (3758.28-6853.74) | 91.27 (72.31-113.39) | 0.77% (0.63-0.98) | -0.19 (-0.28--0.09) |
| Nicaragua | 301.06 (208.11-410.21) | 29.96 (22.58-38.86) | 913.89 (627.39-1245.08) | 30.5 (22.78-39.69) | 2.04% (1.85-2.24) | 0.08 (0.07-0.1) |
| Niger | 560.42 (377.63-786.72) | 33.93 (25.59-43.58) | 1553.23 (1051.06-2156.73) | 33.48 (25.2-43.05) | 1.77% (1.58-1.96) | -0.06 (-0.07--0.05) |
| Nigeria | 10020.78 (6820.27-13878.26) | 36.16 (27.57-46.27) | 18518.07 (12709.53-25349.06) | 37.13 (28.17-47.52) | 0.85% (0.81-0.9) | 0.1 (0.03-0.17) |
| Niue | 0.94 (0.66-1.27) | 58.77 (44.38-75) | 0.94 (0.65-1.28) | 61.05 (46.41-78.3) | 0% (-0.07-0.07) | 0.11 (0.08-0.13) |
| North Macedonia | 754.09 (510.76-1038.7) | 65.63 (50.23-83.45) | 1349.66 (920.11-1872.26) | 63.12 (47.72-80.61) | 0.79% (0.65-0.92) | -0.13 (-0.14--0.12) |
| Northern Mariana Islands | 4.62 (3.13-6.46) | 58.65 (44.39-75.1) | 19.46 (13.16-27.05) | 60.57 (45.86-77.16) | 3.21% (2.95-3.51) | 0.08 (0.05-0.11) |
| Norway | 3003.46 (2073.96-4172.31) | 65.59 (49.54-83.66) | 3595.5 (2475.12-5007.16) | 59.38 (45.29-75.42) | 0.2% (0.15-0.23) | -0.44 (-0.48--0.4) |
| Oman | 149.04 (101.03-209.55) | 39.82 (30.08-51.53) | 369.18 (252.5-515.13) | 42.61 (32.31-54.88) | 1.48% (1.3-1.65) | 0.3 (0.27-0.34) |
| Pakistan | 23129.63 (15829.31-31734.4) | 61.34 (46.37-79.07) | 41035.96 (27784.44-56866.99) | 61.52 (46.48-78.21) | 0.77% (0.67-0.87) | 0.02 (0.01-0.03) |
| Palau | 3.96 (2.69-5.49) | 59.08 (45.09-75.21) | 8.14 (5.52-11.43) | 61.29 (46.51-78.41) | 1.06% (0.9-1.22) | 0.1 (0.08-0.13) |
| Palestine | 234.77 (161.18-320.03) | 39.86 (29.96-51.23) | 565.42 (390.24-777.68) | 40.09 (30.41-51.31) | 1.41% (1.27-1.57) | 0.02 (0.01-0.02) |
| Panama | 310.86 (214.57-423.02) | 29.5 (22.14-38.27) | 932.94 (642.23-1264.38) | 30.77 (22.97-39.8) | 2% (1.83-2.19) | 0.18 (0.16-0.2) |
| Papua New Guinea | 625.42 (422.78-885.4) | 56.85 (43.07-72.13) | 1493.84 (1023.72-2079.26) | 58.06 (43.98-73.68) | 1.39% (1.21-1.56) | 0.06 (0.04-0.08) |
| Paraguay | 612.29 (428.78-831.9) | 40.16 (30.27-51.92) | 1537.6 (1061.41-2097.1) | 40.08 (30.31-51.88) | 1.51% (1.33-1.66) | -0.05 (-0.07--0.03) |
| Peru | 1150.5 (781.02-1556.66) | 14.24 (10.53-18.48) | 3531.59 (2443.71-4810.72) | 14.92 (11.14-19.42) | 2.07% (1.86-2.32) | 0.2 (0.16-0.24) |
| Philippines | 11254.42 (7690.12-15419.67) | 61.28 (46.33-77.93) | 30161.41 (20588.22-41503.39) | 61.56 (46.65-78.41) | 1.68% (1.62-1.73) | -0.02 (-0.04--0.01) |
| Poland | 21367.52 (14680.6-29427.6) | 75.32 (57.64-95.8) | 34612.06 (23760.93-47901.4) | 74.6 (57.04-95.05) | 0.62% (0.57-0.66) | 0.27 (0.03-0.52) |
| Portugal | 7243.96 (4993.81-10089.89) | 72.19 (54.83-92.68) | 9206.88 (6403.18-12685.91) | 60.86 (46.41-78.71) | 0.27% (0.18-0.38) | -0.7 (-0.75--0.65) |
| Puerto Rico | 860.88 (584.5-1181.18) | 30.09 (22.69-39.15) | 1780.99 (1209.15-2420.44) | 30.81 (23.01-39.89) | 1.07% (0.94-1.2) | 0.09 (0.08-0.11) |
| Qatar | 18.24 (12.44-25.24) | 42.26 (31.97-54.24) | 162.54 (106.53-232.11) | 40.83 (31.1-52.17) | 7.91% (7-8.72) | -0.12 (-0.13--0.1) |
| Republic of Korea | 4047.5 (2679.06-5777.1) | 27.61 (21.38-35.44) | 12574.78 (8417.99-17535.31) | 24.56 (18.62-31.52) | 2.11% (1.9-2.37) | -0.45 (-0.5--0.4) |
| Republic of Moldova | 1918.94 (1291.29-2683.38) | 69.54 (52.87-88.42) | 2638.11 (1806.2-3636.77) | 72.59 (55.75-92.75) | 0.37% (0.29-0.47) | 0.04 (-0.02-0.1) |
| Romania | 12728.8 (8793-17563.4) | 70.26 (53.72-89.99) | 15971.14 (10853.5-22147.42) | 66.84 (51.05-85.75) | 0.25% (0.17-0.34) | -0.64 (-0.8--0.48) |
| Russian Federation | 77698.58 (53371.53-105750.15) | 67.48 (51.66-86.09) | 113370.36 (77564.63-156946.57) | 73.58 (56.52-94.37) | 0.46% (0.41-0.51) | 0.36 (0.33-0.39) |
| Rwanda | 341.32 (231.31-479.1) | 19.45 (14.64-25.35) | 663.76 (452.73-914.51) | 18.88 (14.32-24.37) | 0.94% (0.82-1.09) | -0.12 (-0.13--0.11) |
| Saint Kitts and Nevis | 9.63 (6.55-13.28) | 29.42 (22.13-37.88) | 13.38 (9.18-18.48) | 30.29 (22.64-39.61) | 0.39% (0.24-0.55) | 0.12 (0.11-0.13) |
| Saint Lucia | 20.73 (14.14-28.29) | 30.19 (22.55-39.05) | 47.61 (32.71-64.48) | 30.4 (22.77-39.28) | 1.3% (1.16-1.44) | 0.01 (0-0.02) |
| Saint Vincent and the Grenadines | 16.22 (11.15-22.19) | 28.68 (21.4-37.25) | 30.85 (21.2-41.85) | 30.51 (22.8-39.5) | 0.9% (0.78-1.03) | 0.23 (0.22-0.24) |
| Samoa | 35.61 (24.06-49.27) | 60.78 (45.99-77.62) | 57.48 (39.02-78.63) | 62.27 (46.94-79.98) | 0.61% (0.5-0.71) | 0.07 (0.05-0.09) |
| San Marino | 15.77 (10.89-22.02) | 68.61 (52.07-88.07) | 23.29 (15.93-32.06) | 59.74 (45.01-76.68) | 0.48% (0.39-0.59) | -0.56 (-0.59--0.52) |
| Sao Tome and Principe | 16.29 (11-22.54) | 36.05 (27.15-46.11) | 23.3 (16.08-32.04) | 37.36 (28.27-48.03) | 0.43% (0.35-0.53) | 0.07 (0.01-0.12) |
| Saudi Arabia | 1350.38 (926.35-1838.55) | 39.45 (29.83-50.49) | 3339.42 (2264.46-4630.67) | 39.79 (30.1-50.76) | 1.47% (1.2-1.7) | 0.03 (0-0.05) |
| Senegal | 744.57 (505.2-1037.84) | 35.53 (26.66-45.86) | 1658.37 (1130.98-2284.21) | 34.94 (26.49-44.97) | 1.23% (1.06-1.41) | -0.06 (-0.07--0.05) |
| Serbia | 4772.93 (3256.31-6675.84) | 63.31 (48.02-81.02) | 6814.35 (4670-9369.51) | 62.65 (47.59-80.41) | 0.43% (0.31-0.56) | -0.29 (-0.42--0.16) |
| Seychelles | 25.85 (17.74-35.1) | 62.3 (46.89-79.26) | 44.12 (30.86-60.2) | 61.61 (46.92-78.48) | 0.71% (0.6-0.83) | -0.07 (-0.08--0.05) |
| Sierra Leone | 502.11 (340.19-695.64) | 38.53 (29.32-49.42) | 830.14 (559.82-1148.32) | 37.62 (28.33-48.49) | 0.65% (0.56-0.78) | -0.13 (-0.16--0.09) |
| Singapore | 311.98 (209.58-439.64) | 27.42 (21-34.92) | 1111.78 (743.36-1574.58) | 25.58 (19.54-32.81) | 2.56% (2.32-2.87) | -0.28 (-0.31--0.25) |
| Slovakia | 3069.45 (2065.13-4280.49) | 80.14 (61.36-103.1) | 4517.07 (3048.43-6343.62) | 75.46 (57.57-96.25) | 0.47% (0.39-0.58) | 0.19 (-0.05-0.43) |
| Slovenia | 1044.34 (719.44-1423.52) | 69.66 (53.24-88.81) | 1715.16 (1181.54-2368.96) | 66.05 (50.5-85.08) | 0.64% (0.52-0.76) | -0.22 (-0.24--0.19) |
| Solomon Islands | 44.45 (29.67-64.08) | 57.24 (43.42-73.33) | 95.89 (64.78-133.5) | 57.9 (43.67-73.72) | 1.16% (0.99-1.32) | 0.03 (0.02-0.04) |
| Somalia | 227.29 (154.47-315.97) | 18.3 (13.91-23.7) | 665.94 (444.64-949.97) | 18.56 (14.06-24.18) | 1.93% (1.69-2.19) | 0.04 (0.03-0.05) |
| South Africa | 5249.55 (3604.26-7172.4) | 38.72 (29.36-49.74) | 11135.29 (7641.57-15171) | 37.98 (28.89-48.71) | 1.12% (1.06-1.18) | -0.06 (-0.07--0.05) |
| South Sudan | 277.03 (188.29-382.56) | 18.66 (14.15-24.18) | 409.18 (279.49-561.14) | 19.34 (14.59-25.02) | 0.48% (0.37-0.57) | 0.11 (0.09-0.14) |
| Spain | 28016.47 (19578.04-39010.07) | 71.89 (54.63-92.96) | 35012.91 (24761.52-48140.96) | 59.99 (45.96-76.33) | 0.25% (0.17-0.33) | -0.64 (-0.8--0.47) |
| Sri Lanka | 4073.55 (2781.52-5626.71) | 58.29 (44.15-74.6) | 11094.31 (7547.44-15383.86) | 59.37 (45.01-75.77) | 1.72% (1.57-1.88) | 0.07 (0.06-0.08) |
| Sudan | 2518.72 (1709.56-3449.83) | 40.39 (30.36-51.77) | 4780.54 (3302.5-6513.1) | 41.83 (31.36-53.78) | 0.9% (0.79-1.01) | 0.12 (0.12-0.13) |
| Suriname | 51.64 (35.81-70.28) | 29.32 (21.87-38.18) | 127.48 (86.78-173.91) | 30.38 (22.66-39.25) | 1.47% (1.29-1.63) | 0.15 (0.13-0.17) |
| Sweden | 8994.88 (6241.3-12457.87) | 92.48 (71.12-117.15) | 11034.88 (7563.87-15563.85) | 88.44 (67.69-112.96) | 0.23% (0.15-0.3) | 0.29 (0.01-0.57) |
| Switzerland | 3953.48 (2722.41-5410.76) | 57.72 (44.28-73.67) | 5467.48 (3770.72-7533.24) | 50.94 (38.68-65.54) | 0.38% (0.3-0.48) | -0.15 (-0.44-0.15) |
| Syrian Arab Republic | 1350.27 (927.15-1863.76) | 41.16 (31.04-53.38) | 3352.07 (2289.1-4605.38) | 41.11 (30.95-52.75) | 1.48% (1.31-1.65) | -0.03 (-0.04--0.02) |
| Taiwan (Province of China) | 5938.96 (4013.21-8338.09) | 55.01 (41.6-70.45) | 15324.44 (11042.38-20035.08) | 52.39 (41.15-64.85) | 1.58% (1.31-1.93) | -0.2 (-0.23--0.18) |
| Tajikistan | 1017.87 (707.95-1373.88) | 61.04 (46.77-77.14) | 1657.82 (1128.98-2275.58) | 61.77 (47.3-78.34) | 0.63% (0.46-0.77) | 0.02 (0-0.03) |
| Thailand | 12524.9 (8555.88-17093.17) | 57.03 (43.21-72.57) | 42308.91 (29098.87-57319.17) | 58.36 (44.07-74.5) | 2.38% (2.14-2.63) | 0.07 (0.07-0.08) |
| Timor-Leste | 85.24 (58.31-120.9) | 59.21 (44.8-76.64) | 340.91 (230.95-478.18) | 58.64 (44.42-74.85) | 3% (2.68-3.3) | -0.02 (-0.03--0.01) |
| Togo | 259.53 (177.21-361.59) | 35.18 (26.53-45.49) | 717.82 (478.32-998) | 34.45 (25.83-44.45) | 1.77% (1.57-1.95) | -0.08 (-0.09--0.07) |
| Tokelau | 0.63 (0.43-0.85) | 58.57 (44.81-75.06) | 0.57 (0.39-0.8) | 60.94 (46.48-78.56) | -0.09% (-0.16--0.02) | 0.12 (0.1-0.14) |
| Tonga | 22.34 (15.18-30.69) | 60.36 (45.87-77.26) | 33.78 (23.2-45.75) | 62.94 (47.2-80.39) | 0.51% (0.39-0.65) | 0.13 (0.1-0.16) |
| Trinidad and Tobago | 193.77 (131.34-265.19) | 30.74 (22.94-39.97) | 439.74 (298.53-603.81) | 31.19 (23.24-40.17) | 1.27% (1.1-1.42) | 0.08 (0.07-0.09) |
| Tunisia | 1384.22 (949.54-1893.46) | 39.94 (30.01-51.41) | 3635.69 (2507.02-4995.12) | 40.51 (30.45-52.06) | 1.63% (1.46-1.81) | 0.05 (0.04-0.06) |
| Turkey | 10065.63 (6975.42-13649.36) | 43.33 (32.8-55.63) | 26758.55 (18473.43-36306.46) | 42.63 (32.1-54.55) | 1.66% (1.49-1.86) | -0.36 (-0.45--0.27) |
| Turkmenistan | 713.81 (490.07-982.08) | 62.14 (47.22-80.14) | 1441.84 (992.2-1963.45) | 65.43 (49.85-83.6) | 1.02% (0.88-1.18) | 0.16 (0.14-0.17) |
| Tuvalu | 2.76 (1.84-3.92) | 58.5 (44.54-75.13) | 4.3 (2.92-6.02) | 60.55 (45.76-77.78) | 0.56% (0.44-0.75) | 0.1 (0.08-0.12) |
| Uganda | 777.88 (526.31-1068.67) | 19.59 (14.93-25.17) | 1516 (1034.41-2080.57) | 18.97 (14.43-24.53) | 0.95% (0.81-1.08) | -0.12 (-0.13--0.11) |
| Ukraine | 33266.8 (22510.78-45750.86) | 70.71 (53.75-89.52) | 38038.18 (26084.54-52166.13) | 76.81 (59.3-98.35) | 0.14% (0.08-0.22) | 0.37 (0.32-0.41) |
| United Arab Emirates | 71.93 (48.36-101.41) | 44 (33.32-56.23) | 507.95 (330.98-716.6) | 41.75 (31.45-53.07) | 6.06% (5.03-6.97) | -0.19 (-0.22--0.16) |
| United Kingdom | 47586.78 (32865.33-66096.71) | 77.47 (58.47-99) | 58282.35 (42295.3-78276.44) | 75.05 (59.02-93.07) | 0.22% (0.17-0.31) | -0.03 (-0.12-0.07) |
| United Republic of Tanzania | 1473.04 (978.92-2057.99) | 21.65 (16.4-28.22) | 3270.4 (2210-4521.76) | 22.26 (16.88-29) | 1.22% (1.08-1.39) | -0.06 (-0.18-0.05) |
| United States of America | 239249.98 (165751.1-322159.47) | 94.68 (72.49-120.11) | 494178.27 (378533.56-626078.98) | 109.52 (88.97-131.92) | 1.07% (0.84-1.37) | 1.51 (1.07-1.95) |
| United States Virgin Islands | 16.97 (11.53-23.37) | 29.65 (21.93-38.5) | 46.33 (31.21-64.18) | 29.94 (22.46-38.63) | 1.73% (1.54-1.92) | 0.02 (0.01-0.03) |
| Uruguay | 1272.76 (868.69-1749.79) | 42.1 (31.89-54.6) | 1699.11 (1165.34-2318.87) | 42.56 (32.27-55.3) | 0.33% (0.24-0.42) | 0.02 (0-0.04) |
| Uzbekistan | 3974.75 (2732.95-5390.85) | 58.46 (45.01-75.09) | 7152.73 (4766.73-10013.6) | 62.18 (47.57-78.93) | 0.8% (0.6-0.99) | 0.23 (0.22-0.25) |
| Vanuatu | 26.28 (17.73-37.04) | 64.07 (48.96-81.37) | 73.13 (49.43-102.61) | 64.46 (48.86-82.64) | 1.78% (1.59-2.06) | 0 (-0.01-0.01) |
| Venezuela (Bolivarian Republic of) | 2161.24 (1489.71-2934.81) | 33.03 (24.74-42.53) | 6663.58 (4554.64-9095.52) | 32.04 (24.04-41.7) | 2.08% (1.86-2.31) | -0.09 (-0.12--0.07) |
| Viet Nam | 16026.03 (11015.21-22005.06) | 57.56 (43.86-73.87) | 37362.03 (25570.43-51083.88) | 63.3 (48.03-80.39) | 1.33% (1.17-1.49) | 0.44 (0.39-0.49) |
| Yemen | 1202.3 (818.19-1661.69) | 40 (30.22-51.22) | 3262.49 (2247.82-4559.1) | 39.69 (30.03-51.24) | 1.71% (1.54-1.87) | -0.02 (-0.04-0) |
| Zambia | 312.3 (215.77-431.55) | 19.02 (14.37-24.65) | 692.03 (471.71-950.9) | 18.8 (14.21-24.21) | 1.22% (1.08-1.37) | -0.08 (-0.1--0.07) |
| Zimbabwe | 860.95 (585.17-1212.65) | 33.16 (24.98-42.56) | 1419.71 (960.77-1959.98) | 33.09 (24.99-42.3) | 0.65% (0.54-0.8) | -0.02 (-0.04-0.01) |
