# Supplementary material for: Estimates of the global, regional, and national burden of atrial fibrillation in older adults from 1990 to 2019: insights from the Global Burden of Disease study 2019
Source: Front Public Health. 2023 Jun 12;11:1137230. doi: 10.3389/fpubh.2023.1137230 (PMC10291625; doi:10.3389/fpubh.2023.1137230)
Supplement: Supplementary Table 2 — The deaths of atrial fibrillation between 1990 and 2019 at national level. [file Table_2.DOCX]

Supplementary Table2. The deaths of atrial fibrillation between 1990 and 2019 at national level.

| location | Case in 1990 | ASMR in 1990 | Case in 2019 | ASMR in 2019 | Percentage change | EAPC (95%) |
| --- | --- | --- | --- | --- | --- | --- |
| Afghanistan | 95.16 (61.31-128.24) | 2.69 (1.69-3.6) | 168.74 (120.89-232.36) | 3.2 (2.3-4.23) | 0.77% (0.38-1.3) | 0.68 (0.62-0.74) |
| Albania | 42.04 (35.23-46.79) | 3.76 (3.21-4.2) | 121.12 (92.11-154.69) | 3.97 (3.01-4.98) | 1.88% (1.2-2.64) | 0.23 (0.15-0.31) |
| Algeria | 223.19 (175.44-290.69) | 4.79 (3.69-6.43) | 826.38 (632.96-1064.26) | 4.87 (3.67-6.11) | 2.7% (1.84-3.88) | 0.23 (0.17-0.29) |
| American Samoa | 0.48 (0.4-0.58) | 4.83 (4-5.85) | 1.28 (1.03-1.58) | 4.92 (3.92-6.14) | 1.66% (1.15-2.31) | 0.14 (0.07-0.21) |
| Andorra | 1.65 (1.2-2.39) | 5.61 (4.23-7.72) | 5.58 (3.6-7.68) | 5.78 (3.83-7.76) | 2.39% (1.25-3.74) | 0.17 (0.04-0.29) |
| Angola | 61.39 (40.08-84.43) | 3.83 (2.44-5.42) | 234.46 (167.55-297.82) | 5.19 (3.66-6.61) | 2.82% (1.86-4.29) | 1.03 (1-1.06) |
| Antigua and Barbuda | 2.39 (1.98-2.94) | 5.85 (4.76-7.24) | 4.11 (3.4-5.29) | 7.8 (6.45-9.95) | 0.72% (0.43-1.03) | 0.86 (0.71-1) |
| Argentina | 942.14 (797.67-1137.49) | 4.74 (3.93-5.63) | 1920.93 (1627.58-2462.86) | 5.14 (4.25-6.59) | 1.04% (0.78-1.3) | 0.21 (0.07-0.36) |
| Armenia | 43.52 (35.95-53.27) | 2.95 (2.42-3.76) | 124.54 (101.36-162.08) | 4.35 (3.53-5.98) | 1.86% (1.27-2.46) | 1.28 (1.12-1.45) |
| Australia | 969.23 (776.55-1091.75) | 7.48 (5.93-8.35) | 2100.91 (1670.8-2546.32) | 6.79 (5.41-8.24) | 1.17% (0.96-1.51) | -0.51 (-0.58--0.44) |
| Austria | 541.85 (467.2-838.25) | 5.79 (4.93-9.06) | 886.08 (682.97-1007.32) | 6.27 (4.89-7.12) | 0.64% (0.04-0.9) | 0.29 (0.07-0.5) |
| Azerbaijan | 88.48 (61.03-109.24) | 3.5 (2.18-4.52) | 196.74 (164.38-229.42) | 5.69 (4.57-6.67) | 1.22% (0.67-2.34) | 2.19 (1.93-2.44) |
| Bahamas | 4.68 (3.8-5.75) | 5.81 (4.7-7.17) | 12.49 (10.05-16.51) | 6.32 (5.06-8.22) | 1.67% (1.2-2.24) | 0.3 (0.09-0.51) |
| Bahrain | 3.03 (2.58-4.03) | 4.61 (3.88-6.68) | 29.71 (21.88-36.85) | 10.65 (8.28-12.78) | 8.82% (4.69-11.88) | 4.5 (3.78-5.22) |
| Bangladesh | 759.87 (485.19-1024.98) | 3.32 (2.09-4.48) | 3199.44 (2043.75-4279.71) | 4.73 (2.93-6.37) | 3.21% (1.88-4.9) | 1.47 (1.2-1.74) |
| Barbados | 11.17 (9.22-13.87) | 4.94 (4.07-6.16) | 18.35 (14.8-23.68) | 5.71 (4.59-7.19) | 0.64% (0.34-0.93) | 0.46 (0.37-0.55) |
| Belarus | 288.08 (233.88-372.04) | 3.29 (2.64-4.23) | 529.2 (413.87-682.22) | 4.5 (3.51-5.78) | 0.84% (0.34-1.35) | 1.02 (0.93-1.11) |
| Belgium | 689.7 (596.49-963.42) | 5.87 (5.01-7.89) | 1070.35 (877.49-1375.57) | 5.71 (4.61-7.2) | 0.55% (0.3-0.74) | -0.2 (-0.27--0.14) |
| Belize | 2.13 (1.67-2.87) | 3.68 (2.85-4.89) | 6.31 (5.13-8.23) | 4.53 (3.56-5.89) | 1.96% (1.17-2.62) | 0.58 (0.15-1) |
| Benin | 40.63 (28.93-52.3) | 3.8 (2.67-4.93) | 102.36 (81.22-127.93) | 4.63 (3.65-5.68) | 1.52% (0.93-2.4) | 0.74 (0.62-0.86) |
| Bermuda | 2.18 (1.72-2.78) | 5.94 (4.65-7.54) | 3.98 (3.08-5.19) | 4.69 (3.67-6) | 0.82% (0.49-1.29) | -0.92 (-1--0.84) |
| Bhutan | 2.96 (1.88-4.23) | 2.99 (1.87-4.29) | 15.19 (9.98-21.37) | 4.79 (3.15-6.75) | 4.12% (2.2-6.5) | 1.78 (1.71-1.85) |
| Bolivia (Plurinational State of) | 88.55 (62.82-127.28) | 5.2 (3.75-7.14) | 331.31 (246.81-457.75) | 6.75 (4.95-8.92) | 2.74% (1.88-3.83) | 0.87 (0.84-0.9) |
| Bosnia and Herzegovina | 88.32 (75.03-101.79) | 4.24 (3.47-4.82) | 238.04 (185.85-317.68) | 5.75 (4.53-7.48) | 1.7% (1.11-2.53) | 1.1 (0.66-1.55) |
| Botswana | 7.78 (5.89-9.77) | 3.28 (2.5-4.15) | 21.32 (15.31-28.54) | 3.8 (2.71-5.21) | 1.74% (0.88-2.84) | 0.19 (-0.05-0.43) |
| Brazil | 2139.53 (1819.56-2688.22) | 4.78 (3.98-6.04) | 7445.83 (5939.96-8716.31) | 5.04 (4.01-5.97) | 2.48% (1.91-2.75) | 0.52 (0.33-0.71) |
| Brunei Darussalam | 3.12 (2.57-3.89) | 7.63 (6.37-9.26) | 8.71 (7.59-9.96) | 8.1 (6.99-9.22) | 1.79% (1.17-2.44) | 0.37 (0.2-0.53) |
| Bulgaria | 390.28 (323.76-650.13) | 5.42 (4.47-8.77) | 751.59 (578.53-1182.41) | 6.41 (4.99-9.99) | 0.93% (0.53-1.41) | 0.27 (0.09-0.45) |
| Burkina Faso | 70.96 (47.53-96.35) | 3.85 (2.55-5.27) | 233.2 (170.03-309.28) | 5.58 (4.09-7.44) | 2.29% (1.51-3.33) | 1.72 (1.51-1.93) |
| Burundi | 59.75 (34.79-83.94) | 4.69 (2.65-6.76) | 74.17 (44.97-100.28) | 3.87 (2.28-5.24) | 0.24% (-0.1-0.71) | -0.86 (-0.98--0.74) |
| Cabo Verde | 5.91 (4.53-6.98) | 3.37 (2.59-4.02) | 17.61 (13.59-21.83) | 6.29 (4.87-7.7) | 1.98% (1.22-3.32) | 1.67 (1.43-1.92) |
| Cambodia | 44.12 (34.03-59.26) | 2.25 (1.73-2.9) | 179.21 (142.51-224.88) | 3.16 (2.47-3.94) | 3.06% (1.98-4.22) | 1.17 (1.12-1.23) |
| Cameroon | 87.13 (61.78-117.37) | 4.82 (3.41-6.37) | 277.83 (215.15-350.45) | 5.78 (4.46-7.24) | 2.19% (1.27-3.63) | 0.65 (0.61-0.68) |
| Canada | 962.9 (791.81-1192.23) | 4.35 (3.51-5.3) | 1992.17 (1594.8-2458.81) | 3.97 (3.09-4.84) | 1.07% (0.88-1.3) | -0.67 (-0.8--0.54) |
| Central African Republic | 22.31 (13.32-31.09) | 4.78 (2.82-6.72) | 40.62 (25.05-56.33) | 4.89 (2.94-6.68) | 0.82% (0.39-1.47) | 0.12 (0.06-0.18) |
| Chad | 53.25 (32.05-73.33) | 3.7 (2.23-5.11) | 105.51 (75.75-140.72) | 4.25 (3.07-5.63) | 0.98% (0.51-1.71) | 0.57 (0.51-0.63) |
| Chile | 319.15 (277.32-453.43) | 5.85 (4.99-7.95) | 950.91 (804.55-1353.83) | 5.97 (4.91-8.69) | 1.98% (1.71-2.23) | 0.33 (0.21-0.45) |
| China | 13734.95 (11532.83-16142.93) | 4.04 (3.33-4.7) | 39970.2 (33722.12-46387.23) | 3.84 (3.2-4.45) | 1.91% (1.34-2.61) | -0.28 (-0.33--0.22) |
| Colombia | 310.78 (264.86-460.76) | 3.5 (2.9-5.12) | 1289.83 (952.45-1685.86) | 3.64 (2.64-4.76) | 3.15% (1.94-4.3) | 0.05 (-0.1-0.2) |
| Comoros | 5.59 (3.53-7.4) | 4.44 (2.79-5.89) | 12.66 (8.81-16.05) | 4.43 (3.04-5.71) | 1.26% (0.71-2.09) | -0.04 (-0.15-0.06) |
| Congo | 29.47 (19.76-38.93) | 6.06 (4-8.11) | 72.32 (55.01-93.47) | 6.34 (4.75-8.2) | 1.45% (0.86-2.33) | 0.14 (0.03-0.25) |
| Cook Islands | 0.34 (0.28-0.43) | 4.89 (3.93-6.08) | 0.8 (0.64-0.98) | 4.79 (3.85-5.82) | 1.32% (0.79-2.11) | 0 (-0.1-0.09) |
| Costa Rica | 34.71 (26.55-40.28) | 3.32 (2.58-3.94) | 120.31 (84.9-154.39) | 3.82 (2.69-4.88) | 2.47% (1.79-3.3) | 0.1 (-0.11-0.32) |
| Croatia | 160.42 (138.85-202.24) | 3.85 (3.28-4.82) | 243.98 (189.28-337.51) | 3.39 (2.65-4.62) | 0.52% (0.21-0.89) | -0.18 (-0.29--0.06) |
| Cuba | 275.58 (223.34-353.58) | 4.02 (3.2-5.13) | 542.78 (423.61-713.66) | 4.27 (3.32-5.68) | 0.97% (0.58-1.45) | 0.04 (-0.13-0.2) |
| Cyprus | 44.74 (36.83-55.14) | 9.5 (7.79-11.69) | 105.37 (89.77-119.81) | 8.14 (6.79-9.25) | 1.36% (0.83-1.99) | -0.71 (-0.86--0.56) |
| Czechia | 433.95 (372.85-565.6) | 4.26 (3.66-5.58) | 677.64 (507.02-838.53) | 4.17 (3.17-5.09) | 0.56% (0.15-0.95) | -0.08 (-0.15--0.01) |
| C么te d'Ivoire | 51.25 (37.6-67.08) | 4.16 (3.11-5.3) | 177.45 (141.86-219.96) | 4.59 (3.52-5.73) | 2.46% (1.62-3.61) | 0.39 (0.34-0.45) |
| Democratic People's Republic of Korea | 257.64 (199-325.74) | 3.93 (2.86-4.99) | 717.58 (574.23-867.42) | 3.85 (3.02-4.74) | 1.79% (1.22-2.54) | -0.12 (-0.28-0.04) |
| Democratic Republic of the Congo | 331.54 (176.02-623.42) | 5.2 (2.71-9.44) | 933.59 (542.89-1448.37) | 5.65 (3.27-8.81) | 1.82% (1.01-2.97) | 0.25 (0.14-0.37) |
| Denmark | 383.56 (327.94-486.79) | 5.87 (4.94-7.38) | 583.39 (470.4-696.6) | 6.73 (5.42-7.98) | 0.52% (0.33-0.71) | 0.37 (0.18-0.56) |
| Djibouti | 2.19 (1.25-3.05) | 4.56 (2.54-6.4) | 10.5 (6.41-14.14) | 4.73 (2.88-6.36) | 3.8% (2.56-5.81) | 0.15 (0.05-0.26) |
| Dominica | 3.87 (3.27-4.42) | 7.18 (6.06-8.1) | 4.91 (4.08-5.97) | 7.9 (6.54-9.56) | 0.27% (0.01-0.59) | 0.53 (0.43-0.63) |
| Dominican Republic | 82.76 (65.31-97.17) | 4.35 (3.52-5.13) | 303.46 (237.88-383.1) | 5.52 (4.32-6.81) | 2.67% (1.87-3.69) | 1.69 (1.41-1.98) |
| Ecuador | 118.85 (102.58-150.09) | 3.74 (3.19-4.8) | 439.92 (347.57-550.93) | 5.57 (4.47-6.82) | 2.7% (1.9-3.74) | 2.13 (1.79-2.47) |
| Egypt | 447.49 (340.38-587.88) | 3.36 (2.49-4.38) | 1067.94 (700.3-1472.23) | 3.93 (2.51-5.38) | 1.39% (0.75-2.18) | 0.68 (0.49-0.87) |
| El Salvador | 57.78 (46.6-65.39) | 3.56 (2.69-4.03) | 165.15 (123.94-206.81) | 4.14 (3.09-5.14) | 1.86% (1.18-2.62) | 0.36 (0.25-0.47) |
| Equatorial Guinea | 3.87 (2.31-5.6) | 4.12 (2.43-6.03) | 15.94 (10.36-23.61) | 6.96 (4.49-10.58) | 3.12% (1.35-5.71) | 2.05 (1.97-2.13) |
| Eritrea | 14.33 (8.53-22.81) | 4.22 (2.42-6.99) | 53.44 (33.88-72.7) | 5.43 (3.4-7.54) | 2.73% (1.52-4.6) | 0.66 (0.51-0.81) |
| Estonia | 54.42 (47.64-72.25) | 3.69 (3.16-5.03) | 103.55 (79.94-143.56) | 4.58 (3.59-6.43) | 0.9% (0.48-1.37) | 0.78 (0.68-0.87) |
| Eswatini | 3.7 (2.82-4.61) | 2.93 (2.24-3.62) | 8.61 (6.28-11.2) | 3.71 (2.67-4.89) | 1.33% (0.68-2.36) | 0.98 (0.73-1.23) |
| Ethiopia | 309.97 (165.63-451.42) | 4.02 (2.04-5.99) | 846.15 (494.47-1117.93) | 4 (2.31-5.29) | 1.73% (0.89-3.21) | -0.12 (-0.29-0.04) |
| Fiji | 6.49 (5.25-7.91) | 4.43 (3.62-5.4) | 17.44 (14.3-21.59) | 5.22 (4.3-6.32) | 1.69% (1-2.6) | 0.63 (0.53-0.73) |
| Finland | 272.49 (176.35-311.48) | 5.1 (3.33-5.84) | 470.08 (295.66-540.97) | 4.77 (3.08-5.48) | 0.73% (0.51-0.99) | -0.22 (-0.3--0.14) |
| France | 3650.32 (2943.8-4211.17) | 5.45 (4.47-6.3) | 5107.1 (3738.66-6007.71) | 4.59 (3.44-5.39) | 0.4% (0.19-0.59) | -0.68 (-0.74--0.63) |
| Gabon | 18.59 (12.03-26.7) | 6.11 (3.88-8.47) | 37.34 (26.57-49.16) | 6.92 (4.87-9.21) | 1.01% (0.47-1.79) | 0.33 (0.28-0.39) |
| Gambia | 5.41 (3.78-6.91) | 3.86 (2.65-4.91) | 25.09 (19.66-31.58) | 5.21 (4.06-6.57) | 3.64% (2.51-5.46) | 1.05 (1-1.1) |
| Georgia | 162.86 (127.89-221.77) | 4.22 (3.13-6.2) | 309.53 (234.55-369.43) | 6.03 (4.74-7.17) | 0.9% (0.35-1.42) | 2.07 (1.51-2.64) |
| Germany | 6375.99 (5060.03-10776.52) | 6.05 (4.81-10.32) | 12588.28 (10325.86-15315.88) | 7.42 (6.07-9.21) | 0.97% (0.32-1.45) | 1.26 (0.97-1.55) |
| Ghana | 74.06 (60.13-88.62) | 3.23 (2.64-3.89) | 260.17 (212.26-323.48) | 3.93 (3.19-4.77) | 2.51% (1.65-3.57) | 0.69 (0.64-0.73) |
| Greece | 614.52 (518.69-1033) | 5.46 (4.51-9.41) | 1178.89 (939.34-2014.16) | 5.23 (4.12-8.96) | 0.92% (0.73-1.21) | -0.32 (-0.4--0.23) |
| Greenland | 1.5 (1.27-1.72) | 9.79 (8.16-11.27) | 3.53 (2.82-4.18) | 9.35 (7.48-10.94) | 1.35% (0.85-1.9) | -0.38 (-0.54--0.23) |
| Grenada | 3.23 (2.55-3.95) | 5.41 (4.32-6.55) | 3.38 (2.86-4.5) | 5.94 (5.01-7.87) | 0.04% (-0.17-0.29) | 0.22 (0.06-0.37) |
| Guam | 1.77 (1.52-2.11) | 5.89 (4.96-7.15) | 3.56 (2.93-4.28) | 3.1 (2.52-3.72) | 1.01% (0.58-1.55) | -2.37 (-2.87--1.87) |
| Guatemala | 58.82 (43.34-70.54) | 3.93 (2.9-4.96) | 217.01 (159.71-270.98) | 3.72 (2.74-4.49) | 2.69% (1.91-3.63) | -0.88 (-1.09--0.66) |
| Guinea | 65.75 (43.55-86.96) | 3.98 (2.64-5.32) | 126.61 (96.51-162.99) | 4.62 (3.53-5.92) | 0.93% (0.43-1.64) | 0.63 (0.52-0.73) |
| Guinea-Bissau | 6.63 (4.6-9.02) | 4.12 (2.85-5.53) | 13.2 (9.99-17.55) | 4.95 (3.73-6.54) | 0.99% (0.41-1.87) | 0.73 (0.69-0.77) |
| Guyana | 11.07 (9.38-15.08) | 5.56 (4.69-7.55) | 18.46 (14.34-24.56) | 6.54 (5.12-8.65) | 0.67% (0.33-1.08) | 0.34 (0.1-0.59) |
| Haiti | 88.86 (62.37-122.94) | 6.62 (4.2-9.35) | 182.87 (128.99-271.19) | 5.79 (4.19-8.31) | 1.06% (0.53-1.88) | -0.43 (-0.49--0.36) |
| Honduras | 38.26 (23.85-65.53) | 3.78 (2.18-6.84) | 174.62 (133.4-223.75) | 5.74 (4.27-7.56) | 3.56% (2.2-5.32) | 1.62 (1.33-1.92) |
| Hungary | 470.62 (408.31-582.02) | 4.65 (3.96-5.8) | 644.41 (518.35-817.91) | 4.21 (3.36-5.37) | 0.37% (0.16-0.6) | -0.44 (-0.5--0.39) |
| Iceland | 13.93 (11.9-18.45) | 6.14 (5.17-8.16) | 23.3 (18.51-29.86) | 5.78 (4.58-7.4) | 0.67% (0.46-0.88) | -0.24 (-0.31--0.16) |
| India | 5549.18 (4198.87-7331.3) | 3.34 (2.49-4.33) | 23386.64 (18133.68-30007.28) | 3.99 (3.07-5.12) | 3.21% (2.08-4.52) | 0.45 (0.32-0.58) |
| Indonesia | 1145.14 (948.48-1390.17) | 2.82 (2.33-3.39) | 3865.15 (3042.64-5105.53) | 4.33 (3.37-5.65) | 2.38% (1.7-3.12) | 1.47 (1.41-1.53) |
| Iran (Islamic Republic of) | 272.75 (190.33-329.25) | 3.11 (2.1-3.79) | 1283.11 (1119.85-1463.17) | 3.16 (2.68-3.59) | 3.7% (2.79-5.9) | 0.12 (-0.02-0.25) |
| Iraq | 126.82 (91.73-217.64) | 3.44 (2.49-5.35) | 416.2 (312.42-714.16) | 4.43 (3.37-7.52) | 2.28% (1.5-3.41) | 0.93 (0.79-1.08) |
| Ireland | 193.93 (160.82-230.95) | 6.39 (5.37-7.68) | 326.19 (235.16-373.48) | 6.08 (4.38-6.95) | 0.68% (0.35-0.9) | -0.39 (-0.54--0.23) |
| Israel | 209.16 (181.59-270.26) | 5.97 (5.09-7.87) | 402.91 (338.18-549.66) | 4.8 (3.91-6.44) | 0.93% (0.74-1.24) | -1.17 (-1.31--1.03) |
| Italy | 3892.28 (3397.93-5988.44) | 6.01 (5.05-9.52) | 6480.66 (5148.1-8776.32) | 5.23 (4.22-7.07) | 0.67% (0.34-0.88) | -0.58 (-0.66--0.5) |
| Jamaica | 51.65 (42.95-67.77) | 4.32 (3.48-5.56) | 100.26 (76.32-147.88) | 5.3 (4.03-7.38) | 0.94% (0.48-1.41) | 0.51 (0.33-0.7) |
| Japan | 3341.25 (2791.58-4339.54) | 3.01 (2.51-4.13) | 7580.53 (5699.25-10086.32) | 2.39 (1.83-3.27) | 1.27% (0.96-1.62) | -0.85 (-0.96--0.75) |
| Jordan | 22.76 (18.01-27.69) | 4.47 (3.37-5.49) | 102.75 (84.69-123.25) | 4.39 (3.44-5.19) | 3.51% (2.53-4.9) | -0.18 (-0.35--0.01) |
| Kazakhstan | 280.45 (211.91-521.56) | 4.12 (3-7.16) | 544.8 (419.13-1002.18) | 6.64 (5.18-11.75) | 0.94% (0.4-1.41) | 1.05 (0.78-1.32) |
| Kenya | 148.13 (94.99-195.72) | 3.46 (2.16-4.61) | 479.05 (315.51-632.93) | 4.99 (3.23-6.74) | 2.23% (1.6-3.31) | 1.45 (1.36-1.53) |
| Kiribati | 0.68 (0.52-0.92) | 4.12 (3.16-5.63) | 1.13 (0.88-1.47) | 4.29 (3.36-5.47) | 0.68% (0.28-1.2) | 0.12 (0.07-0.18) |
| Kuwait | 5.21 (4.06-6.52) | 2.25 (1.78-2.81) | 24.68 (18.9-34.52) | 2.21 (1.67-3.04) | 3.74% (2.91-4.88) | 0.33 (0-0.66) |
| Kyrgyzstan | 51.66 (41.25-58.91) | 2.57 (2-2.97) | 80.38 (62.45-94.08) | 3.67 (2.78-4.26) | 0.56% (0.37-0.75) | 1.42 (0.78-2.07) |
| Lao People's Democratic Republic | 21.51 (15.71-29.35) | 2.59 (1.86-3.49) | 67.73 (56.07-81.16) | 3.57 (2.98-4.23) | 2.15% (1.4-3.15) | 0.96 (0.9-1.03) |
| Latvia | 88.55 (74.59-105.87) | 3.54 (3-4.22) | 134.15 (108.02-167.97) | 4.11 (3.32-5.12) | 0.51% (0.26-0.8) | 0.45 (0.32-0.59) |
| Lebanon | 47.9 (37.3-59.45) | 4.05 (3.15-5.12) | 147.11 (94.42-192.34) | 4.07 (2.57-5.27) | 2.07% (0.97-3.43) | -0.08 (-0.13--0.02) |
| Lesotho | 12.44 (8.96-16.4) | 2.57 (1.83-3.43) | 21.23 (15.79-27.49) | 3.94 (2.93-5.04) | 0.71% (0.25-1.44) | 1.96 (1.77-2.16) |
| Liberia | 19.96 (14.4-25.32) | 3.91 (2.86-5.01) | 37.55 (28.08-49.11) | 4.36 (3.32-5.64) | 0.88% (0.41-1.52) | 0.59 (0.47-0.72) |
| Libya | 22.1 (15.69-30.2) | 2.28 (1.59-3.14) | 69.83 (46.34-96.99) | 2.66 (1.78-3.63) | 2.16% (1.25-3.65) | 0.79 (0.68-0.91) |
| Lithuania | 119.67 (95.6-136.98) | 3.74 (2.99-4.28) | 202.8 (162.74-251.58) | 4.18 (3.36-5.19) | 0.69% (0.41-1.07) | 0.25 (0.09-0.4) |
| Luxembourg | 26.37 (23.18-34.99) | 6.37 (5.51-8.89) | 42.87 (33.8-53.52) | 5.69 (4.45-7.19) | 0.63% (0.28-0.95) | -0.28 (-0.39--0.18) |
| Madagascar | 143.44 (85.94-188.93) | 5.41 (3.19-7.18) | 249.91 (158.97-331.8) | 5.67 (3.62-7.54) | 0.74% (0.3-1.37) | -0.07 (-0.16-0.03) |
| Malawi | 59.59 (33.94-82.19) | 3.59 (1.96-4.97) | 144.82 (86.77-191.54) | 4.18 (2.48-5.54) | 1.43% (0.81-2.38) | 0.62 (0.55-0.69) |
| Malaysia | 140.02 (120.01-172.54) | 3.01 (2.46-3.64) | 533.47 (413.77-700.71) | 4.05 (3.12-5.31) | 2.81% (1.94-3.85) | 0.89 (0.57-1.22) |
| Maldives | 0.99 (0.76-1.38) | 3.56 (2.72-4.7) | 5.8 (4.52-7.15) | 3.89 (2.94-4.72) | 4.83% (3.01-7.2) | 0.05 (-0.04-0.15) |
| Mali | 67.77 (46.46-89.22) | 4.23 (2.84-5.53) | 178.63 (133.3-223.22) | 4.87 (3.56-6.07) | 1.64% (1.01-2.42) | 0.49 (0.41-0.58) |
| Malta | 14.84 (12.7-18.83) | 4.84 (4.11-6.42) | 30.56 (24.48-37.33) | 4.24 (3.38-5.21) | 1.06% (0.76-1.34) | -0.4 (-0.47--0.34) |
| Marshall Islands | 0.45 (0.32-0.6) | 5.7 (4.03-7.69) | 0.77 (0.58-1.02) | 6.15 (4.61-7.99) | 0.72% (0.32-1.31) | 0.32 (0.22-0.42) |
| Mauritania | 22.32 (16.72-27.47) | 4.54 (3.41-5.58) | 48.67 (36.46-60.69) | 4.61 (3.47-5.71) | 1.18% (0.63-1.85) | 0.02 (-0.02-0.07) |
| Mauritius | 11.97 (10.45-15.06) | 3.38 (2.85-4.24) | 34.41 (27.31-44.37) | 3.65 (2.88-4.76) | 1.87% (1.35-2.46) | 0.21 (0.09-0.33) |
| Mexico | 1202.05 (1033.62-1572.42) | 5.3 (4.48-7.09) | 3555.96 (2889.48-4749.03) | 5.23 (4.17-7.05) | 1.96% (1.57-2.35) | -0.19 (-0.34--0.05) |
| Micronesia (Federated States of) | 1.27 (0.93-1.72) | 5.94 (4.28-7.99) | 2.04 (1.53-2.7) | 7.08 (5.45-9.35) | 0.6% (0.16-1.17) | 0.59 (0.58-0.61) |
| Monaco | 3.19 (2.48-3.86) | 4.94 (3.87-5.97) | 3.85 (2.97-4.68) | 4.96 (3.87-5.96) | 0.21% (-0.07-0.55) | 0.13 (0.05-0.22) |
| Mongolia | 24.55 (19.23-29.16) | 4.6 (3.42-5.6) | 43.71 (34.93-53.53) | 4.99 (4.11-5.94) | 0.78% (0.39-1.32) | 0.15 (-0.03-0.33) |
| Montenegro | 43.92 (38.28-53.8) | 11.55 (9.83-13.83) | 99.28 (81.04-130.88) | 14.38 (11.31-19.38) | 1.26% (0.86-1.76) | 1.13 (0.96-1.3) |
| Morocco | 199.6 (138.81-249.97) | 3.07 (2.06-3.89) | 650.73 (506.24-789.6) | 4.37 (3.2-5.55) | 2.26% (1.6-3.1) | 1.25 (1.07-1.43) |
| Mozambique | 100.82 (52.16-140.48) | 3.83 (1.9-5.48) | 257.85 (151.16-372.32) | 5.33 (3.08-7.76) | 1.56% (0.85-2.59) | 1.34 (1.24-1.44) |
| Myanmar | 272.85 (210.41-349.36) | 2.69 (2.05-3.4) | 823.3 (674.79-1038.84) | 3.56 (2.88-4.4) | 2.02% (1.38-2.87) | 0.94 (0.88-1) |
| Namibia | 11.31 (8.62-13.97) | 3.28 (2.46-4.09) | 30.34 (22.55-38.64) | 4.1 (3.08-5.16) | 1.68% (1.05-2.48) | 0.61 (0.45-0.77) |
| Nauru | 0.08 (0.06-0.1) | 6.05 (4.87-7.54) | 0.06 (0.05-0.08) | 6.25 (4.9-7.96) | -0.18% (-0.35-0.06) | 0.13 (-0.03-0.29) |
| Nepal | 93.64 (55.04-146.16) | 2.31 (1.33-3.58) | 461.09 (306.66-615.32) | 4.08 (2.67-5.52) | 3.92% (2.36-5.91) | 1.99 (1.89-2.1) |
| Netherlands | 882.29 (746.21-1096.3) | 5.97 (4.91-7.28) | 1348.66 (1124.69-1800.35) | 5.27 (4.27-6.83) | 0.53% (0.41-0.75) | -0.63 (-0.73--0.52) |
| New Zealand | 189.01 (162.66-246.73) | 6.94 (5.84-8.89) | 410.59 (331.17-500.7) | 7.21 (5.71-8.71) | 1.17% (0.81-1.39) | -0.08 (-0.22-0.06) |
| Nicaragua | 30.81 (25.45-35.27) | 3.95 (3.18-4.55) | 144.03 (121.48-169.97) | 6.52 (5.45-7.64) | 3.67% (2.88-4.81) | 1.4 (0.82-1.98) |
| Niger | 35.35 (21.66-52.04) | 3.65 (2.19-5.53) | 115.5 (77.02-154.93) | 4.07 (2.7-5.48) | 2.27% (1.59-3.24) | 0.52 (0.45-0.59) |
| Nigeria | 988.78 (748.48-1385.52) | 5.03 (3.81-6.99) | 1768.03 (1339.84-2158.27) | 4.81 (3.58-5.91) | 0.79% (0.1-1.36) | -0.42 (-0.52--0.32) |
| Niue | 0.09 (0.07-0.11) | 5.13 (4.14-6.35) | 0.08 (0.06-0.1) | 5.05 (3.75-6.36) | -0.15% (-0.36-0.09) | -0.05 (-0.08--0.02) |
| North Macedonia | 71.35 (62.49-78.53) | 7.95 (6.58-8.93) | 140.97 (115.64-171.1) | 8.22 (6.78-9.93) | 0.98% (0.63-1.39) | 0.23 (0.13-0.33) |
| Northern Mariana Islands | 0.33 (0.27-0.42) | 6.24 (5.13-7.82) | 1.42 (1.19-1.63) | 6.73 (5.63-7.83) | 3.34% (2.48-4.32) | 0.57 (0.28-0.86) |
| Norway | 370.63 (292.41-434.51) | 6.27 (5.01-7.3) | 468.17 (318.06-534.48) | 6.76 (4.49-7.69) | 0.26% (0.04-0.4) | 0.18 (0.05-0.3) |
| Oman | 13.3 (8.31-17.79) | 7.45 (4.23-9.92) | 28.71 (22.88-34.67) | 8.38 (5.14-10.31) | 1.16% (0.53-2.53) | 0.43 (0.2-0.66) |
| Pakistan | 1073.51 (712.88-1362.66) | 3.34 (2.17-4.32) | 2153.63 (1669.34-2729.07) | 4.5 (3.41-5.79) | 1.01% (0.46-1.78) | 0.96 (0.81-1.12) |
| Palau | 0.17 (0.13-0.22) | 3.45 (2.56-4.55) | 0.29 (0.23-0.36) | 3.2 (2.53-3.87) | 0.73% (0.2-1.44) | -0.19 (-0.29--0.1) |
| Palestine | 18.31 (13.05-24.19) | 3.88 (2.78-5.07) | 47.54 (38.38-67.95) | 4.64 (3.74-6.6) | 1.6% (0.89-2.9) | 0.53 (0.4-0.67) |
| Panama | 29.15 (23.45-35.98) | 3.46 (2.67-4.29) | 100.23 (74.46-137.9) | 3.99 (2.93-5.39) | 2.44% (1.71-3.26) | 0.31 (0.21-0.41) |
| Papua New Guinea | 23.16 (14.25-37.63) | 3.69 (2.03-6.08) | 71.33 (47.49-104.55) | 4.01 (2.66-5.84) | 2.08% (1.4-2.96) | 0.34 (0.32-0.37) |
| Paraguay | 48.21 (40.21-55.56) | 4.19 (3.29-4.9) | 152.2 (115.86-198.65) | 4.69 (3.54-6.09) | 2.16% (1.39-3.14) | 0.3 (0.19-0.42) |
| Peru | 297.13 (237.54-352.16) | 4.45 (3.6-5.19) | 753.82 (561.09-1006.68) | 3.52 (2.65-4.62) | 1.54% (0.82-2.57) | -0.82 (-1--0.64) |
| Philippines | 295.88 (257.18-377.26) | 2.6 (2.24-3.25) | 1137.11 (938.98-1505.35) | 3.25 (2.68-4.25) | 2.84% (2.17-3.68) | 1.28 (1.05-1.51) |
| Poland | 1391.41 (1216.16-1798.27) | 4.62 (3.92-5.94) | 2863.05 (2153.16-3523.52) | 5.33 (4.05-6.47) | 1.06% (0.53-1.43) | 0.39 (0.32-0.47) |
| Portugal | 467.07 (407.42-655.39) | 5.03 (4.35-7.12) | 812.55 (667.79-1187.04) | 3.97 (3.23-5.84) | 0.74% (0.54-1.06) | -1.05 (-1.17--0.94) |
| Puerto Rico | 94.81 (76.3-115.57) | 4.12 (3.23-5.05) | 203.05 (147.15-261.8) | 3.88 (2.81-4.93) | 1.14% (0.7-1.66) | -0.25 (-0.33--0.17) |
| Qatar | 3.58 (2.35-4.59) | 12.02 (8.41-15.29) | 16.81 (12.28-22.17) | 12.12 (9.13-16.76) | 3.7% (2.16-6.27) | 0.06 (-0.26-0.37) |
| Republic of Korea | 373.85 (322.38-434.29) | 2.93 (2.47-3.42) | 1433.28 (1182.59-1636.09) | 2.57 (2.05-2.94) | 2.83% (2.13-3.6) | -0.45 (-0.5--0.39) |
| Republic of Moldova | 83.3 (69.46-97.94) | 3.81 (2.97-4.28) | 125.49 (103.47-165.1) | 3.41 (2.72-4.29) | 0.51% (0.28-0.85) | -0.83 (-1.11--0.54) |
| Romania | 587.51 (398.73-662.32) | 3.5 (2.36-3.98) | 1108.6 (699.21-1350.98) | 3.61 (2.31-4.34) | 0.89% (0.57-1.26) | -0.13 (-0.21--0.04) |
| Russian Federation | 4073.35 (3524.81-5871.23) | 3.71 (3.16-5.34) | 7045.78 (5841.62-9126.27) | 4.27 (3.5-5.65) | 0.73% (0.47-1) | 0.23 (0.04-0.41) |
| Rwanda | 69.32 (40.73-98.61) | 5.18 (2.97-7.46) | 134.71 (83.92-176.24) | 5.02 (3.08-6.6) | 0.94% (0.38-1.66) | -0.27 (-0.48--0.05) |
| Saint Kitts and Nevis | 2.77 (2.31-3.43) | 11.18 (9.46-13.88) | 3.01 (2.56-3.8) | 10.06 (8.48-12.53) | 0.09% (-0.08-0.27) | 0.23 (0.07-0.4) |
| Saint Lucia | 3.91 (3.29-4.93) | 8.08 (6.83-10.17) | 8.75 (7.06-11.07) | 7.55 (6.1-9.43) | 1.24% (0.87-1.61) | -0.78 (-1.07--0.49) |
| Saint Vincent and the Grenadines | 3.03 (2.34-3.45) | 6.64 (5.24-7.49) | 5.54 (4.58-7.17) | 7.02 (5.84-9.12) | 0.83% (0.5-1.34) | 0.49 (0.17-0.82) |
| Samoa | 2.44 (1.88-3.02) | 5.2 (3.97-6.46) | 4.1 (3.23-5.13) | 4.94 (3.87-6.22) | 0.68% (0.32-1.14) | -0.18 (-0.32--0.05) |
| San Marino | 1.2 (0.99-1.43) | 5.06 (4.11-6.04) | 2.8 (1.93-3.85) | 5.24 (3.71-6.99) | 1.33% (0.55-2.34) | 0.38 (0.27-0.49) |
| Sao Tome and Principe | 1.13 (0.83-1.36) | 3.73 (2.71-4.55) | 2.4 (1.87-2.93) | 5.03 (3.83-6.1) | 1.12% (0.55-1.94) | 1.29 (1.16-1.42) |
| Saudi Arabia | 98.44 (60.21-133.83) | 3.96 (2.39-5.41) | 194.22 (155.3-245.95) | 4.29 (3.35-5.29) | 0.97% (0.26-2.57) | -0.04 (-0.21-0.14) |
| Senegal | 53.99 (36.67-69.4) | 3.72 (2.5-4.73) | 158.56 (116.02-203.13) | 4.55 (3.35-5.8) | 1.94% (1.25-2.84) | 0.71 (0.66-0.77) |
| Serbia | 352.86 (306.43-476.84) | 5.1 (4.42-6.63) | 686.11 (552.64-964.09) | 6.28 (5.08-8.77) | 0.94% (0.55-1.39) | 0.64 (0.49-0.78) |
| Seychelles | 1.37 (1.11-1.65) | 3.75 (3-4.6) | 2.46 (1.77-3.21) | 4.17 (2.87-5.56) | 0.8% (0.45-1.28) | 0.35 (0.27-0.43) |
| Sierra Leone | 33.45 (24.08-44.88) | 3.39 (2.42-4.61) | 64.84 (50.16-85.73) | 3.98 (3.07-5.29) | 0.94% (0.47-1.6) | 0.73 (0.65-0.8) |
| Singapore | 28.74 (24.24-36.95) | 2.69 (2.2-3.25) | 80.82 (61.92-102.28) | 1.97 (1.44-2.37) | 1.81% (1.24-2.45) | -1.08 (-1.24--0.92) |
| Slovakia | 213.93 (189.34-234.58) | 4.98 (4.36-5.47) | 337.88 (270.96-415.19) | 5.17 (4.13-6.3) | 0.58% (0.27-0.95) | 0.22 (0.12-0.32) |
| Slovenia | 85.48 (66.75-113.05) | 4.75 (3.71-6.24) | 160.53 (107.71-208.75) | 4.37 (3.04-5.66) | 0.88% (0.27-1.59) | -0.19 (-0.34--0.04) |
| Solomon Islands | 1.96 (1.34-2.83) | 3.72 (2.54-5.32) | 5.63 (4.19-7.55) | 4.59 (3.47-6.03) | 1.87% (1.14-2.78) | 0.8 (0.74-0.85) |
| Somalia | 39.16 (19.23-59.01) | 3.88 (1.82-5.95) | 91.24 (43.66-134.52) | 3.7 (1.69-5.5) | 1.33% (0.71-2.26) | 0.03 (-0.1-0.16) |
| South Africa | 320.86 (265.16-371.04) | 3.08 (2.54-3.54) | 865.06 (750.38-961.06) | 3.96 (3.37-4.42) | 1.7% (1.34-2.06) | 0.81 (0.58-1.03) |
| South Sudan | 55.1 (30.43-78.35) | 4.34 (2.41-6.13) | 65.6 (38.33-95.99) | 3.75 (2.18-5.39) | 0.19% (-0.11-0.65) | -0.49 (-0.62--0.36) |
| Spain | 2316.02 (1962.64-2973.28) | 6.06 (5.06-7.85) | 4328.36 (3538.95-5471.2) | 5.41 (4.39-6.83) | 0.87% (0.67-1.09) | -0.46 (-0.53--0.39) |
| Sri Lanka | 136.38 (106.4-164.31) | 2.8 (2.19-3.38) | 450.24 (335.72-578.1) | 3.4 (2.53-4.3) | 2.3% (1.31-3.59) | 1.31 (1.08-1.54) |
| Sudan | 126.04 (86.89-165.94) | 2.66 (1.79-3.46) | 297.82 (225.14-382.36) | 3.19 (2.35-4.14) | 1.36% (0.75-2.2) | 0.62 (0.55-0.68) |
| Suriname | 6.89 (6.04-7.97) | 4.9 (4.1-5.6) | 17.8 (14.55-21.82) | 5.08 (4.06-6.16) | 1.58% (1.13-2.14) | 0.14 (-0.13-0.41) |
| Sweden | 749.02 (481.74-840.51) | 6.08 (3.75-6.84) | 1358.58 (643.91-1619.65) | 8.63 (4-10.32) | 0.81% (0.31-1.06) | 1.47 (1.34-1.59) |
| Switzerland | 299.08 (239.42-373.67) | 3.55 (2.82-4.39) | 466.17 (366.65-561.63) | 3.38 (2.66-4.05) | 0.56% (0.33-0.87) | -0.22 (-0.31--0.13) |
| Syrian Arab Republic | 93.78 (69.42-116.05) | 3.79 (2.85-4.67) | 251.53 (186.72-326.23) | 4.63 (3.35-5.95) | 1.68% (0.93-2.99) | 0.58 (0.5-0.66) |
| Taiwan (Province of China) | 212.88 (176.36-253.49) | 2.92 (2.37-3.44) | 838.42 (640.51-1136.04) | 3.32 (2.54-4.61) | 2.94% (2.1-4.1) | 0.4 (0.32-0.48) |
| Tajikistan | 71.66 (44.34-95.46) | 4.75 (2.46-6.88) | 112.17 (87.76-135.42) | 7.33 (5.59-8.78) | 0.57% (0.16-1.49) | 0.97 (0.47-1.46) |
| Thailand | 592.24 (482.04-728.63) | 4.11 (3.28-5.03) | 2054.53 (1515.77-2637.37) | 3.63 (2.66-4.62) | 2.47% (1.47-3.71) | -0.71 (-0.82--0.6) |
| Timor-Leste | 2.08 (1.52-2.88) | 2.14 (1.54-3) | 13.35 (10.55-16.97) | 3.49 (2.73-4.43) | 5.43% (3.88-7.96) | 1.93 (1.81-2.05) |
| Togo | 20.89 (16.05-25.92) | 4.21 (3.2-5.19) | 64.99 (50.03-83.77) | 4.89 (3.72-6.32) | 2.11% (1.37-3.19) | 0.56 (0.53-0.58) |
| Tokelau | 0.04 (0.03-0.06) | 4.64 (3.54-6.4) | 0.04 (0.03-0.05) | 4.87 (3.86-6) | -0.17% (-0.38-0.12) | 0.33 (0.27-0.38) |
| Tonga | 1.01 (0.76-1.23) | 3.73 (2.64-4.63) | 2.07 (1.59-2.6) | 4.14 (3.09-5.28) | 1.06% (0.55-1.7) | 0.31 (0.18-0.44) |
| Trinidad and Tobago | 20.57 (16.16-24.63) | 4.22 (3.42-5.12) | 49.31 (33.65-64.9) | 4.46 (3.16-5.73) | 1.4% (0.78-2.08) | 0.36 (0.21-0.51) |
| Tunisia | 81.38 (64.31-100.8) | 3.38 (2.68-4.15) | 283.66 (209.07-371.2) | 3.9 (2.83-5.07) | 2.49% (1.48-3.89) | 0.61 (0.5-0.71) |
| Turkey | 683.58 (520.9-837) | 3.78 (2.79-4.72) | 1558.57 (1208-2109.96) | 3.15 (2.43-3.97) | 1.28% (0.68-2.34) | -0.65 (-0.88--0.42) |
| Turkmenistan | 32.82 (27.69-42.78) | 3.31 (2.73-4.31) | 92.67 (73.28-123.49) | 4.65 (3.7-6.27) | 1.82% (1.2-2.47) | 0.88 (0.69-1.06) |
| Tuvalu | 0.17 (0.13-0.24) | 5.34 (3.89-7.34) | 0.3 (0.24-0.38) | 5.33 (4.22-6.75) | 0.73% (0.26-1.45) | -0.18 (-0.26--0.1) |
| Uganda | 117.55 (53.79-168.88) | 3.66 (1.62-5.21) | 304.29 (167.2-418.09) | 4.63 (2.49-6.47) | 1.59% (0.96-2.61) | 0.82 (0.8-0.85) |
| Ukraine | 2297.79 (1996.59-3073.09) | 4.99 (4.23-6.73) | 3278.31 (2727.22-4355.92) | 5.9 (4.79-7.92) | 0.43% (0.21-0.65) | 0.24 (0.11-0.37) |
| United Arab Emirates | 3.99 (2.45-6.18) | 4.23 (2.51-6.55) | 20.98 (11.31-37.98) | 4.12 (2.25-7.16) | 4.25% (2.23-6.76) | -0.29 (-0.89-0.31) |
| United Kingdom | 3835.89 (3094.61-4402.21) | 5.55 (4.31-6.2) | 5632.06 (4230.06-6348.7) | 5.86 (4.33-6.56) | 0.47% (0.31-0.56) | 0.16 (0.08-0.24) |
| United Republic of Tanzania | 259.96 (157.16-336.31) | 4.94 (2.91-6.48) | 659.27 (412.73-862.16) | 5.11 (3.21-6.7) | 1.54% (0.95-2.19) | 0.05 (-0.04-0.14) |
| United States of America | 10189.04 (8619.36-12743.37) | 4.01 (3.35-4.95) | 20729.34 (16844.12-25132.13) | 5.08 (4.12-6.16) | 1.03% (0.83-1.12) | 0.85 (0.77-0.93) |
| United States Virgin Islands | 2.51 (2.12-2.95) | 5.87 (4.96-6.84) | 8.12 (6.91-9.77) | 7.46 (6.24-8.97) | 2.23% (1.68-2.94) | 1.22 (1.04-1.4) |
| Uruguay | 122.48 (75.37-137.65) | 4.52 (2.78-5.12) | 202.49 (134.38-230.55) | 4.62 (3.13-5.27) | 0.65% (0.5-0.86) | 0.15 (0.03-0.27) |
| Uzbekistan | 177.47 (120.05-321.64) | 2.76 (1.81-5.14) | 447.02 (373.03-554.85) | 8.6 (6.78-10.05) | 1.52% (0.38-2.68) | 4.58 (4.23-4.94) |
| Vanuatu | 1.06 (0.67-1.59) | 3.55 (2.27-5.35) | 3.69 (2.74-4.93) | 4.32 (3.2-5.74) | 2.5% (1.61-3.95) | 0.74 (0.68-0.79) |
| Venezuela (Bolivarian Republic of) | 198.42 (163.47-243.6) | 3.94 (3.15-4.7) | 710.94 (533.46-944.36) | 4.39 (3.26-5.69) | 2.58% (1.8-3.49) | 0.04 (-0.11-0.19) |
| Viet Nam | 795.14 (654.96-983.74) | 3.76 (3.01-4.66) | 2179.23 (1667.16-2855.29) | 4.63 (3.53-5.96) | 1.74% (0.92-2.73) | 0.67 (0.56-0.77) |
| Yemen | 60.54 (38.05-84.73) | 2.91 (1.81-4.11) | 202 (160.49-263.2) | 3.34 (2.63-4.32) | 2.34% (1.57-3.85) | 0.66 (0.59-0.73) |
| Zambia | 54.93 (34.83-75.01) | 4.26 (2.63-5.86) | 200.29 (137.82-252.24) | 6.43 (4.49-8.1) | 2.65% (1.59-4.21) | 1.56 (1.34-1.79) |
| Zimbabwe | 53.17 (28.1-64.62) | 3.42 (1.6-4.14) | 99.16 (54.48-127.39) | 3.8 (1.94-4.86) | 0.86% (0.45-1.37) | 0.5 (0.36-0.63) |
