# Supplementary material for: Estimates of the global, regional, and national burden of atrial fibrillation in older adults from 1990 to 2019: insights from the Global Burden of Disease study 2019
Source: Front Public Health. 2023 Jun 12;11:1137230. doi: 10.3389/fpubh.2023.1137230 (PMC10291625; doi:10.3389/fpubh.2023.1137230)
Supplement: Supplementary Table 3 — The disability-adjusted life years (DALYs) of atrial fibrillation between 1990 and 2019 at national level. [file Table_3.DOCX]

**Supplementary Table 3. The disability-adjusted life years (DALYs) of atrial fibrillation between 1990 and 2019 at national level.**

| location | Cases in 1990 | ASDR in 1990 | Cases in 2019 | ASDR in 2019 | Percentage change | EAPC (95%) |
| --- | --- | --- | --- | --- | --- | --- |
| Afghanistan | 3182.49 (2348.07-4239.6) | 69.57 (51.66-91.18) | 4911.71 (3766.49-6336.66) | 76.41 (59.48-98.79) | 0.54% (0.34-0.76) | 0.37 (0.34-0.4) |
| Albania | 1654.67 (1258.84-2150.36) | 117.77 (90.7-149.63) | 4304.98 (3208.78-5715.19) | 120.14 (91.29-157.68) | 1.6% (1.32-1.91) | 0.14 (0.1-0.18) |
| Algeria | 6523.58 (5062.33-8262.87) | 92.14 (73.11-115.68) | 19725.55 (15485.5-24689.8) | 91.18 (71.78-111.62) | 2.02% (1.61-2.58) | 0.03 (0-0.06) |
| American Samoa | 16.06 (12.68-20.27) | 127.49 (103.53-157.99) | 39.42 (30.93-49.95) | 131.02 (104.81-162.47) | 1.45% (1.21-1.75) | 0.11 (0.09-0.13) |
| Andorra | 58.02 (42.34-77.63) | 142.87 (106.75-187.56) | 144.13 (105.55-191.29) | 133.72 (99.85-176.65) | 1.48% (1.05-2) | -0.32 (-0.39--0.26) |
| Angola | 1707.72 (1239.06-2210.43) | 84.07 (61.56-108.39) | 5716.15 (4364.98-7084.26) | 99.37 (74.9-123.73) | 2.35% (1.75-3.1) | 0.56 (0.55-0.58) |
| Antigua and Barbuda | 43.46 (36.4-51.97) | 95.99 (81.48-115.08) | 75.49 (63.53-91.57) | 114.01 (96.92-139.06) | 0.74% (0.51-0.97) | 0.47 (0.36-0.59) |
| Argentina | 22644.21 (18480.97-27942.82) | 92.61 (76.56-113.49) | 42344.11 (34627.11-53072.78) | 96.73 (79.44-120.36) | 0.87% (0.73-1.02) | 0.14 (0.09-0.2) |
| Armenia | 1920.62 (1423.83-2566.42) | 105.21 (79.34-138.6) | 4058.39 (3065.41-5389.25) | 122 (93.57-160.53) | 1.11% (0.92-1.35) | 0.52 (0.45-0.58) |
| Australia | 29297.61 (23069.8-37502.87) | 183.86 (146.41-231.11) | 58800.67 (46046.71-75911.93) | 167.01 (131.41-213.42) | 1.01% (0.9-1.15) | -0.4 (-0.42--0.37) |
| Austria | 14950.13 (11797.73-19229.83) | 143.07 (112.76-182.68) | 24857.25 (19309.45-31857.92) | 163.04 (126.86-207.93) | 0.66% (0.32-0.84) | 0.79 (0.54-1.03) |
| Azerbaijan | 3451.85 (2557.75-4616.18) | 106.79 (78.98-140.22) | 6910.14 (5262.03-8960.55) | 131.39 (103.7-165.3) | 1% (0.8-1.29) | 0.96 (0.86-1.06) |
| Bahamas | 98.14 (80.92-117.93) | 100 (83.33-120.14) | 251.16 (206.69-309.97) | 104.43 (86.3-129.47) | 1.56% (1.22-1.96) | 0.19 (0.08-0.31) |
| Bahrain | 88.12 (71.32-109.74) | 100.24 (82.24-126.38) | 661.77 (504.75-811.22) | 165.78 (129.43-198.77) | 6.51% (4.19-8.32) | 2.71 (2.27-3.14) |
| Bangladesh | 27815.99 (20477.54-36393.43) | 92.04 (68.78-118.86) | 95069.13 (70450.22-122236.55) | 105.11 (78.77-133.15) | 2.42% (1.87-3.06) | 0.55 (0.45-0.65) |
| Barbados | 216.74 (178.74-265.73) | 83.58 (69.51-101.03) | 353.88 (289.59-437.69) | 92.33 (76.17-113.36) | 0.63% (0.41-0.84) | 0.26 (0.21-0.32) |
| Belarus | 11573.92 (8516.09-15501.43) | 114.42 (84-151.57) | 17914.05 (13353.77-23746.77) | 138.54 (103.48-181.55) | 0.55% (0.36-0.75) | 0.64 (0.59-0.69) |
| Belgium | 17129.78 (13652.63-21820.77) | 129.91 (104.26-164.12) | 23262.34 (18891.81-29121.51) | 119.42 (96.46-149.63) | 0.36% (0.22-0.48) | -0.4 (-0.45--0.34) |
| Belize | 48.61 (38.38-61.8) | 71.7 (57.21-90.73) | 140.68 (115.17-174.54) | 83.32 (69.27-101.13) | 1.89% (1.36-2.34) | 0.41 (0.13-0.68) |
| Benin | 1015.82 (753.73-1287.63) | 75.12 (56.18-94.07) | 2404.46 (1908.27-2960.86) | 83.71 (67.66-102.29) | 1.37% (1.04-1.81) | 0.4 (0.33-0.46) |
| Bermuda | 43.64 (35.12-53.39) | 96.93 (78.94-116.98) | 80.06 (64.26-100.55) | 76.91 (62.35-95.72) | 0.83% (0.59-1.15) | -0.89 (-0.96--0.83) |
| Bhutan | 127.16 (91.88-170.93) | 91.26 (66.96-122.65) | 437.17 (324.2-563.83) | 111.59 (83.06-143.06) | 2.44% (1.82-3.24) | 0.73 (0.71-0.75) |
| Bolivia (Plurinational State of) | 1560.02 (1175.65-2166.9) | 77.6 (58.68-105.57) | 5448.87 (4136.74-7320.01) | 92.97 (70.89-121.81) | 2.49% (1.75-3.4) | 0.6 (0.58-0.62) |
| Bosnia and Herzegovina | 3537.88 (2679.53-4614.58) | 133.26 (103.75-169.82) | 7115.01 (5436.48-9181.88) | 145.72 (113.24-186.08) | 1.01% (0.8-1.32) | 0.36 (0.21-0.51) |
| Botswana | 240.04 (183.81-305.88) | 72.28 (56.44-89.83) | 589.07 (451.5-745.11) | 79.07 (60.67-100.19) | 1.45% (1.01-1.98) | 0.1 (-0.06-0.26) |
| Brazil | 55746.6 (45567.69-69648.72) | 98.67 (81.8-121.28) | 175983.73 (143622.87-216990.68) | 102.5 (84.33-124.48) | 2.16% (1.85-2.31) | 0.53 (0.4-0.65) |
| Brunei Darussalam | 67.67 (55.35-83.03) | 132.29 (110.09-158.33) | 184.17 (157.77-214.88) | 127.1 (110.49-146.41) | 1.72% (1.22-2.22) | -0.01 (-0.09-0.08) |
| Bulgaria | 13994.09 (10336.64-19102.44) | 145.4 (109.8-195.57) | 20021.55 (15347.8-26412.81) | 156.83 (120.82-204.6) | 0.43% (0.26-0.65) | 0.17 (0.11-0.24) |
| Burkina Faso | 2021.96 (1455.61-2637.89) | 76.77 (55.47-99.5) | 5185.15 (3992.04-6497) | 97.29 (75.09-121.7) | 1.56% (1.17-2.02) | 1.09 (0.96-1.21) |
| Burundi | 1219.19 (828.76-1634.65) | 81.77 (54.57-111.24) | 1639.15 (1142.74-2133.92) | 67.89 (46.25-88.39) | 0.34% (0.04-0.71) | -0.82 (-0.94--0.71) |
| Cabo Verde | 141.69 (111.89-178.41) | 70.79 (56.2-87.73) | 312.71 (253.78-376.09) | 100.8 (81.8-121.1) | 1.21% (0.85-1.77) | 0.88 (0.72-1.04) |
| Cambodia | 2188.87 (1577.96-2963.18) | 82.06 (60.63-107.52) | 7150.61 (5257.34-9476.51) | 95.56 (72.43-124.5) | 2.27% (1.93-2.63) | 0.52 (0.51-0.54) |
| Cameroon | 2147.64 (1590.12-2770.77) | 85.91 (64.61-109.4) | 6356.34 (5065.37-7859.31) | 97.63 (78.11-119.12) | 1.96% (1.42-2.72) | 0.49 (0.46-0.52) |
| Canada | 39179.64 (29607.88-52067.45) | 147.9 (112.36-193.6) | 82534.94 (61266.87-110734.99) | 142.69 (106.55-188.65) | 1.11% (0.99-1.22) | -0.26 (-0.3--0.21) |
| Central African Republic | 596.96 (411.26-788.59) | 98.19 (67.53-129.14) | 1028.26 (724.89-1351.16) | 97.78 (69.1-127.48) | 0.72% (0.42-1.13) | 0 (-0.04-0.05) |
| Chad | 1385.04 (983.26-1811.64) | 72.55 (51.25-94.33) | 2621.65 (1994.08-3299.9) | 79.93 (61.33-100.2) | 0.89% (0.62-1.23) | 0.39 (0.35-0.43) |
| Chile | 7632.16 (6248.99-9792.15) | 109.91 (91.28-136.9) | 21086.19 (17238.33-26366.73) | 112.55 (92.57-141.1) | 1.76% (1.58-1.93) | 0.17 (0.07-0.28) |
| China | 501239.52 (380036.21-652732.82) | 98.61 (76.85-124.84) | 1383673.61 (1047540.64-1802515.59) | 97.08 (75.21-123.12) | 1.76% (1.49-2.02) | -0.05 (-0.14-0.04) |
| Colombia | 7749.1 (6238.05-10128.02) | 69.85 (57.07-90.51) | 28464.23 (22406.01-36104.75) | 70.54 (55.84-88.37) | 2.67% (1.97-3.34) | -0.01 (-0.08-0.05) |
| Comoros | 111.1 (76.75-142.41) | 74.94 (51.37-95.54) | 238.62 (179.5-292.5) | 72.7 (54.24-89.34) | 1.15% (0.73-1.78) | -0.16 (-0.25--0.06) |
| Congo | 709.33 (519.44-902.6) | 113.78 (83.83-144.52) | 1602.08 (1265.17-1965.6) | 113.56 (90.29-139.8) | 1.26% (0.82-1.78) | -0.04 (-0.13-0.04) |
| Cook Islands | 10.34 (8-13.16) | 128.6 (101.85-159.51) | 22.9 (18.06-29.16) | 124.97 (99.65-157.03) | 1.22% (0.92-1.59) | -0.08 (-0.13--0.03) |
| Costa Rica | 845.73 (676.26-1040.06) | 67.87 (55.02-83.02) | 2731.75 (2114.02-3464.09) | 73.08 (57.4-92.39) | 2.23% (1.83-2.74) | 0.06 (-0.05-0.17) |
| Croatia | 5728.38 (4391.71-7537.32) | 116.02 (90.29-150.78) | 7955.47 (6027.87-10338.79) | 103.77 (78.57-135.05) | 0.39% (0.26-0.55) | -0.27 (-0.52--0.03) |
| Cuba | 5864.82 (4755.92-7233.12) | 73.17 (59.95-90.27) | 11147.63 (8806.56-14083.09) | 75.58 (60.87-94.11) | 0.9% (0.64-1.2) | 0.01 (-0.1-0.12) |
| Cyprus | 1106.8 (884.36-1366.71) | 177.96 (143.39-215.59) | 2384.92 (1947.8-2957.86) | 150.07 (124.21-182.95) | 1.15% (0.88-1.48) | -0.88 (-0.98--0.79) |
| Czechia | 15814.14 (12130.86-20904.89) | 137.03 (105.7-177.5) | 24257.3 (18347.75-32094.73) | 133.84 (102.33-174.44) | 0.53% (0.34-0.7) | 0.33 (0.19-0.48) |
| C么te d'Ivoire | 1494.43 (1121.26-1954.83) | 79.13 (60.37-99.23) | 4535.94 (3518.14-5665.57) | 83.68 (66.98-103.23) | 2.04% (1.58-2.58) | 0.21 (0.18-0.24) |
| Democratic People's Republic of Korea | 8743.85 (6661.75-11288.02) | 97.13 (74.76-122.91) | 22303.08 (17093.91-28639.25) | 96.57 (75.12-122.93) | 1.55% (1.29-1.89) | -0.05 (-0.11-0.01) |
| Democratic Republic of the Congo | 8735.26 (5578.87-14123.17) | 101.41 (64.49-165.61) | 20701.42 (14091.02-28607.8) | 104.42 (70.43-145.61) | 1.37% (0.85-2.01) | 0.06 (-0.03-0.15) |
| Denmark | 9772.89 (7818.91-12403.52) | 137.76 (110.85-172.83) | 13484.41 (10958.61-16761.23) | 137.23 (111.86-170.64) | 0.38% (0.26-0.5) | -0.13 (-0.25--0.01) |
| Djibouti | 48.51 (31.6-64.38) | 76.71 (48.63-101.31) | 225.29 (159.73-293.2) | 77.52 (53.58-100.61) | 3.64% (2.64-5.02) | 0.05 (-0.03-0.13) |
| Dominica | 68.81 (58.15-79.96) | 110.46 (93.45-127.95) | 84.65 (70.78-101.16) | 119.33 (100.15-142.62) | 0.23% (0.03-0.47) | 0.44 (0.36-0.51) |
| Dominican Republic | 1831.73 (1479.57-2224.91) | 76.12 (62.67-90.23) | 5901.04 (4727.89-7260.83) | 90.95 (73.12-111.08) | 2.22% (1.72-2.88) | 1.07 (0.92-1.22) |
| Ecuador | 1987.14 (1729.45-2371.08) | 55.06 (48.21-65.75) | 7161.85 (5787.48-8756.95) | 72.69 (59.44-88.27) | 2.6% (1.96-3.41) | 1.54 (1.26-1.82) |
| Egypt | 13737.99 (10324.62-17722.68) | 78.24 (59.11-98.86) | 31482.71 (22811.11-41787.64) | 85.85 (62.32-110.67) | 1.29% (0.96-1.71) | 0.38 (0.29-0.47) |
| El Salvador | 1357.57 (1102.78-1645.71) | 67.23 (55.21-79.86) | 3381.73 (2678.74-4204.92) | 73.52 (58.16-90.41) | 1.49% (1.11-1.93) | 0.21 (0.15-0.28) |
| Equatorial Guinea | 101.16 (68.96-135.13) | 87.52 (59.65-117.35) | 330.86 (236.67-451.04) | 119.46 (85.19-163.85) | 2.27% (1.24-3.6) | 1.25 (1.19-1.3) |
| Eritrea | 323.73 (211.61-472.21) | 74.14 (48.35-109.66) | 1073.38 (759.84-1379.62) | 85.11 (59.17-111.05) | 2.32% (1.47-3.46) | 0.32 (0.21-0.42) |
| Estonia | 1949.1 (1494.61-2567.47) | 118.26 (90.88-154.42) | 3171.46 (2381.5-4095.89) | 137.68 (103.3-179.54) | 0.63% (0.45-0.81) | 0.56 (0.5-0.63) |
| Eswatini | 113.89 (87.27-145.56) | 68.4 (53.91-86.51) | 248.08 (192.33-317.43) | 76.11 (59.09-96.47) | 1.18% (0.84-1.67) | 0.46 (0.28-0.63) |
| Ethiopia | 7300.95 (4698.61-9984.81) | 70.83 (44.27-97.18) | 17072.98 (11610.62-21424.61) | 67.64 (45.72-85.46) | 1.34% (0.76-2.2) | -0.25 (-0.39--0.12) |
| Fiji | 226.28 (175.23-291.07) | 120.75 (96.28-151.91) | 569.99 (446.23-722.21) | 133.13 (107.11-164.82) | 1.52% (1.15-1.95) | 0.4 (0.34-0.46) |
| Finland | 8038.36 (6236.22-10299.11) | 133.04 (104.08-167.91) | 12963.07 (9803.18-16816.54) | 120.88 (92.01-154.01) | 0.61% (0.5-0.76) | -0.35 (-0.36--0.33) |
| France | 100771.17 (79530.81-128642.95) | 138.94 (108.97-175.51) | 136017.45 (105482.56-174928.49) | 116.24 (90.34-148.61) | 0.35% (0.26-0.44) | -0.68 (-0.71--0.65) |
| Gabon | 415.59 (298.63-561.58) | 113.01 (79.53-152.71) | 763.92 (568.33-967.67) | 120.62 (90.4-152.56) | 0.84% (0.44-1.31) | 0.15 (0.11-0.2) |
| Gambia | 152.26 (113.44-198.31) | 75.2 (57.17-95.81) | 548.73 (437.97-673.75) | 89.47 (71.89-109.22) | 2.6% (2.05-3.4) | 0.6 (0.55-0.64) |
| Georgia | 5811.47 (4395.82-7640.42) | 124.35 (94.41-163.11) | 8062.19 (6296.02-10034.95) | 155.57 (122.96-193.37) | 0.39% (0.14-0.63) | 1.14 (0.9-1.38) |
| Germany | 173984 (132896.87-232510.86) | 158.51 (120.8-211.67) | 269377.2 (219823.88-336955.3) | 155.89 (126.49-196.4) | 0.55% (0.26-0.75) | 0.48 (0.3-0.66) |
| Ghana | 2372.44 (1815.23-3078.47) | 68.81 (54.3-86.62) | 7120.27 (5588.93-9004.17) | 76.96 (61.54-94.77) | 2% (1.61-2.46) | 0.38 (0.33-0.42) |
| Greece | 16897.05 (13034.56-22926.6) | 132.94 (103.37-178.5) | 25935.65 (19992.17-35200.52) | 119.96 (92.52-160.23) | 0.53% (0.43-0.71) | -0.51 (-0.56--0.46) |
| Greenland | 42.09 (33.98-51.31) | 223.01 (183.07-267.8) | 98.57 (79.15-122.99) | 214.86 (175.09-264.43) | 1.34% (1.04-1.66) | -0.26 (-0.35--0.17) |
| Grenada | 59.52 (48.61-72.25) | 93.5 (77.16-111.95) | 71.32 (59.93-88.25) | 98.21 (83.59-121.68) | 0.2% (0.01-0.39) | 0.1 (-0.01-0.21) |
| Guam | 54.71 (43.43-68.34) | 132.78 (109.68-161.95) | 138.06 (105.12-183.06) | 105.84 (83.07-136.07) | 1.52% (1.19-1.84) | -0.83 (-1.04--0.61) |
| Guatemala | 1467.65 (1160.83-1796.9) | 72.2 (57.32-87.04) | 5117.35 (4002.69-6476.51) | 67.39 (53.14-83.25) | 2.49% (1.97-3.07) | -0.59 (-0.69--0.49) |
| Guinea | 1663.62 (1221.65-2130.27) | 75.77 (55.8-96.19) | 2885.82 (2232.25-3580.82) | 82.98 (65.41-102.29) | 0.73% (0.46-1.07) | 0.39 (0.34-0.45) |
| Guinea-Bissau | 185.03 (136.28-237.41) | 79.83 (60.07-101.31) | 328.86 (255.87-411.63) | 87.98 (69.13-109.87) | 0.78% (0.45-1.19) | 0.4 (0.37-0.43) |
| Guyana | 238.86 (199.83-304.43) | 100.58 (85.93-125.68) | 393 (315.07-496.03) | 108.96 (88.07-136.65) | 0.65% (0.38-0.93) | 0.2 (0.09-0.32) |
| Haiti | 1934.03 (1462.09-2557.29) | 107.04 (79.8-140.71) | 3914.09 (2945.79-5400.68) | 98.37 (74.55-135.09) | 1.02% (0.63-1.57) | -0.23 (-0.29--0.17) |
| Honduras | 907.35 (665.29-1288.43) | 69.5 (49.9-101.76) | 3613.35 (2901.58-4548.05) | 92.63 (74.86-116.14) | 2.98% (2.2-3.8) | 1.19 (1.02-1.35) |
| Hungary | 17544.51 (13388.24-22976.17) | 144.59 (110.96-186.34) | 22171.58 (16723.92-29227.39) | 131.8 (100.04-172.95) | 0.26% (0.17-0.37) | -0.37 (-0.4--0.34) |
| Iceland | 357.62 (288.38-454.24) | 147.76 (119.09-187.48) | 601.92 (477.58-772.15) | 133.41 (105.29-168.57) | 0.68% (0.55-0.8) | -0.1 (-0.2-0) |
| India | 241951.95 (178646.51-319564.36) | 97.36 (74.6-125.5) | 821092.07 (625390.4-1046877.75) | 105.66 (81.67-132.84) | 2.39% (1.98-2.84) | 0.22 (0.17-0.27) |
| Indonesia | 54277.52 (39986.79-72724.39) | 96.33 (72.41-126.77) | 143817.35 (107954.75-188957.73) | 113.21 (86.59-143.72) | 1.65% (1.44-1.93) | 0.55 (0.53-0.57) |
| Iran (Islamic Republic of) | 10305.87 (7790.73-13490.22) | 75.31 (57.33-95.61) | 36790.75 (28810.77-46710.77) | 75.3 (59.93-94.58) | 2.57% (2.23-3.18) | -0.06 (-0.23-0.1) |
| Iraq | 3948.4 (2882.45-5666.34) | 83.87 (63.61-116.26) | 11900.15 (8924.62-17671.44) | 96.62 (75.38-137.25) | 2.01% (1.59-2.57) | 0.5 (0.44-0.57) |
| Ireland | 5353.53 (4323.58-6780.57) | 152.66 (123.78-192.91) | 8455.54 (6602.26-10706.09) | 137.38 (107.66-171.91) | 0.58% (0.43-0.7) | -0.61 (-0.74--0.48) |
| Israel | 5630.09 (4524.89-7124.95) | 137.75 (111.29-173.54) | 11062.38 (8535.92-14315.32) | 116.32 (90.74-150.52) | 0.96% (0.84-1.14) | -0.86 (-0.95--0.76) |
| Italy | 105386.95 (82397.36-137855.29) | 140.1 (110.3-182.78) | 146752.68 (115983.92-185838.67) | 115.84 (91.37-147.56) | 0.39% (0.24-0.51) | -0.78 (-0.84--0.72) |
| Jamaica | 1095.65 (888.07-1370.95) | 78.18 (64.67-94.84) | 1980.45 (1540.2-2653.08) | 93.1 (74.03-120.71) | 0.81% (0.5-1.15) | 0.51 (0.37-0.66) |
| Japan | 82554.05 (67719.69-103441.69) | 66.28 (54.13-82.97) | 155475.51 (123614.04-193404.04) | 53.15 (42.54-66.74) | 0.88% (0.74-1.07) | -1.15 (-1.32--0.97) |
| Jordan | 611.65 (477.85-778.36) | 88.73 (70.68-110.64) | 2943.68 (2285.07-3716.05) | 85.19 (68.51-104.14) | 3.81% (3.18-4.55) | -0.23 (-0.32--0.15) |
| Kazakhstan | 10353.76 (7373.6-14529.9) | 119.72 (86.33-165.61) | 16350.33 (12016.37-23078.82) | 146.76 (110.39-207.2) | 0.58% (0.38-0.78) | 0.48 (0.37-0.59) |
| Kenya | 3138.55 (2267.79-4048.82) | 61.52 (43.95-79.26) | 9387.54 (6788.82-11977.18) | 78.47 (56.47-99.88) | 1.99% (1.54-2.73) | 1.28 (1.1-1.46) |
| Kiribati | 24.6 (19.04-31.6) | 125.08 (98.8-157.28) | 41.66 (32.35-53.38) | 124.71 (98.8-154.5) | 0.69% (0.47-0.95) | -0.05 (-0.09--0.01) |
| Kuwait | 199.9 (150.12-259.55) | 66.55 (50.97-85.8) | 860.3 (643.42-1143.18) | 64.7 (49.47-84.46) | 3.3% (2.94-3.77) | 0.07 (-0.07-0.22) |
| Kyrgyzstan | 2191.93 (1640.17-2907.46) | 97.02 (72.81-127.74) | 3089.19 (2319.48-4063.28) | 108.95 (82.81-140.7) | 0.41% (0.32-0.51) | 0.41 (0.21-0.61) |
| Lao People's Democratic Republic | 1113.14 (787.46-1534.77) | 88.84 (65.14-117.9) | 2574 (1937.86-3377.61) | 100.43 (77.33-127.89) | 1.31% (1.06-1.59) | 0.37 (0.35-0.39) |
| Latvia | 3232.64 (2472.33-4257.01) | 112.9 (86.81-148.08) | 4386.2 (3327.78-5739.82) | 126.88 (95.98-164.14) | 0.36% (0.25-0.47) | 0.49 (0.39-0.58) |
| Lebanon | 1281.64 (1016.57-1632.69) | 87.09 (69.39-109.06) | 3576.97 (2695.84-4551.95) | 86.47 (65.25-108.85) | 1.79% (1.27-2.4) | -0.07 (-0.08--0.05) |
| Lesotho | 395.91 (297.17-518.44) | 62.81 (47.86-80.52) | 581.73 (443.24-730.86) | 79.68 (61.39-99.6) | 0.47% (0.24-0.8) | 1.1 (0.99-1.21) |
| Liberia | 568.15 (435.68-734.39) | 77.59 (59.94-98.67) | 909.88 (708.28-1162) | 80.71 (63.04-101.6) | 0.6% (0.37-0.9) | 0.26 (0.18-0.33) |
| Libya | 791.94 (584.57-1062.63) | 66.48 (49.76-88.61) | 2224.52 (1607.86-2915.03) | 70.76 (51.83-91.33) | 1.81% (1.42-2.35) | 0.29 (0.26-0.33) |
| Lithuania | 4301.22 (3254.05-5640.62) | 120.14 (92.36-155.08) | 6612.9 (4974.42-8621.49) | 133.1 (99.48-174.78) | 0.54% (0.41-0.68) | 0.35 (0.28-0.43) |
| Luxembourg | 708.31 (564.55-904.7) | 152.79 (122.52-194.4) | 1144.18 (895.14-1448.08) | 140.2 (109.56-179.75) | 0.62% (0.43-0.77) | -0.16 (-0.32--0.01) |
| Madagascar | 2773.1 (1877.52-3497.23) | 89.87 (60.46-112.95) | 5037.59 (3572.64-6414.06) | 93.69 (65.65-120.29) | 0.82% (0.42-1.31) | -0.03 (-0.11-0.05) |
| Malawi | 1380.57 (928.1-1781.83) | 63.56 (41.82-81.89) | 2988.98 (1998.74-3788.76) | 70.07 (46.97-87.9) | 1.17% (0.73-1.75) | 0.38 (0.34-0.43) |
| Malaysia | 5785.18 (4377.48-7681.35) | 98.65 (76.21-127.75) | 19301.13 (14601.39-25298.37) | 109.7 (84.5-139.98) | 2.34% (2.01-2.75) | 0.38 (0.27-0.5) |
| Maldives | 44.38 (31.82-59.8) | 97.1 (72.6-126.66) | 181 (136.46-235.34) | 99.21 (75.87-127.04) | 3.08% (2.44-3.78) | -0.05 (-0.09--0.01) |
| Mali | 1828.03 (1330.21-2361.53) | 77.51 (57.29-98.72) | 4203.67 (3260.13-5281.44) | 84.82 (66.2-105.27) | 1.3% (0.97-1.67) | 0.32 (0.28-0.36) |
| Malta | 455.97 (358.44-596.72) | 130.35 (102.67-168.94) | 934.98 (729.18-1206.76) | 114.9 (89.45-147.87) | 1.05% (0.88-1.2) | -0.33 (-0.36--0.29) |
| Marshall Islands | 13.25 (10.18-17.02) | 136.72 (106.55-172.72) | 24.28 (18.73-31.08) | 147.09 (114.96-187.12) | 0.83% (0.54-1.18) | 0.28 (0.21-0.34) |
| Mauritania | 547.86 (430.23-684) | 83.92 (66.62-102.29) | 1125.06 (879.4-1393.47) | 83.45 (66.01-103.11) | 1.05% (0.73-1.41) | 0 (-0.03-0.03) |
| Mauritius | 482.84 (367.72-635.97) | 101.37 (79.56-131.85) | 1269.02 (959.81-1667.94) | 103.71 (80.14-133) | 1.63% (1.4-1.88) | 0.01 (-0.05-0.08) |
| Mexico | 24826.75 (20986.54-30520.91) | 90.27 (76.84-110.44) | 74264.49 (60926.8-92280.94) | 91.86 (75.82-114.22) | 1.99% (1.71-2.27) | -0.03 (-0.1-0.04) |
| Micronesia (Federated States of) | 38.39 (29.34-49.35) | 140.53 (108.57-179.01) | 55.87 (43.29-70.53) | 156.79 (122.38-198.07) | 0.46% (0.17-0.77) | 0.37 (0.36-0.38) |
| Monaco | 86.76 (67.27-110.38) | 132.15 (102.9-168.33) | 100.92 (77.83-128.48) | 122.34 (95.13-155.06) | 0.16% (0.02-0.32) | -0.27 (-0.29--0.26) |
| Mongolia | 865.14 (664.49-1114.04) | 122.55 (95.05-157.13) | 1590.41 (1198.99-2086.01) | 127.39 (98.98-161.65) | 0.84% (0.64-1.08) | 0.01 (-0.08-0.11) |
| Montenegro | 1003.12 (839.01-1227.11) | 223.28 (188.18-270.88) | 2011.52 (1652.69-2515.39) | 250.62 (205.82-313.72) | 1.01% (0.75-1.33) | 0.62 (0.53-0.71) |
| Morocco | 6576.12 (5058.07-8626.21) | 76.25 (59.27-96.92) | 17387.43 (13706.15-21961.48) | 89.32 (70.27-110.18) | 1.64% (1.33-1.99) | 0.56 (0.48-0.64) |
| Mozambique | 2213.17 (1372.95-2921.45) | 65.73 (40.22-86.87) | 5066.21 (3297.11-6871.17) | 84.39 (54.17-116.32) | 1.29% (0.8-1.96) | 1.03 (0.95-1.11) |
| Myanmar | 12925.15 (9426.59-17170.61) | 90.84 (68.29-117.48) | 29561.27 (22283.38-38520.38) | 99.7 (76.99-126.11) | 1.29% (1.07-1.58) | 0.32 (0.29-0.35) |
| Namibia | 336.4 (262.03-424.73) | 71.69 (55.95-88.7) | 727.37 (559.54-912.63) | 80.95 (63.56-100.37) | 1.16% (0.83-1.52) | 0.33 (0.24-0.43) |
| Nauru | 2.27 (1.79-2.88) | 146.05 (117.46-183.03) | 2.12 (1.61-2.74) | 150.32 (117.66-190.06) | -0.07% (-0.19-0.1) | 0.08 (-0.04-0.21) |
| Nepal | 4566.04 (3220.97-6394.79) | 81.83 (58.35-112.01) | 15100.36 (11320.5-19742.49) | 100.29 (75.42-129.72) | 2.31% (1.84-2.94) | 0.71 (0.67-0.75) |
| Netherlands | 21823.61 (17888.71-26864.89) | 129.44 (106.15-158.6) | 34853.58 (27391.16-44686.59) | 120.3 (95.51-153.9) | 0.6% (0.46-0.75) | -0.43 (-0.53--0.34) |
| New Zealand | 5924.83 (4584.84-7698.45) | 183.26 (143.76-237.07) | 11365.58 (9055.65-14346.65) | 175.07 (139.28-218.7) | 0.92% (0.74-1.09) | -0.2 (-0.23--0.17) |
| Nicaragua | 710.79 (586.62-854.13) | 73.65 (61.7-87.35) | 2784.3 (2339.27-3304.93) | 99.89 (84.02-117.79) | 2.92% (2.43-3.53) | 0.85 (0.48-1.21) |
| Niger | 1065.43 (753.01-1420.93) | 72.69 (51.73-97.42) | 3206.71 (2336.22-4153.96) | 77.14 (56.98-98.57) | 2.01% (1.65-2.47) | 0.27 (0.23-0.31) |
| Nigeria | 23738.48 (18302.38-30622.84) | 89.28 (69.61-114.86) | 42546.61 (33261.21-52787.34) | 87.69 (68.73-107.82) | 0.79% (0.34-1.11) | -0.21 (-0.27--0.15) |
| Niue | 2.26 (1.78-2.81) | 128.64 (102.77-158.94) | 2.1 (1.64-2.68) | 130.4 (103.33-164.13) | -0.07% (-0.2-0.06) | 0.02 (-0.01-0.05) |
| North Macedonia | 1983.46 (1611.3-2473.05) | 160.46 (132.03-195.9) | 3774.84 (2980.45-4776.67) | 159.84 (129.27-197.35) | 0.9% (0.71-1.14) | 0 (-0.03-0.03) |
| Northern Mariana Islands | 9.95 (7.93-12.54) | 145.29 (117.83-179.81) | 42.07 (34.08-51.69) | 153.03 (125.88-182.92) | 3.23% (2.67-3.76) | 0.37 (0.2-0.54) |
| Norway | 8998.41 (7324.07-11207.15) | 145.51 (118.14-180.62) | 10860.99 (8394.63-13543.67) | 138.06 (108.02-170.33) | 0.21% (0.09-0.28) | -0.3 (-0.37--0.23) |
| Oman | 326.38 (240.81-421.34) | 112.66 (80.36-142.8) | 737.18 (593.44-926.43) | 120.67 (95.12-145.85) | 1.26% (0.8-1.95) | 0.29 (0.16-0.42) |
| Pakistan | 39378.04 (29304.99-52220.74) | 99.63 (74.85-130.29) | 72301.87 (55032.08-92255.62) | 112.61 (87.07-141.19) | 0.84% (0.61-1.11) | 0.4 (0.33-0.46) |
| Palau | 6.62 (4.95-8.73) | 106.25 (80.98-136.69) | 12.51 (9.45-16.47) | 104.56 (81.28-132.36) | 0.89% (0.61-1.19) | -0.04 (-0.07--0.01) |
| Palestine | 484.97 (369.94-626.5) | 85.31 (65.53-108.51) | 1213.84 (955.17-1574.4) | 92.92 (74.6-117.47) | 1.5% (1.1-2.2) | 0.26 (0.19-0.33) |
| Panama | 699.17 (572.25-867.75) | 67.54 (55.84-81.86) | 2215.56 (1734.19-2827.86) | 73.5 (57.6-93.12) | 2.17% (1.75-2.66) | 0.22 (0.17-0.27) |
| Papua New Guinea | 976.79 (694.12-1320.5) | 101.68 (73.06-137.63) | 2597.71 (1928.84-3422.54) | 111.18 (83.7-145.8) | 1.66% (1.35-2) | 0.33 (0.31-0.35) |
| Paraguay | 1271.58 (1022.41-1595.59) | 86.49 (70.09-105.91) | 3626.03 (2854.46-4588.44) | 95.45 (75.89-119.73) | 1.85% (1.42-2.4) | 0.3 (0.23-0.36) |
| Peru | 5041.61 (4202-5998.91) | 65.03 (54.71-76.15) | 12957.95 (10140.03-16391.46) | 54.39 (42.48-68.2) | 1.57% (1-2.35) | -0.58 (-0.7--0.45) |
| Philippines | 15259.61 (10893.8-20953.88) | 85.55 (63.21-115.27) | 46186.98 (34362.38-61002.34) | 97.53 (74.38-126.25) | 2.03% (1.82-2.34) | 0.59 (0.49-0.7) |
| Poland | 48156.01 (37159.26-62844.8) | 138.33 (107.6-179.68) | 86928.63 (67443.8-111580.37) | 146.05 (113.54-186.37) | 0.81% (0.58-0.97) | 0.34 (0.16-0.52) |
| Portugal | 15486.02 (11920.38-20396.41) | 134.59 (104.83-173.58) | 23471.66 (17873.49-31015.72) | 110.87 (84.5-145.72) | 0.52% (0.42-0.65) | -0.84 (-0.91--0.77) |
| Puerto Rico | 2085.35 (1722.84-2550.2) | 74.69 (62.18-90.14) | 4365.96 (3384.67-5511.91) | 73.03 (57.64-92.08) | 1.09% (0.82-1.42) | -0.12 (-0.18--0.07) |
| Qatar | 76.07 (52.67-93.93) | 194.99 (142.12-238.95) | 436.19 (329.21-557.18) | 178.86 (142.32-228.96) | 4.73% (3.16-7.06) | -0.28 (-0.47--0.1) |
| Republic of Korea | 10993.79 (8788.88-13692.51) | 65.94 (53.47-80.34) | 36853.3 (29477.39-46145.21) | 56.82 (45.46-70.8) | 2.35% (1.96-2.73) | -0.53 (-0.57--0.49) |
| Republic of Moldova | 3713.17 (2764.57-5029.16) | 119.7 (91.07-156.81) | 5444.86 (4006.33-7322.28) | 120.03 (89.33-159.45) | 0.47% (0.37-0.58) | -0.2 (-0.29--0.1) |
| Romania | 25517.23 (19092.37-33932.05) | 118.37 (89.69-153.47) | 38332.55 (28633.12-50567.68) | 116.9 (87.37-153.71) | 0.5% (0.38-0.65) | -0.45 (-0.57--0.32) |
| Russian Federation | 157194.01 (117333.38-209440.37) | 115.6 (87.61-151.51) | 248861.73 (187099.33-327907.73) | 130.9 (99.5-170.9) | 0.58% (0.49-0.68) | 0.37 (0.28-0.46) |
| Rwanda | 1445.48 (949.73-1970.04) | 87.11 (57.99-119.21) | 2647.95 (1857.27-3314.68) | 79.35 (54.68-99.08) | 0.83% (0.39-1.34) | -0.54 (-0.74--0.34) |
| Saint Kitts and Nevis | 48.83 (41.04-58.42) | 161.84 (138.29-194.4) | 56.19 (47.7-69.16) | 146.73 (125.35-182.39) | 0.15% (0-0.32) | -0.07 (-0.2-0.05) |
| Saint Lucia | 72.29 (60.73-87.96) | 117.82 (100.79-140.18) | 159.64 (132.49-194.31) | 110.01 (91.89-133.88) | 1.21% (0.93-1.48) | -0.62 (-0.83--0.4) |
| Saint Vincent and the Grenadines | 56.75 (45.87-66.61) | 104.09 (85.51-120.95) | 102.65 (85.4-127.03) | 108.12 (91.09-133.85) | 0.81% (0.55-1.16) | 0.3 (0.07-0.53) |
| Samoa | 72.64 (56.22-91.87) | 130.54 (102.3-161.39) | 118.54 (92.7-150.92) | 129.58 (101.89-162.28) | 0.63% (0.44-0.86) | -0.03 (-0.08-0.02) |
| San Marino | 36.57 (27.89-47.02) | 130.91 (101.09-168.61) | 68.54 (50.71-90.62) | 123.64 (91.42-160.96) | 0.87% (0.55-1.28) | -0.12 (-0.16--0.08) |
| Sao Tome and Principe | 31.99 (24.42-40.86) | 74.33 (57.42-92.05) | 55.5 (43.97-69.21) | 90.16 (72.25-111.18) | 0.73% (0.47-1.07) | 0.76 (0.73-0.8) |
| Saudi Arabia | 2633.54 (1915.65-3432.51) | 81.78 (59.92-105.68) | 5882.61 (4522.4-7553.96) | 85.11 (66.98-105.14) | 1.23% (0.75-2.02) | -0.02 (-0.11-0.08) |
| Senegal | 1494.08 (1117.15-1936.59) | 74.54 (55.64-93.7) | 3796.14 (2974.55-4798.82) | 82.46 (64.54-103.12) | 1.54% (1.21-1.97) | 0.38 (0.34-0.41) |
| Serbia | 10832.16 (8393.93-14246.05) | 131.11 (103.89-169.12) | 18865.92 (14501.4-24433.56) | 139.63 (108.59-179.45) | 0.74% (0.55-0.96) | 0.05 (-0.03-0.13) |
| Seychelles | 46.25 (35.32-59.93) | 105.83 (82.07-134.34) | 78.45 (58.57-102.33) | 108.85 (82.2-139.96) | 0.7% (0.55-0.89) | 0.06 (0.02-0.1) |
| Sierra Leone | 976.04 (727.81-1280.92) | 73.94 (56.17-95.1) | 1724.68 (1304.64-2185.07) | 79.39 (61.49-100.19) | 0.77% (0.52-1.07) | 0.32 (0.28-0.36) |
| Singapore | 861.62 (686.98-1101.93) | 64.24 (51.78-80.19) | 2720.81 (2068.12-3568.93) | 51.94 (40.06-67.04) | 2.16% (1.77-2.54) | -0.78 (-0.84--0.72) |
| Slovakia | 7405.2 (5734.46-9548.89) | 152.24 (118.25-193.29) | 11177.76 (8554.1-14541.43) | 146.25 (112.91-187.67) | 0.51% (0.35-0.68) | 0.1 (-0.08-0.29) |
| Slovenia | 2644.69 (2035.13-3434.56) | 134.37 (104.22-173) | 4679.38 (3526.64-6134.57) | 124.96 (94.67-162.2) | 0.77% (0.48-1.08) | -0.25 (-0.33--0.18) |
| Solomon Islands | 78.09 (56.38-103.87) | 115.5 (84.48-152.24) | 189.55 (143.24-242.54) | 127.94 (98.35-163.44) | 1.43% (1.07-1.83) | 0.38 (0.35-0.42) |
| Somalia | 846.49 (505.26-1170.94) | 69.57 (40.6-96.64) | 2124.59 (1275.27-2908.65) | 64.46 (37.68-89.04) | 1.51% (0.99-2.23) | -0.15 (-0.25--0.06) |
| South Africa | 9613.16 (7565.95-12345.28) | 72.78 (58.79-90.96) | 22836.97 (18564.38-28537.62) | 80.28 (66.26-97.2) | 1.38% (1.21-1.54) | 0.33 (0.2-0.47) |
| South Sudan | 1107.15 (721.62-1484.55) | 73.82 (46.69-98.45) | 1369.43 (931.9-1853.71) | 64.93 (43.6-87.68) | 0.24% (-0.02-0.58) | -0.44 (-0.55--0.34) |
| Spain | 66529.57 (52602.78-85581.24) | 145.16 (115.33-185.04) | 102680.63 (81304.41-129954.95) | 123.88 (97.64-157.71) | 0.54% (0.44-0.67) | -0.6 (-0.71--0.48) |
| Sri Lanka | 5980.49 (4359.1-8045.18) | 87.6 (65.84-113.82) | 17207.71 (12899.09-22901.67) | 94.03 (71.76-122.08) | 1.88% (1.53-2.28) | 0.46 (0.38-0.54) |
| Sudan | 4295.73 (3260.54-5778.57) | 70.88 (54.38-92.57) | 8842.58 (6724.51-11504.44) | 77.82 (59.82-99.82) | 1.06% (0.77-1.39) | 0.32 (0.28-0.35) |
| Suriname | 143.7 (120.88-168.85) | 85.15 (72.82-99.11) | 366.62 (301.86-444.3) | 89.87 (73.93-107.84) | 1.55% (1.23-1.92) | 0.18 (0.03-0.34) |
| Sweden | 22785.59 (17496.3-29630.94) | 168.71 (129.82-217.59) | 33709.43 (23645.89-43524.23) | 188.07 (132.83-241.02) | 0.48% (0.27-0.62) | 0.67 (0.48-0.85) |
| Switzerland | 9159.5 (7116.57-11838.21) | 102.09 (79.11-131.09) | 13507.38 (10350.18-17463.6) | 92.69 (71.5-120.19) | 0.47% (0.36-0.61) | -0.18 (-0.37-0.02) |
| Syrian Arab Republic | 2599.4 (2010.54-3292.03) | 83.34 (65.11-103.79) | 6711.94 (5155.88-8525.57) | 90.57 (69.49-113.06) | 1.58% (1.18-2.21) | 0.2 (0.15-0.26) |
| Taiwan (Province of China) | 9065.14 (6813.3-12131.44) | 87.52 (67.54-112.49) | 26541.2 (19938.36-34415.37) | 87.09 (66.54-111.88) | 1.93% (1.6-2.37) | -0.07 (-0.12--0.03) |
| Tajikistan | 2251.6 (1646.55-2969.07) | 115.86 (83.16-152.2) | 3470.22 (2688.39-4439.32) | 147.07 (117.62-182.69) | 0.54% (0.34-0.85) | 0.7 (0.53-0.86) |
| Thailand | 20589.14 (15620.74-26636.22) | 98.48 (76.76-124.7) | 71245.1 (52889.2-95014.35) | 95.56 (72.27-124.94) | 2.46% (1.98-2.92) | -0.24 (-0.29--0.19) |
| Timor-Leste | 111.08 (78.95-154.39) | 80.75 (58.71-109.99) | 529.4 (388.23-710.09) | 96.73 (72.81-126.94) | 3.77% (3.28-4.43) | 0.74 (0.67-0.8) |
| Togo | 554.7 (428.12-696.74) | 80.21 (62.79-99.53) | 1628.86 (1262.08-2029.38) | 86.61 (68.31-106.93) | 1.94% (1.5-2.51) | 0.28 (0.27-0.29) |
| Tokelau | 1.32 (1-1.68) | 124.41 (95.37-160.06) | 1.14 (0.89-1.45) | 127.86 (101.81-159.67) | -0.14% (-0.27-0.01) | 0.16 (0.14-0.19) |
| Tonga | 37.93 (28.57-49.07) | 108.46 (84.47-138.38) | 65.57 (50.92-84.33) | 117.17 (91.79-150.11) | 0.73% (0.52-0.98) | 0.24 (0.17-0.31) |
| Trinidad and Tobago | 470.08 (377.29-568.95) | 78.11 (63.87-94.03) | 1113.23 (842.66-1417.41) | 82.78 (63.32-104) | 1.37% (0.96-1.81) | 0.27 (0.19-0.35) |
| Tunisia | 2501.91 (1948.99-3231.24) | 76.74 (61.13-97.05) | 7375.9 (5642.63-9449.01) | 82.77 (63.74-104.79) | 1.95% (1.5-2.53) | 0.32 (0.27-0.38) |
| Turkey | 19526.18 (15377.38-24652.95) | 85.52 (67.83-106.82) | 47024.85 (35421.9-61026.11) | 74.82 (57.84-95.3) | 1.41% (1.05-1.88) | -0.7 (-0.78--0.61) |
| Turkmenistan | 1351.67 (1013.86-1783.55) | 107.08 (81.44-140.39) | 3161.14 (2406.51-4104.26) | 130.65 (100.58-167.74) | 1.34% (1.09-1.62) | 0.56 (0.46-0.66) |
| Tuvalu | 5.57 (4.26-7.29) | 134.54 (103.49-174.24) | 8.92 (6.95-11.35) | 135.04 (106.88-169.56) | 0.6% (0.33-0.92) | -0.07 (-0.1--0.03) |
| Uganda | 2543.1 (1484.53-3370.09) | 64.55 (37.58-86.22) | 5945.45 (3724.19-7789.37) | 74.91 (46.54-98.31) | 1.34% (0.89-1.96) | 0.49 (0.47-0.52) |
| Ukraine | 76232.04 (58952.72-99883.59) | 135.43 (104.84-174.63) | 98385.87 (75625.8-127430.84) | 155.92 (121.12-200.19) | 0.29% (0.19-0.4) | 0.38 (0.31-0.46) |
| United Arab Emirates | 131.46 (93.28-178.14) | 94.05 (66.93-127.04) | 826.44 (549.69-1199.94) | 90.02 (60.43-129.24) | 5.29% (3.92-6.86) | -0.26 (-0.61-0.09) |
| United Kingdom | 115879.21 (91330.33-148703.31) | 146.83 (116.07-187.33) | 156916.23 (124013.93-197817.02) | 148.14 (116.72-184.99) | 0.35% (0.26-0.43) | 0.08 (0.02-0.14) |
| United Republic of Tanzania | 5514.41 (3803.46-6882.94) | 83.56 (56.03-105.3) | 12765.5 (8823-16139.4) | 84.74 (58.4-106.72) | 1.31% (0.91-1.77) | -0.06 (-0.15-0.04) |
| United States of America | 385147.8 (297138.53-505262.05) | 140.01 (107.86-182.87) | 749378.66 (587081.93-952929.52) | 162.34 (127.34-203.97) | 0.95% (0.81-1.08) | 1.02 (0.82-1.22) |
| United States Virgin Islands | 51.93 (43.48-61.98) | 97.97 (82.68-115.42) | 160.82 (135.64-191.12) | 115.41 (98.6-135.98) | 2.1% (1.69-2.61) | 0.88 (0.74-1.02) |
| Uruguay | 2993.01 (2254.12-3727.75) | 93.18 (71.29-113.87) | 4451.3 (3471.71-5543.91) | 93.87 (73.79-116.24) | 0.49% (0.4-0.59) | 0.03 (-0.02-0.08) |
| Uzbekistan | 7342.21 (5063.34-10331.69) | 93.72 (65.62-131.65) | 15080.11 (11750.74-19410.29) | 164.01 (134.73-200.8) | 1.05% (0.61-1.5) | 2.32 (2.17-2.47) |
| Vanuatu | 42.95 (31.37-57.7) | 112.2 (82.96-150.11) | 131.84 (99.39-171.77) | 125.63 (95.86-161.32) | 2.07% (1.67-2.55) | 0.38 (0.34-0.42) |
| Venezuela (Bolivarian Republic of) | 4755.6 (3895.95-5928.29) | 75.55 (62.46-92) | 16115.33 (12649.75-20226.98) | 81.03 (64.06-101.17) | 2.39% (1.92-2.97) | 0.02 (-0.06-0.11) |
| Viet Nam | 27341.35 (20470.76-35972.51) | 99.04 (75.74-126.87) | 67464.36 (50995.94-87325.38) | 114.08 (86.71-144.54) | 1.47% (1.13-1.85) | 0.53 (0.48-0.59) |
| Yemen | 2043.15 (1497.91-2696.99) | 73.32 (54.02-96.44) | 6053.13 (4591.31-7860.32) | 77.67 (59.98-99.56) | 1.96% (1.55-2.51) | 0.29 (0.26-0.33) |
| Zambia | 1138.52 (805.68-1467.38) | 71.5 (50.04-92.16) | 3697.28 (2685.19-4518.79) | 99.95 (72.15-122.67) | 2.25% (1.5-3.23) | 1.25 (1.07-1.42) |
| Zimbabwe | 1589.82 (1183.88-2037.35) | 68.1 (51.51-84.9) | 2756.19 (2056.94-3505.4) | 71.95 (54.32-89.59) | 0.73% (0.51-1) | 0.25 (0.17-0.33) |
